# Characteristics and Potential of KSL, KSL-W, and Dadapin-1 Antimicrobial Peptides for Preventing Infections of Orthopedic Prosthetic Devices: Identifying the Most Robust Candidate

**DOI:** 10.3390/ijms26167745

**Published:** 2025-08-11

**Authors:** Davide Campoccia, Andrea De Donno, Giulia Bottau, Gloria Bua, Stefano Ravaioli, Eleonora Capponi, Giovanna Sotgiu, Francesco Pegreffi, Silvia Costantini, Carla Renata Arciola

**Affiliations:** 1 Laboratorio di Patologia delle Infezioni Associate all’Impianto, IRCCS Istituto Ortopedico Rizzoli, Via di Barbiano 1/10, 40136 Bologna, Italy; andrea.dedonno@ior.it (A.D.D.); giulia.bottau@ior.it (G.B.); gloria.bua@ior.it (G.B.); stefano.ravaioli@ior.it (S.R.); eleonora.capponi@ior.it (E.C.); 2 Institute for Organic Synthesis and Photoreactivity (ISOF), National Research Council, Via Gobetti 101, 40129 Bologna, Italy; giovanna.sotgiu@isof.cnr.it; 3 Department of Medicine and Surgery, School of Medicine and Surgery, “Kore” University of Enna, 94100 Enna, Italy; francesco.pegreffi@unikore.it; 4 Unit of Recovery and Functional Rehabilitation, P. Osp. Umberto I, 94100 Enna, Italy; 5 Department of Medical and Surgical Sciences (DIMEC), University of Bologna, Via San Giacomo 14, 40126 Bologna, Italy; silvia.costantini4@studio.unibo.it; 6 Laboratory of Immunorheumatology and Tissue Regeneration, Laboratory of Pathology of Implant Infections, IRCCS Istituto Ortopedico Rizzoli, Via di Barbiano 1/10, 40136 Bologna, Italy

**Keywords:** antimicrobial peptides, AMPs, orthopedic infections, *Staphylococcus*, bacterial biofilm, orthopedics, minimum inhibitory concentration, minimum bactericidal concentration, minimum biofilm inhibitory concentration

## Abstract

Antimicrobial peptides (AMPs) are increasingly emerging as alternatives to conventional antibiotics. This study compared the antibacterial activity of two decapeptides, KSL and KSL-W, and a 23-residue peptide, Dadapin-1, against bacterial species that colonize orthopedic implants, with the aim of identifying the most effective peptide for future AMP-based anti-infective orthopedic biomaterials. *Staphylococcus aureus* ATCC 25923 was the reference strain. The minimum inhibitory concentration (MIC), minimum bactericidal concentration (MBC), and minimum biofilm inhibitory concentration (MBIC) of the AMPs were determined in both undiluted and diluted Mueller–Hinton Broth II (MHB II) to gain a simplified perspective on the potential interference of bioenvironments. The MBICs of the AMPs were close to their MICs. In diluted broth, a concentration of 3.91 μg/mL of KSL or KSL-W was bactericidal against staphylococci and prevented biofilm formation. An eight-fold higher concentration of Dadapin-1 was required to achieve bactericidal activity. Undiluted MHB II significantly hindered the antibacterial activity of KSL and Dadapin-1, while KSL-W was notably less affected. The values of LoA, a newly developed indicator of loss of activity, confirmed these findings. Bacterial species and strain influenced LoA. Furthermore, KSL-W exhibited a protective effect on osteoblasts co-cultured with *S. aureus* ATCC 25923. Overall, KSL-W emerged as the most promising candidate for AMP-based anti-infective orthopedic biomaterials.

## 1. Introduction

Microbial infections remain a worrying, still unresolved problem associated with the use of biomaterials in orthopedics, as well as in other fields of medicine where prostheses and indwelling devices are largely and increasingly used [1]. Perioperative antibiotics have proved capable of preventing a significant proportion of infective events. Nonetheless, multiresistant strains have become frequent, and the spread of antibiotic resistance among bacterial strains represents a serious threat for the prevention and cure of prosthetic infections in orthopedics. Indeed, antibiotic-based prophylaxis measures, such as perioperative antibiotics and anti-infective biomaterials, as well as antibiotic-based treatments, encounter reduced efficacy. Moreover, the majority of opportunistic pathogens causing biomaterial-associated infections, once adhered to implant surfaces, form protective biofilms, become tolerant to common antibiotics, and skew host immune defenses [2]. Current preventive and therapeutic strategies to deal with the phenomenon of implant infections are increasingly focusing on the use of new bioactive molecules that are alternative to current antibiotics. Depending on the clinical application and anatomic site of insertion, they were initially directed towards the development of antifouling surfaces, controlled local delivery of well-characterized antibiotic drugs (for instance, aminoglycosides, glycopeptides such as vancomycin, or combinations of antibiotics such as clindamycin/rifampicin and minocycline/rifampicin), and long-established antibacterial substances (ranging from metal ions, such as Ag^+^, Cu^2+^, and Ga^3+^, and metal nanoparticles to disinfectants such as chlorhexidine and triclosan) [3]. With time, the range of strategies to counteract biomaterial-associated infections has significantly broadened, with scientists starting to contemplate multifront approaches and even considering the possibility of immunomodulating the host response and strengthening the immune defenses against bacterial biofilms [4]. An interesting category of antibacterial molecules that, with time, has drawn growing attention is that of antimicrobial peptides (AMPs) [5]. Initially identified from natural sources, AMPs take part in the innate defense systems of a broad variety of organisms, including plants, fungi, and animals [6]. Most natural AMPs are polycationic peptides capable of interacting with and disrupting the integrity of the cell membranes of fungi, bacteria, and viruses, determining the lysis of the microorganism. However, others exhibit diverse mechanisms of action and different cellular targets [7], such as, for instance, enzymatic activities causing the lysis of the bacterial cell wall. Nonetheless, the enormous possibilities of permuting the sequences of amino acids of the peptides offer infinite combinations and real chances to identify new powerful molecules with selective activity against pathogenic microorganisms. Moreover, the selection of improved peptide sequences helps to minimize some shortcomings of the current AMPs, such as thermal instability, vulnerability to the enzymic action of proteases, or inactivation following interaction/complexation with components of the physiologic fluids or defense mechanisms developed by bacteria. Thus, starting from the known sequences of natural peptides, algorithms can be applied to identify new artificial AMPs that optimize antimicrobial and other biological activities [8,9,10].

In 2021, aiming at identifying AMPs for a possible application in the prevention or treatment of prosthetic orthopedic infections, our group conducted an extensive systematic review on known short-chain AMPs, primarily based on the Database of Antimicrobial Activity and Structure of Peptides (DBAASP) [11]. The choice of considering short-chain peptides was dictated by the potentially lower allergenic risk offered by small peptides. The query included all monomeric AMPs with a length spanning from 7 to 13 amino acids. The research results provided a list of 415 entries, which were then extended by consultations of further information sources (e.g., PubMed, Web of Science and Scholar Google). The top-ranking AMPs emerging from the survey included, among a few others, the new antibiotic teixobactin [12] and the antimicrobial peptide KSL [13,14]. Described for the first time in 2015 [15], teixobactin damages only membranes that contain lipid II, which is absent in eukaryotes, and thus, it does not produce damage to the membranes of mammalian cells [12]. The questionable use of important antibiotics in delivery systems for the prevention of infections, the difficult synthesis of the peptide, and a lower potency of teixobactin against Gram-negative pathogens guided us to instead focus our attention on alternative choices, i.e., the decapeptide KSL [16] and its derivative KSL-W [17], which were considered some of the most interesting AMPs in terms of the scope of our research. KSL was initially discovered from the screening of combinatorial libraries consisting of simplified amino acid sequences for peptides active against the *Candida albicans* membrane [13,14,18]. KSL is a polycationic peptide (net charge of +5) which contains five Lys residues, and the primary target of its bactericidal mechanism is the bacterial membrane. Lipid membrane perturbation by KSL occurs without pore formation. KSL may adopt a β sheet structure on lipid membranes, and this seems to be a critical requirement for its lipid perturbation activity [19]. KSL-W is a KSL analog with a net charge of +4 in which the Lys^6^ residue is replaced by Trp [17] and whose main strength is a greater stability to enzymic activity such as trypsin-catalyzed cleavage in human saliva. Both KSL and its derivative KSL-W have previously been investigated, with studies proving their activity against oral bacteria and supporting their clinical use as antiplaque agents [16,17,18,20,21,22].

The aim of this study was to provide a further, in-depth exploration of the potential of these two decapeptides and of a third, more recently discovered, AMP consisting of 23 amino acids, namely Dadapin-1 [23], as potential candidates for the production of novel anti-infective coatings for resurfacing orthopedic implants. Dadapin-1 was initially developed following a process of simultaneous automatic selection of natural peptides of anuran origin (from the dedicated Database of Anuran Defense Peptides, DADP) and mutation of multiple sequences by means of the Mutator software [23]. The modification strategy consisted in replacing a Val with a Lys residue, which enhanced the net positive charge of the peptide (+5) while decreasing its hemolytic activity. In aqueous solutions, Dadapin-1 exhibits a mostly disordered structure, but it shows a transition to a partly helical structure in the presence of membrane-like environments and has been found to damage bacterial membranes at sub-MICs [23].

**Figure 1:**
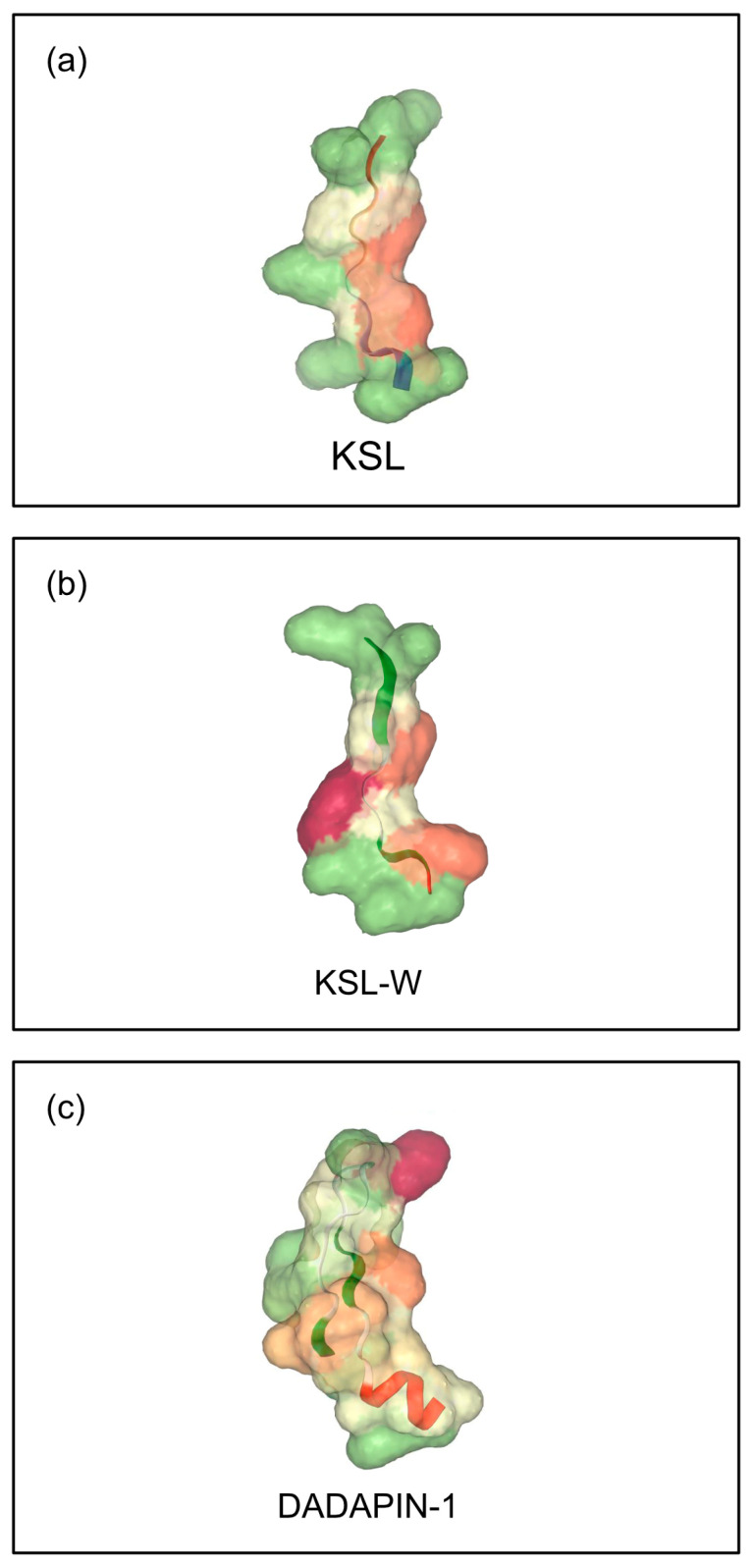
The illustration reports the 3D structure prediction for (**a**) KSL, (**b**) KSL-W, and (**c**) Dadapin-1. The images were obtained by the PEP-FOLD4 tool of the Mobyle platform developed jointly by the Institut Pasteur Biology IT Center and the Resource Parisienne en Bioinformatique Structurale (https://bioserv.rpbs.univ-paris-diderot.fr/services/PEP-FOLD4 accessed on 30 April 2025). Secondary structure and molecular surface hydrophobicity are represented.

Earlier, Dadapin-1 underwent some preliminary investigations [24] and was included in the present work mainly as a useful term of comparison for the two decapeptides. Here, the characterization of Dadapin-1 has been further extended to four new bacterial strains, two of which belong to *Enterococcus faecalis*, a species on which this peptide was never tested before. Figure 1 reports the 3D structure prediction for all three peptides.

To characterize the peptides’ antibacterial properties, microbiological tests were performed using two different culture conditions for the reference *S. aureus* strain, as well as for other bacterial species involved in orthopedic prosthetic infections. Müller–Hinton Broth II (MHB II) is a culture medium known to be optimized for bacterial growth and antibiotic testing. However, different works have highlighted some chemical components of MHB II medium that have been found to form complexes with cationic peptides and quench their antimicrobial activity [25,26,27]. This interference results in higher MIC, MBC, and MBIC values. Taking into consideration this potential interference, the antibacterial activity of the selected AMPs was therefore tested under two distinct culture conditions, i.e., in undiluted and in diluted MHB II [24]. Further, noticing a different susceptibility of the peptides to the medium composition, we tried using a quantitative approach to measure the interference of the culture broth on the AMPs. Intriguingly, the loss of activity of the peptides in undiluted medium depended not only on the specific chemistry of the peptide but also on the bacterial species/strain used in the test.

## 2. Results

### 2.1. Bacterial Ribotyping

In previous circumstances, we stressed the importance of a fine characterization of clinical isolates to be utilized in preclinical experimental investigations for the assessment of anti-infective substances [24]. As part of the qualification process of the clinical isolates, automated ribotyping (riboprinting) by a RiboPrinter^®^ Microbial Characterization System (Qualicon, Wilmington, DE, USA) was performed for robust molecular confirmation of the species identification. Additionally, bacterial riboprinting helped to obtain information on the ribopattern and the specific ribogroup to which each bacterial strain used in the present microbiological investigation belonged. Ribopattern and ribogroup information is reported in Table S1. The pairs of isolates belonging to the same species were shown not to belong to the same ribogroup and, therefore, were confirmed not to be clonal duplicates.

### 2.2. Bacterial Strains’ Antibiotic Resistance

The phenotypic antibiotic resistances expressed by the different clinical isolates are reported in Tables S2–S4. The enlisted strains comprised a methicillin-resistant *S. epidermidis* (MRSE), a multiresistant *E. coli* strain with a MAR of 8, which, across the entire panel of antibiotics screened, exhibited sensitivity just to tigecycline and imipenem, and a multiresistant *S. aureus* strains with a MAR of 9, sensitive just to vancomycin and tigecycline.

### 2.3. MIC and MBC in Undiluted Medium

The antibacterial properties of all test AMPs were investigated under two distinct conditions: 20% and 100% MHB II. It should be noted that the annotations “20%” and “100%” correspond just to the nominal medium concentrations of the bacterial suspension before adding the challenging AMP solution. Table 1 and Table S5 (for μM concentrations) report the MIC and MBC of the three peptides in 100% MHB II.

Where not differently specified, MIC values in the tables refer to measurements performed by ATP luminescence assay. However, in very few circumstances, the MIC could not be determined (ND) either by luminescence or by both luminescence and OD reading. For instance, both *P. aeruginosa* strains exhibited an abnormal increase in luminescence readings in proximity to the MIC value obtained by OD, hindering correct MIC determination for KSL and KSL-W. A similar phenomenon was observed for EF 02. Results from each single experiment performed with the three AMPs can be found in Tables S7–S9.

In undiluted MHB II, KSL-W emerged as the peptide with the greatest antibacterial potency and, thus, with the best performance in such a challenging condition. MIC and MBC values of KSL-W were lower than those of KSL for the vast majority of the strains. Only with *S. epidermidis* did the differences in MIC and MBC for KSL and KSL-W appear attenuated. In Table 1, earlier data concerning Dadapin-1 [24] appear between brackets. They have been integrated with new data from the present study and can be used for comparison with the MIC/MBC values of the two decapeptides. In undiluted medium, the antibacterial activity of Dadapin-1 was found to be marginal, with MICs frequently exceeding the highest concentration of the peptide tested and, anyway, pronouncedly higher than those of KSL and KSL-W, even when expressed in μM concentration (Table S5). MBCs always exceeded 500 μg/mL (Tables S5 and S6). The newly investigated strains of *P. aeruginosa* (PA 02) and *E. faecalis* (EF 01, EF 02) were found to be non-susceptible to Dadapin-1 across the entire range of concentrations tested. Vice versa, the new *S. aureus* (SA 02) exhibited very high MIC and MBC values, consistent with past results obtained with the other *S. aureus* strains [24].

**Figure 2:**
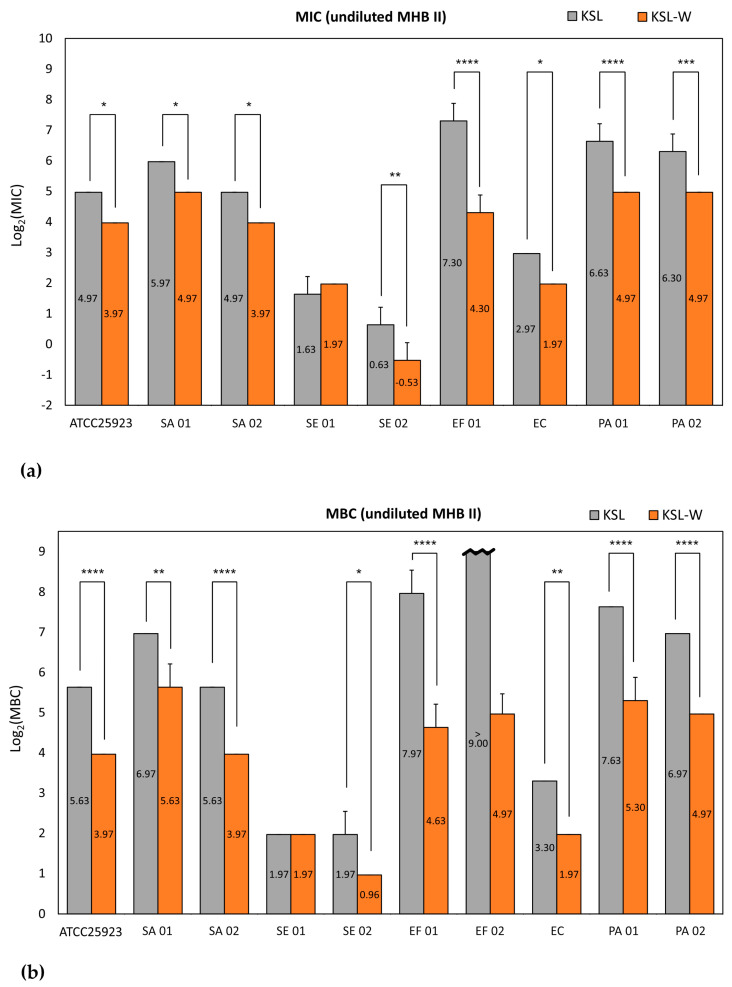
Bar graphs show the antibacterial activity of KSL and KSL-W in terms of MIC (**a**) and MBC (**b**), when tests were performed in undiluted MHB II. MIC and MBC values in μg/mL from each single experiment were Log_2_-normalized. Bars represent the mean value and SD of 3 to 5 independent experiments. For the statistical analyses, Log_2_-normalized data were analyzed by two-way ANOVA followed by post hoc Tukey’s multiple comparisons test. *, *p* < 0.05; **, *p* < 0.005; ***, *p* < 0.0005; ****, *p* < 0.0001.

Statistical analysis of normalized Log_2_ (MIC) of KSL and KSL-W in undiluted (100%) and diluted (20%) MHB II is reported in Tables S10 and S11. Two-way ANOVA followed by Tukey’s multiple comparisons test showed that the MICs of KSL and KSL-W in undiluted medium were always significantly different except in the case of SE 01 (Figure 2a). Under these circumstances, KSL-W clearly confirmed its superiority over KSL. The same was also observed with the MBC (Figure 2b and Tables S12 and S13). For EF 02, the MBC could not be included in the statistical analysis because values exceeded the range of concentrations assayed.

With bacteria cultured in undiluted broth, the observed MBC/MIC ratio for KSL (Table S14) was 1 or 2 in eight bacterial strains. The sole exception, with a ratio between 2 and 4, was observed in the case of SE 02. For EF 02, the MBC exceeded 500 μg/mL and the ratio could not be calculated. In the case of KSL-W, the majority of strains (6 out of 10) exhibited a value of MBC/MIC ratio of 1, suggesting that the minimum inhibitory concentration was bactericidal. A ratio between 1 and 2 or 2 was observed in three strains. Interestingly, as in the case of KSL, even KSL-W showed a ratio of 2–4 in association with SE 02, suggesting that the growth of this clinical isolate could be influenced even at concentrations of polycationic peptides more pronouncedly lower than the MBC. To ensure data uniformity and correct comparison, MBC of Dadapin-1 was re-tested with the new methodological approach used in the present study, i.e., plating 10 μL of suspension and expecting no colony growth. Nonetheless, the MBC/MIC ratio could not be determined as MBC in undiluted medium always exceeded 500 μg/mL.

### 2.4. MIC and MBC in Diluted Medium

When the microbiological tests were conducted in diluted MHB II medium (Table 2 and Tables S15–S19), the performance of all AMPs substantially improved. The observed greater activity of the peptides in diluted medium confirmed the inhibitory effects of some ingredients of MHB II medium on cationic peptides. However, some bacterial strains exhibited poor growth in a broth that is less rich in nutrients, and the luminescence assay often became essential to sensitively detect the MIC. For instance, the limited growth of *E. faecalis* strains in diluted broth hindered a valid determination of MIC values with the less sensitive OD measurements. In the specific case of EF 02, poor cell growth also hindered the efficacy of the luminescence reading, leading to undetermined results by both of the adopted techniques (ND ^L/OD^).

**Figure 3:**
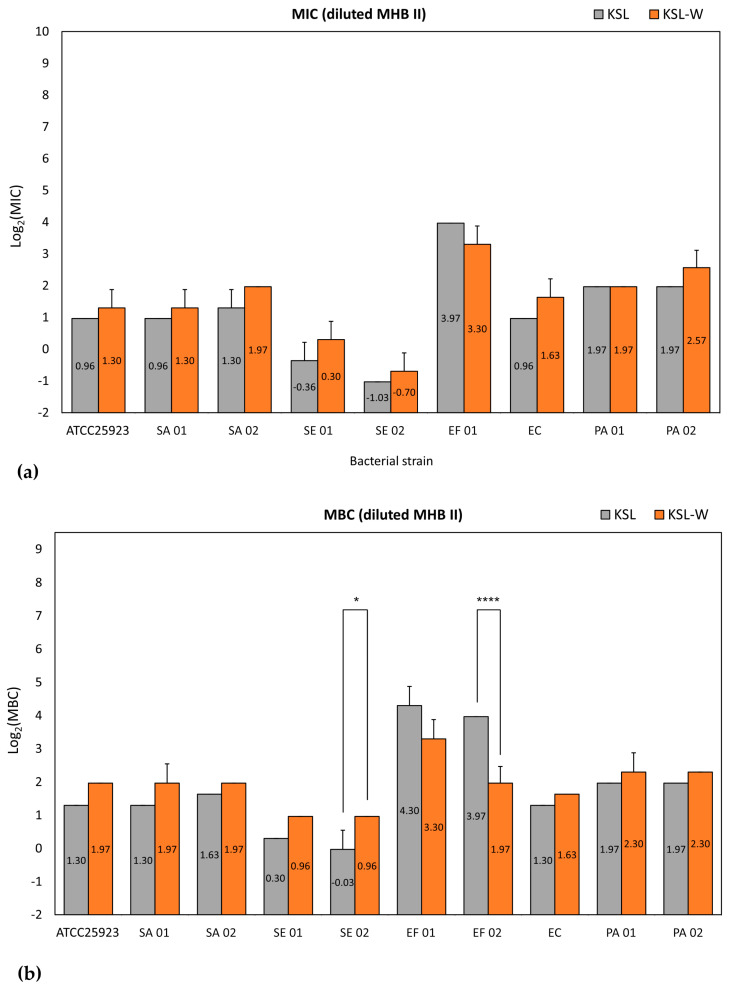
Bar graph showing the antibacterial activity in terms of MIC (**a**) and MBC (**b**) in diluted MHB II. MIC and MBC values in μg/mL from each single experiment were Log_2_-normalized. Bars represent the mean value and SD of 3 to 5 independent experiments. For the statistical analyses, Log_2_-normalized values were analyzed by two-way ANOVA followed by post hoc Tukey’s multiple comparisons test. *, *p* < 0.05; ****, *p* < 0.0001.

Interestingly, under these mutated circumstances, the use of diluted medium revealed a similar performance of KSL and KSL-W (Table 2 and Tables S15–S19), and it seemed as if, in the absence of interference due to the polyanionic component of undiluted broth, KSL-W and KSL possessed comparable antimicrobial activity. This is evident in Figure 3a, which shows that statistical differences were never observed when cross-comparing the MICs of KSL and KSL-W (see also Tables S10 and S11 with the detailed results of the statistical analysis). A similarity emerged even from the analysis of the MBC values, and only two significant differences were reported over the entire panel of strains assayed (Figure 3b). In diluted broth, KSL-W exhibited superior antibacterial properties on EF 02, showing an MBC value of 3.91 μg/mL vs. 15.63 μg/mL for KSL. Conversely, the MBC value of KSL for SE 01 was as low as 0.98 μg/mL vs. 1.95 μg/mL for KSL-W. All the other strains indicated closely resembling MBCs for both KSL and KSL-W (Tables S12 and S13). These findings suggest that undiluted broth masks the true antibacterial potential of KSL and that the real advantage of KSL-W over KSL mostly relies on its more stable bacteriostatic and bactericidal activity, which largely persists even in the presence of the interfering substances of MHB II.

With regard to Dadapin-1, consistently with what was reported earlier [24], the antimicrobial activity substantially improved when tests were performed in diluted broth. The data collected with the new *S. aureus* (SA 02) strain were aligned with data of the previously tested *S. aureus* strains, although this newly investigated strain is characterized by a profile of antibiotic resistance that includes several antibiotic substances. The same applies also to the new *P. aeruginosa* (PA 02) strain, which indicated a MIC perfectly matching with the one found for the other Gram-negative strains (PA 01, but also EC). Of interest, Dadapin-1 had never been tested on *E. faecalis* before. Our results show a remarkable difference in the bactericidal activity of Dadapin-1 on the two enterococcal strains. Unexpectedly, with EF 01 the MBC of Dadapin-1 (in micromolar concentration; see Table S15) was very close to that of both KSL and KSL-W. On the contrary, EF 02 was found to be completely unsusceptible to Dadapin-1 over the range of tested peptide concentrations. It is curious to note that, in the case of KSL-W, EF 02 had an opposite behavior, and it turned out to be much more susceptible to KSL-W than EF 01. Even more so, in the case of KSL, differences between MBC tested on EF 01 and EF 02 were marginal. These observations suggest a completely different interaction of the three polycationic peptides with bacterial strains of the same species. Apart from what was just said regarding *E. faecalis*, even when they were expressed in the form of μM concentration (Table S15), MICs and MBC values of Dadapin-1 were always found to be at least a dilution factor higher than the corresponding ones of KSL and KSL-W.

The MBC/MIC ratios observed with bacteria cultured in diluted MHB II are reported in Table S20. For KSL, a ratio of 1 was observed for three bacterial strains. For six strains, the ratio was 1–2 or 2, and for one strain, the ratio was undetermined. In the case of KSL-W, a ratio of 1 was observed in four strains. Four strains gave a ratio of 1–2, while a ratio of 2–4 was observed just for one strain. EF 02 did not provide a valid MBC/MIC ratio, as was also observed for KSL. For Dadapin-1, five strains showed a ratio of 1 and three exhibited an MBC/MIC ratio of 1–2 or 2. Moreover, in the case of Dadapin-1, even EF 01, in addition to EF 02, did not provide a valid MBC/MIC ratio.

### 2.5. MBIC

The ability of all three AMPs to prevent biofilm formation was assayed in diluted and undiluted medium (Table 3, Tables S21 and S22). After 24 h under static culture conditions, viable adhered bacteria on the plastic surface of the microtiter plate wells were assessed by ATP luminescence assay. With rare exceptions, MBIC values were generally equal or within 1 dilution factor of difference with respect to the corresponding MIC values. Thus, under these test conditions, MBIC was found to generally approximate MIC. Moreover, because of the greater number of bacteria existing in the 24 h biofilms with respect to planktonic cells in suspension, determination of MBIC was successfully achieved even in the case of poorly growing bacteria in diluted medium, for which the MIC was ND.

**Figure 4:**
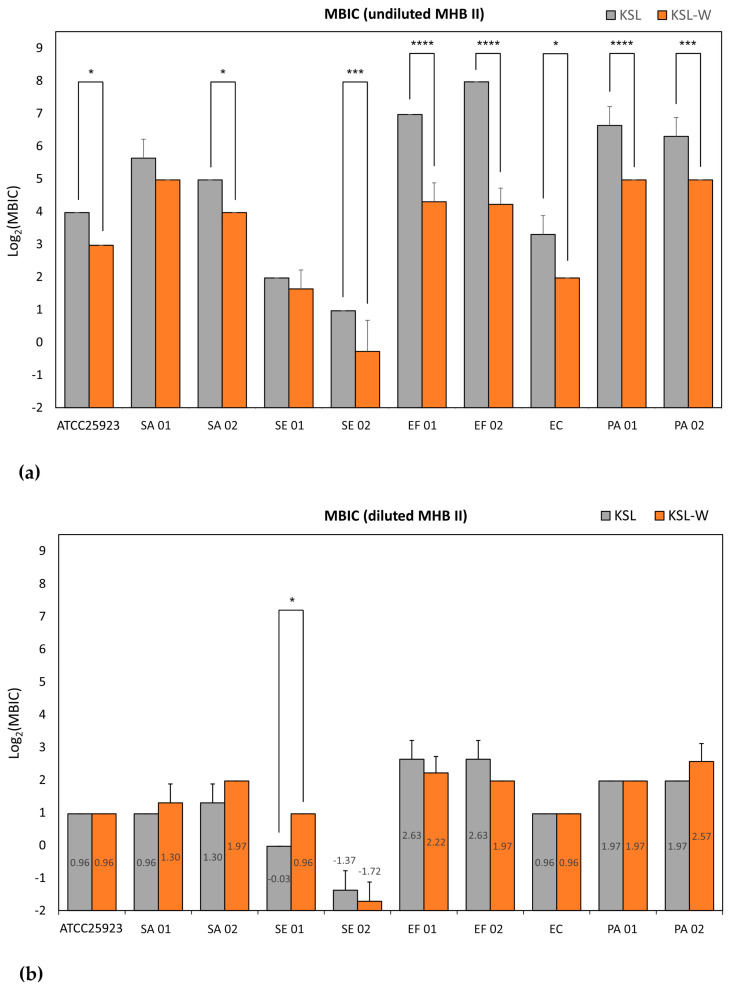
Bar graphs showing the MBIC when tested in (**a**) undiluted and (**b**) diluted MHB II. MBIC values in μg/mL from each single experiment were Log_2_-normalized. Bars represent the mean value and SD of 3 to 5 independent experiments (see in Supplementary Tables S7, S8, S17 and S18). For the statistical analyses, Log_2_-normalized were analyzed by two-way ANOVA followed by post hoc Tukey’s multiple comparisons test. *, *p* < 0.05; ***, *p* < 0.005; ****, *p* < 0.0001.

Figure 4 reports the Log_2_-normalized MBIC mean values found for KSL and KSL-W, in undiluted (Figure 4a) and diluted medium (Figure 4b). The results of the statistical analysis cross-comparing the activity of KSL and KSL-W under both culture conditions can be found in Tables S23 and S24. Once again, it may be noticed that, in undiluted medium, KSL-W confirmed its superior antibacterial activity even in preventing biofilm formation. Only with two strains out of ten, namely SA 01 (p = 0.16) and SE 01 (p = 0.72), the difference observed was not statistically significant. In undiluted medium, KSL and KSL-W manifested a similar antibiofilm activity except with SE 01, for which strain KSL showed a slightly stronger biofilm inhibitory activity than KSL-W. It would appear as if this specific strain was more susceptible to KSL than to KSL-W and, for this reason, the advantage expressed by KSL-W in undiluted broth was attenuated and non-significant. However, it should be recalled that, with this specific strain, the MIC and MBC of the two decapeptides in diluted and undiluted broth always showed similar inhibitory and bactericidal activity. Overall, in diluted medium, 3.91 μg/mL of any of the two decapeptides was effective in inhibiting biofilm formation of all staphylococcal strains tested, *S. epidermidis* being more susceptible than *S. aureus*. The same concentration of KSL could also inhibit the biofilm of all Gram-negative strains. Conversely, 7.81 μg/mL of KSL-W were required to ensure the same effect. *E. coli* was more susceptible than the two *P. aeruginosa* strains, being inhibited already at a dose as low as 1.95 μg/mL of any of the decapeptides. A total of 7.81 μg/mL of KSL and KSL-W ensured the inhibition of both *E. faecalis* strains.

In undiluted medium, *S. epidermidis* remained susceptible to a concentration of 3.91 μg/mL of both peptides, but inhibition of biofilm formation of all staphylococcal strains required 31.25 μg/mL of KSL-W and up to 62.5 μg/mL of KSL. For KSL-W, 31.25 μg/mL more broadly succeeded in inhibiting biofilm formation of any of the bacterial strains, including the two enterococcal strains, while up to 250 μg/mL was required for KSL.

As far as Dadapin-1 is concerned, the present findings indicate that, in diluted broth, a concentration of 31.25 μg/mL was capable of inhibiting biofilm formation by two, non-clonal, strains of *E. faecalis*. With EF 01, the reduction observed was of three logs (99.9%). The activity was, however, totally lost in undiluted medium. The same performance of Dadapin-1 in diluted medium was also proved on PA 02, reaching a three-log inhibition with 31.25 μg/mL of peptide. Differently, a concentration of 15.63 μg/mL (6.22 μM) was sufficient to inhibit the biofilm formation of the new SA 02 strain. Overall, a concentration of 31.25 μg/mL (12.43 μM) of Dadapin-1 ensured the inhibition of biofilm formation of the entire panel of strains. On the contrary, in undiluted medium, the concentration needed for a broad-spectrum biofilm inhibitory activity exceeded 500 μg/mL. It should be noticed that, when considering MBIC in diluted broth in μM concentrations (Table S21), Dadapin-1 exhibited overlapping range of values with those of KSL in the case of both enterococcal strains.

### 2.6. AMPs’ Susceptibility to the Interference of Undiluted MHB II

It is well established that culture conditions have profound effects on the outcomes of antimicrobial activity tests. All the results described above indicate and statistically prove that the structure of peptides plays a substantial role in determining the susceptibility to the interference determined by medium composition. Nonetheless, the extent of quenching of the antibacterial activity was not constant and seemed to consistently vary. To quantitatively measure the impact of broth concentration on the different peptides and explore the influence of each individual bacterial strain, we decided to follow two alternative approaches, proposing two different quantitative indexes: (i) the rate of abatement (RoA), which is easy to calculate but often consists of a range of values, and (ii) the loss of activity (LoA), which represents a log_2_-reduction value (see the description in Materials and Methods) and was more versatile. In the present study, both these indexes were applied to ascertain the impact of medium dilution on the MBC values. MBC was selected because, in a few cases, MIC values were ND. Nevertheless, both RoA and LoA could be applied to MIC and MBIC values as well. The former, RoA, indicates the fold increase in MBC when comparing the conditions of diluted and undiluted broth (the higher the ratio, the greater the effect of broth concentration and the involved abatement of antibacterial activity). The latter, LoA, implicates the log_2_-trasformation of each individual MBC result and the calculation of a mean value. The log_2_-difference in values for undiluted and diluted broth represents in a single exponential value the strength of the quenching effect (see a more detailed explanation in Section 4).

Table 4 reports RoA and LoA for all test peptides and bacterial strains. KSL-W was the most resilient peptide to the quenching effect of the undiluted medium. Its bactericidal activity underwent just a mild interference. This was generally reflected by values of RoA that were always lower than those reported for KSL and Dadapin-1, across all investigated strains. The RoA of KSL ranged from 2–4 (for *S. epidermidis*) to 64 (for both *P. aeruginosa* strains and *S. aureus* SA 01. In other words, *P. aeruginosa* strains and *S. aureus* SA 01 seemed to thrive well in undiluted broth, requiring up to 64-fold higher concentrations of KSL before being killed. RoA of KSL for EF 02 could not be finely defined but was greater than 32 anyway. Conversely, in the case of KSL-W, values of RoA were in a more restricted range, from 1 (for SE 2) up to 16 (for SA 01 and EF 02).

As far as LoA is concerned, Table 4 reports not only single values found per each single bacterial strain, but also the mean LoA ± standard deviation (SD) of all the strains belonging to the same bacterial species. Interestingly, the data introduced in Table 4 can be easily interpreted and are of prompt support when quantitatively assessing the influence of bacterial strains/species in modulating the intensity of the quenching activity due to the medium.

Focusing on the impact of medium interference on KSL-W bactericidal activity, a trend could be observed, where *P. aeruginosa* was associated with the highest LoA value, closely followed by *S. aureus*, and *E. faecalis.* The same three species exhibited the greatest LoA values even in the case of KSL, but, due to a LoA > 5.00 for *E. faecalis* strain EF 02, these species could not be perfectly ranked. Conversely, for both peptides, the lowest LoA values were associated with the two *S. epidermidis* strains and the single strain of *E. coli*, which, not coincidentally, were also the most sensitive ones to the activity of KSL and KSL-W in undiluted broth. While LoA values for the non-clonal strains of *S. epidermidis* and *P. aeruginosa* were, within the same species, relatively close to each other, a greater heterogenicity of LoA was observed for the strains belonging to *S. aureus* and *E. faecalis.* Expecting an even larger variability of LoA when investigating more consistent and representative numbers of strains, it is likely that *P. aeruginosa*, *S. aureus* and *E. faecalis* would result in behaving similarly. On the contrary, a lower loss of activity associated with the two *S. epidermidis* strains, reconfirmed for both KSL and KSL-W, appears to be a more robust indication of a diverging behavior observed with this species and perhaps with *E. coli* as well.

As expected, for a given strain, LoA of KSL was always greater than that of KSL-W, consistently indicating KSL’s greater susceptibility to undiluted MHB II quenching. Overall, these data clearly demonstrate the lower susceptibility of KSL-W to the inhibitory action of the components in the undiluted culture broth. However, they also reveal that RoA/LoA may be influenced to a certain extent even by the bacterial species/strain. This last finding warrants further investigations both to extend the number of analyzed strains per each species but also to understand the reasons behind it. Certainly, RoA remains the most immediate and simplest approach to perceive the impact of specific environmental conditions on the antibacterial performance. The real advantage offered by LoA is that, differing from RoA, it could be conveniently utilized to perform statistical analyses and assess confidence interval.

### 2.7. KSL-W Suppression of S. aureus Cytotoxicity on Osteoblast-like MG63 Cells

**Figure 5:**
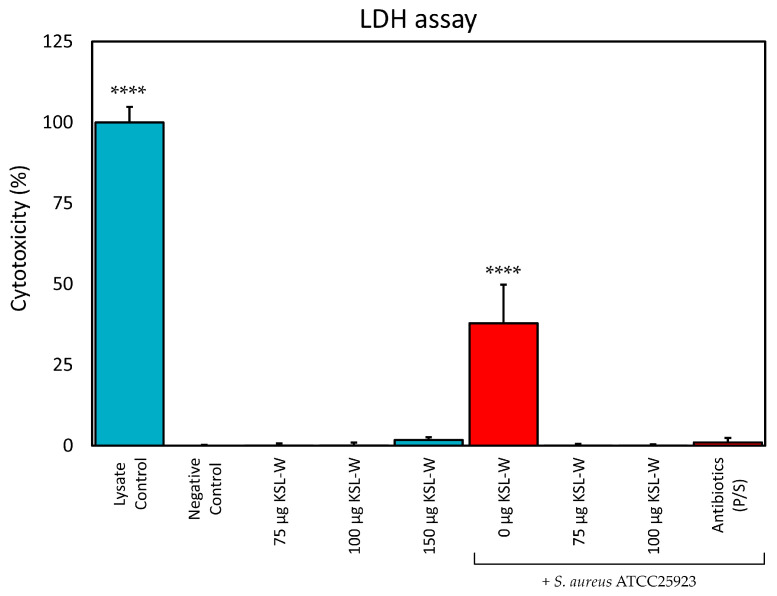
The bar graph illustrates the cytotoxicity as estimated by LDH assay in MG63 cells exposed to the following treatments: cells cultured for 24 h and completely lysed (Lysate Control); cells cultured without any treatment (Negative Control); cells treated for 24 h with different concentrations of KSL-W (the quantity in μg is intended per mL of medium) in the presence (red bar) and in the absence (light blue bar) of bacterial inoculum of *S. aureus* ATCC25923; cells cultured in the presence of inoculum and antibiotics (P/S). It may be noticed as KSL-W did not cause significant cytotoxicity in terms of release of LDH up to a concentration of 150 μg/mL. On the contrary, cells cultured in the presence of *S. aureus*, without additional treatment with KSL-W or antibiotics, significantly differed with respect to all the other treatments. In the presence of *S. aureus*, the viability of osteoblasts was significantly affected. Nonetheless, already at a non-cytotoxic concentration of 75 μg/mL, KSL-W was capable of totally suppressing any cytotoxicity due to the bacterial inoculum. For the statistical analyses, cytotoxicity data were normalized equating the Lysate Control to 100% cytotoxicity. Normalized data in percentage of cytotoxicity were analyzed by one-way ANOVA followed by the Bonferroni/Dunn test. Bars represent the mean ± standard deviation of data from three independent experiments where each treatment was performed in triplicate. Legend: ****; *p* < 0.0001.

KSL-W was selected as the most promising peptide for testing its ability to suppress the cytotoxicity observed when osteoblast-like MG63 cells are co-cultured with *S. aureus*. The cytotoxicity was assessed by means of the LDH assay, which measures the enzymic activity of LDH released in the medium by damaged eukaryotic cells. In preliminary tests, the level of LDH activity in the culture medium proved not to be significantly influenced by the sole presence of the bacterial inoculum of *S. aureus* ATCC 25923. The results are illustrated in Figure 5. It should be noticed that, under the experimental conditions used, KSL-W did not cause significant cytotoxicity up to a concentration of 150 μg/mL. At this peptide concentration, the level of cytotoxicity was still found not to be significantly different with respect to the Negative Control. Conversely, in the presence of *S. aureus*, MG63 cells were severely damaged, leading to the release of LDH. The cytotoxicity caused by the presence of *S. aureus* was estimated to be 37.9% ± 11.9%, significantly differing (*p* < 0.0001) with respect to the Negative Control (0.0% ± 0.3%) and all the other treatments performed (the Lysate Control included). However, when the cells co-cultured with the pathogen were treated with KSL-W, the peptide was capable of completely suppressing any cytotoxicity due to the inoculated bacteria. KSL-W’s efficacy in protecting osteoblast viability started at the lowest concentration of peptide tested (75 μg/mL).

The experimental model also included an additional type of control, which was performed by culturing the cells in the presence of a detergent (1% Tween-20, Positive Control). This control treatment showed a value of cytotoxicity of 50.3% ± 4.2% and significantly differed from all the other treatments (*p* < 0.0001). Positive Control is not included in the histogram in Figure 5 in order to simplify it and make the interpretation of the graph easier. The value observed for Positive Control suggests that, as soon as the cells were treated with the detergent, they were lysed and the LDH enzyme was completely released. A value of 50% approximately corresponds to the expected quantity of cells in culture, before the doubling occurring in the 24 h of incubation that precede LDH testing.

When samples of the supernatants were plated on MHA, bacterial colony growth was never observed in co-cultures that were treated with KSL-W or P/S, suggesting that a rapid bactericidal activity prevented any cell damage due to the inoculated pathogen.

## 3. Discussion

The microbiological properties of three promising antimicrobial peptides, KSL, KSL-W, and Dadapin-1, were extensively analyzed on a panel of relevant and finely characterized clinical bacterial isolates from orthopedic infections. The use of different testing conditions enabled a series of interesting observations. Our findings demonstrate that KSL, KSL-W, and Dadapin-1 have a broad-spectrum antibacterial activity on relevant Gram-positive and Gram-negative bacterial species that are frequently isolated from orthopedic prosthetic infections. Moreover, all test peptides, in particular KSL and KSL-W, turned out to be very active even on multidrug-resistant (MDR) strains, such as SA 02 and EC, which exhibit a high multiplicity of antibiotic resistance (M.A.R. = sum of each single antibiotic resistance) of 9 and 8, respectively. In diluted medium, the differences between the three peptides were subtle, the two decapeptides exhibited equivalent antibacterial activity and all three antimicrobial substances manifested significant antibacterial and bactericidal properties. Conversely, in undiluted medium, the antibacterial activity of the AMPs underwent pronounced variations, with KSL-W emerging as the best performing peptide. In the case of Dadapin-1, the diverging behavior was even more obvious, as its activity was negligible in undiluted medium.

Most known AMPs are amphiphilic polycationic molecules with a positive charge > 3 and whose bactericidal mechanism depends on electrostatic interactions with negatively charged components expressed on the bacterial envelop (e.g., phosphatidylglycerol, lipopolysaccharides and lipoteichoic acids) [16]. Experimental studies have shown that the use of culture media such as MHB may be not ideal for testing cationic peptides. Turner et al. [25] investigated the MIC of two AMPs, i.e., LL-37 and PG-1, against four microorganisms using two diverse culture broth formulations, respectively: standard MHB and a customized broth, which they named “refined” MHB. In the latter formulation, MHB was subjected to anion-exchange chromatography to deplete it of possible (poly)anionic inhibitors. The authors found that MIC determinations performed with standard MHB yielded values that were up to 20-fold higher than those obtained with refined MHB. The major component of MHB is an acid hydrolysate of casein, consisting in a significant proportion of dicarboxylic or phosphorocarboxylic acids (generated by total hydrolysis) and polyanionic peptides (derived from incomplete hydrolysis and composed of negatively charged glutamate and phosphoserine residues). The authors concluded that the use of standard MHB in a NCCLS broth microdilution assay vastly underestimates the activity of LL-37, it is suboptimal for the testing of antimicrobial peptides, and its utility can be improved substantially by anion depletion. The rich component of anionic amino acids would cause precipitation of cationic AMPs, in turn resulting in a pronounced loss of antibacterial activity. More in general, in the presence of certain polyanionic substances (among them even phosphates), electrostatic complexation could take place, sequestering/inactivating part of the free active peptide, and thus markedly diminishing its antibacterial activity [26]. More recently, when the MIC was tested in unmodified standard MHB, a loss of LL-37 activity against Gram-positive and Gram-negative strains was reported also by Mechesso et al. [28]. The authors reported that the antibacterial activity of the peptide was restored when using diluted MHB (12.5%), which roughly corresponds to the real final dilution of MHB II (10%) used in the present study. Curiously, the same effect was not observed with diluted tryptic soy broth (TSB) or Luria−Bertani (LB). These authors finally underscored the usefulness of the diluted broth for antimicrobial screening of peptides.

Certainly, the current knowledge of how and to what extent the many variables of culture conditions are capable of influencing the activity of positively charged peptides is still limited. Among others, the protein source, cationic salts (e.g., NaCl, CaCl_2_, and MgCl_2_), anionic components, and pH are known to play a potential role. In any case, this role varies depending on the molecular structure and the antibacterial mechanism of the specific antimicrobial substance, but also on the microorganism under examination, as also observed in our study. The importance of the use of sensitive conditions and the various environmental conditions capable of influencing the results of antibacterial activity testing have recently been reviewed in [29].

In the present study, we opted to use MHB II as a starting broth, which differs from standard MHB for its balanced cationic formulation. Besides a proportionally reduced concentration of the polyanionic components [25], its use in a ten-time diluted form implies a proportional reduction of cations as well as of the nutrients. Mechesso et al. [28] emphasized the importance of not diluting the media to the extent where bacteria do not grow or differences in bacterial growth are no longer clear. We earlier pinpointed the same limitation rising from the use of diluted broth and excogitated the use of alternative techniques such as the ATP luminescence assay for more sensitive, quantitative and objective measurement of MIC and MBIC [24]. Poor growth was rarely observed. Whenever a reduction in the bacterial growth or an abnormal metabolic activity hindered a correct assessment of the antibacterial activity, the results of the tests were classified as ND.

The structure of the decapeptide KSL-W differs from KSL just for the replacement of the Lys amino acid in sixth position with a Trp residue. This simple substitution was earlier associated with a greater resistance of this peptide to salivary proteases [18]. Our results further indicate that this single substitution in the primary sequence of KSL-W would confer additional desirable properties that have never been reported before as far as we are aware. The minimal loss of activity in undiluted MHB II proves a greater resilience of this peptide to additional factors such as the presence of polyanionic substances, which were found capable of mightily quenching the bacteriostatic, bactericidal, and biofilm-inhibitory activity of the other two polycationic AMPs [23]. These results underscore the strong effect a single amino acid substitution can have on peptide behavior and provide new insights.

Trp is an amino acid with unique physicochemical properties [30] and is capable of both hydrophobic and electrostatic interactions (e.g., cation-π, anion-π, ion pair-π interactions, hydrogen bonds, and hydrophobic contacts) with various molecular components of biological membranes (lipids, glycoconjugates, GAGs, polysaccharides and so on). The role of Trp in membranotropic activity could likely confer to KSL-W an additional functionality with respect to KSL, facilitating its insertion into membranes and its positioning at the membrane–water interface even in conditions where the mechanisms dependable on electrostatic charges are hindered, as in the case of undiluted broth. However, the molecular mechanisms determining the different properties of AMPs under different bioenvironments have still not been completely unveiled. To explain the pronounced LoA of Dadapin-1, whose antibacterial activity in undiluted broth was nearly suppressed, variables are even greater and of more difficult interpretation.

In the attempt to quantitatively express the different susceptibility of KSL-W antibacterial activity to MHB II quenching, we have proposed two types of approaches. LoA emerged as a very versatile measure that warrants further investigation. It potentially enables statistical comparison of the susceptibility of AMPs to interfering substances or physical factors and could be broadly adopted for screening the most stable and suitable candidate peptides. Even further, LoA could also be used to identify and compare the activity of relevant natural molecules that are present in physiologic fluids, which could more critically hinder the efficacy of antimicrobial treatments in the different anatomic sites and interstitial milieux.

It is easily conceivable that AMPs interact differently with the polyanionic components of the culture medium, based on their specific cationic charge and structure. More counterintuitive is that bacterial strains that were found more susceptible to peptides in diluted broth, such as *E. coli* and *S. epidermidis*, were the ones less advantaged in undiluted broth, with MICs undergoing no or limited shift. Indeed, it could be expected that lower concentrations of peptides can more easily be completely sequestered. Regarding the role of the microbial strain, we speculate that the zeta potential of bacterial cells could not only condition the susceptibility to cationic peptides, by attracting a greater number of AMP molecules to the surfaces of more electronegative bacteria but could also generate a competition with the polyanionic components responsible for peptide sequestration. Therefore, the impact of peptide complexation with the polyanionic component of the undiluted broth could be weakened or even abolished by bacterial surfaces with higher affinity for polycationic agents.

Early studies by Hong et al. [13,14,27] assessed the antibacterial activity of KSL on a series of pathogens using antibiotic medium 3 (AM3) (Table S25). Unfortunately, experimental work uniquely concerned testing MIC and not MBC or MBIC. MIC values for *S. aureus* ATCC 6538 and 1550 were, respectively, 3.12 to 22 μg/mL. Thus, they fell in a range lower than that found by us with undiluted MHB II and marginally overlapped with that of diluted MHB II, of 1.95–3.91 μg/mL, which was found for three distinct strains in the present work. In AM3, the MIC on *E. coli* ATCC 2592 ranged from 3.12 to 10 μg/mL, more closely matching our results with EC strain in undiluted broth (7.81 μg/mL), while, with *P. aeruginosa* ATCC 9027, KSL exhibited a MIC of 1.56–5 μg/mL, i.e., very close to that found by us in diluted medium (3.91 μg/mL). More recently, Kosikowska et al. [22] investigated just the MIC of KSL-W in undiluted non-cation-adjusted MHB (Table S26). In the absence of supplemental cations, the MIC of KSL-W was 7.8 μg/mL with *Escherichia coli* PCM 2057, 25 μg/mL with *S. aureus* PCM 2054, 7.8 μg/mL with *S. epidermidis* PCM 2118, and 25 μg/mL with *P. aeruginosa* PCM 499. Thus, MIC values were mostly similar than those found by us with undiluted MHB II, with *E. coli* exhibiting exactly the same value. It is known that even cationic salts in culture broths such as cation-adjusted MHB (MHB II) could potentially interfere with the results of antibacterial activity testing. In fact, they could partly neutralize the negative charges expressed on the outer surfaces of bacteria, having a protective effect. The saturation of the negative charges present on the surface of viable bacteria could compromise the electrostatic interaction between cationic peptides and their anionic targets. However, at least under these circumstances, the supplementation of cations did not appear to cause relevant variations in the results.

The present findings appear promising, particularly in view of our recent data that attest the bland levels of cytotoxicity of these three peptides, which were recently tested on a panel of cell lines including murine fibroblast-like L929 cells, human osteoblast-like MG63 cells and human bone marrow-derived mesenchymal stem cells (hMSCs) [24,31]. As far as KSL-W is concerned, lack of cytotoxicity [16,32,33] or even pro-regenerative properties [34] have been documented also on a series of other primary and secondary cell lines. Furthermore, a Phase 1 and 2a clinical trial recently demonstrated KSL-W’s safety when used as an anti-plaque agent [35]. In the present study, we have provided further evidence that concentrations of KSL-W of up to 150 μg/mL do not cause significant release of LDH and, thus, do not cause cytotoxicity by disrupting membrane integrity of human osteoblast-like cells. Even further, our findings indicate that, although in the presence of serum proteases, KSL-W at a concentration as low as 75 μg/mL totally abolished the toxicity of *S. aureus* in co-culture with MG63 cells, resulting selectively toxic and bactericidal for *S. aureus*.

Along with natural amino acid substitution as the one described for KSL-W, new advancing strategies are being implemented to address the intrinsic limitations of stability and selectivity of peptides. These strategies involve the possibility to empower short peptides activity and stability through various types of chemical modifications such as peptide cyclization [36], lipidation/alkylation [37], nanoparticle conjugation, substitution of natural L-amino acids with synthetic D-amino acids, and peptidomimetics [38].

We do not want to ignore the suggestion that comes from other articles, which is to look not only for single successful AMPs but also for cocktails of AMPs or combinations of AMPs and other antimicrobials. In this regard, Maron et al. studied the effects against *Staphylococcus aureus* of single AMPs, pairs of AMPs, and a library of antimicrobial peptides. The authors found that the treatment with combined pairs of AMPs hindered the evolution of resistance compared to treatments based on single AMPs and that the lowest level of resistance was observed with a random antimicrobial peptide mixture (RPM) that contained more than one million different peptides [39]. Antunes et al. [40] found that RPMs reduce the risk of resistance in *P. aeruginosa* more than single AMPs [41]. Bauer et al. [41], using an RPM entrapped in a copper metal matrix, found an increased antimicrobial activity toward methicillin-resistant *S. aureus* than either copper or RPM alone.

Nanotechnologies lend themselves to enhancing the properties of AMPs. The encapsulation of AMPs in nanoparticles could improve their stability and protect them from protease degradation in the physiological environments [42]. Moreover, functionalizing nanoparticles with AMPs could enable the development of antimicrobial coatings for biomaterials [43,44].

Two very recent and interesting papers focused on the AMP–protease interaction. Kim et al. presented two novel-designed AMPs, DAP-7 and DAP-10, which are able to remain stable for up to 12 h after exposure to proteases [45], while Zhu et al. focused on protease inhibitors as molecular scaffolds in the creation of highly stable peptide-based biomaterials [46].

In this study, an original model including bone cells is proposed to better mimic the complexity of the in vivo environment, with potential future expansion to incorporate immune system components. Key advantages of this approach are the following: a more accurate simulation of the physiological environment by including serum proteins and tissue cells usually absent in standard tests; simultaneous assessment of antibacterial activity and cytotoxicity, verifying bactericidal effects without damage to human cells; and insights into the peptide’s ability to protect human cells from damage caused by pathogens or their toxins.

## 4. Materials and Methods

### 4.1. AMPs

The synthetic peptides KSL (aminoacidic sequence: KKVVFKVKFK), KSL-W (aminoacidic sequence: KKVVFWVKFK), and Dadapin-1, consisting of 23 amino acids (aminoacidic sequence: GLLRASSKWGRKYYVDLAGCAKA), were purchased from GenScript (Shanghai, China) and from Proteogenix Sas (Schiltigheim, France) with a degree of purity > 98%. Mass spectra and HPLC chromatograms of all the different lots of peptides used for the experimental work are reported at the end of the document in the Supplementary Materials (Section: Spectra and chromatograms of the different lots of peptides). Upon arrival, the peptides were placed at −80 °C for long-term storage. For the experimental work, the peptides were diluted to a concentration of 1 mg/mL in sterile deionized water, as recommended by the producers. Peptide aliquots were stored at −80 °C and, once thawed, entirely used within a day.

### 4.2. Strains

The antibacterial properties of the antimicrobial peptides (AMPs) were evaluated using the commercial reference strain *S*. *aureus* ATCC 25923 Seattle 1945, a human clinical isolate (ATCC^®^ Catalog No. 25923™), simply referred to as 25923 or SA 00, along with additional strains representing the most common Gram-positive and Gram-negative pathogens involved in implant-related orthopedic infections: *S*. *aureus* (cra4030 [23] and cra2198), hereafter referred to as SA 01 and SA 02, respectively; *S*. *epidermidis* (cra4034 [23,31] and cra4029 [23]), referred to as SE 01 and SE 02, respectively; *E*. *coli* (cra4038 [23,31]), referred to as EC; *P*. *aeruginosa* (cra4004 [23] and cra3840 [31]), referred to as PA 01 and PA 02, respectively; and *E*. *faecalis* (cra4024 and cra4033), referred to as EF 01 and EF 02, respectively.

To assess the potential of the AMP KSL-W in suppressing the cytotoxic effects of *S. aureus* on osteoblasts, the *S. aureus* ATCC 25923 strain and osteoblast-like MG63 cells (ATCC, Rockville, MD, USA) were utilized.

### 4.3. Bacterial Strain Ribotyping

Each bacterial strain was thawed from frozen stock and seeded on Mueller–Hinton agar (MHA, PO50007A, ThermoScientific, Thermo Fisher Scientific, Segrate, Italy) plates for 24 h at 37 °C. Pure colonies on the MHA were resuspended and processed by RiboPrinter^®^ microbial characterization system (Qualicon, Wilmington, DE, USA), following the manufacturer’s instructions. EcoRI was the restriction enzyme used for the analysis.

### 4.4. Antibiotic Resistance Characterization of Bacterial Strains

All clinical isolates were assayed to determine their resistance to a panel of antibiotic substances by using the technique of the MIC test strips (MTSs) on MHA. MTSs were purchased from Liofilchem (Roseto degli Abruzzi, Italy) and were as follows: gentamicin 0.016–256 μg/mL; imipenem 0.002–32 μg/mL; oxacillin 0.016–256 μg/mL; amoxicillin 0.016–256 μg/mL; ciprofloxacin 0.002–32 μg/mL; vancomycin 0.016–256 μg/mL; clindamycin 0.016–256 μg/mL; penicillin G 0.016–256 μg/mL; penicillin G 0.002–32 μg/mL; piperacillin 0.016–256 μg/mL; tobramycin 0.016–256 μg/mL; trimethoprim*—sulfamethoxazole (1/19) 0.002–32* μg/mL; ceftazidime 0.016–256 μg/mL; ceftobiprole 0.002–32 μg/mL; and tigecycline 0.016–256 μg/mL. Once the MIC values had been obtained, EUCAST breakpoints (“The European Committee on Antimicrobial Susceptibility Testing. Breakpoint tables for interpretation of MICs and zone diameters, version 10.0, 2020”) were applied to classify bacterial strains as clinically resistant/intermediate/sensitive.

### 4.5. Characterization of the Antibacterial Properties of AMPs

**Figure 6:**
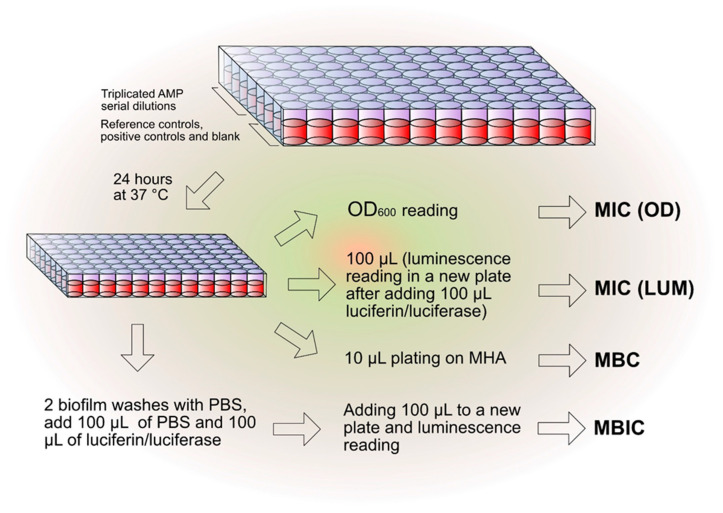
This scheme was generally adopted to assess the antibacterial activity of the peptides, enabling us to obtain two distinct estimations of MIC (by OD and by luminescence measurement), the assessment of the MBC, and the MBIC from a single initial microplate.

The experimental scheme reported in Figure 6 helped to simplify and optimize the in vitro testing of the antibacterial activity of the different AMPs. The initial transparent microtiter plate for the determination of the minimum inhibitory concentration (MIC) by optical density (OD) measurement was conveniently used as a starting point not only for the alterative determination of the MIC by luminescence but also for the minimum bactericidal concentration (MBC) and for the minimum biofilm inhibitory concentration (MBIC), as explained below. For every test peptide, at least three independent experiments were performed on each bacterial strain per test condition (100% MHB and 20% MHB). The sole exception to this rule was when MIC values were on rare occasions found to be greater than 500 µg/mL. Under these circumstances, two consecutive experiments were performed to confirm the results. In this case, the antibacterial activity was considered insignificant and the third experiment was generally considered unnecessary.

### 4.6. Minimum Inhibitory Concentration (MIC)

The antibacterial properties of the test AMPs were assayed under two distinct conditions of bacterial culture: (i) with bacteria cultured in undiluted Mueller–Hinton II Broth (MHB II) and (ii) with bacteria cultured in diluted MHB II as earlier described in Campoccia et al. [24]. Briefly, a few colonies of each bacterial strain, taken from overnight cultures on Mueller–Hinton Agar plates (MHA, PO50007A, ThermoScientific, Thermo Fisher Scientific, Segrate, Italy), were resuspended in Mueller–Hinton II Broth (MHB II, 24107, Liofilchem, Roseto degli Abruzzi, Italy), either undiluted or at a 20% dilution in deionized water. The concentration of bacteria was adjusted to approximately 10^8^ CFU/mL, as estimated by optical density reading at 625 nm, using a Hewlett Packard G1103A spectrophotometer (Waldbronn, Germany). The bacterial suspension was further diluted 1:100 in 100% or 20% MHB II.

Serial 1:2 dilutions of all the test peptides were prepared in sterile deionized water starting from a stock solution of 1 mg/mL. Microtiter plates (96-Well CytoOne^®^ Plate, CC7672-7596, Starlab S.r.l., Milano, Italy) were prepared by adding a volume of 100 µL of inoculum to 100 µL of treatment. Each AMP dilution was assayed in triplicate; each microplate included triplicate wells for the blank (100 µL of sterile distilled water and 100 µL of sterile MHB II), and 3 to 6 wells for reference control of 100% growth (100 µL of sterile deionized water and 100 µL of MHB II inoculated with the bacterium). Positive controls were performed in triplicate wells with 100 µL of a 10 mg/mL gentamicin solution (G1272, Sigma Aldrich, Milan, Italy) and with 100 µL of a solution containing 10,000 units/mL of penicillin and 10,000 µg/mL of streptomycin (penicillin–streptomycin, 15140-122, Life Technologies, Monza, Italy).

After a 1-day incubation at 37 °C, the MIC was determined by two distinct methods: (1) optical density measurement and (2) bioluminescence reading. In the first case, the optical density of the microtiter plates was read directly at a wavelength of 600 nm and the MIC was estimated from the curves obtained by plotting the OD measurements. For the bioluminescence reading, after gentle resuspension, 100 µL of each treated culture was transferred to a new 96-well non-treated plate (236105/237105, Life Technologies, Monza, Italy) and the metabolic activity was determined by the kit Bac-Titer Glo Microbial Cell Viability Assay (G8231, Promega Italia S.r.l, Milan, Italy). Optical density and bioluminescence were read by a Modulus II multifunction plate reader (Turner BioSystems, Sunnyvale, CA, USA) or, alternatively, by an Infinite M Plex (Tecan Group Ltd., Männedorf, Switzerland). As earlier described in [24], in the case of luminescence, the MIC was identified from the curves of growth inhibition as the first concentration leading to a 1-log reduction in bacterial growth (i.e., 90% growth inhibition). A close match was generally observed between MIC estimated by optical reading and luminescence. Nonetheless, when using 20% MHB II, the growth of some bacterial strains was exceptionally low, and OD reading was not as sensitive as luminescence in detecting the bacterial growth. In rare occasions, even the curve obtained by luminescence reading did not allow the extrapolation of valid MIC values. For instance, in the experiments performed with undiluted medium, both strains of *P. aeruginosa* showed an unexpected burst in metabolic activity just in proximity of the MIC value assessed by OD reading.

### 4.7. Minimum Bactericidal Concentration (MBC)

For assessing the MBC, 10 μL of bacterial suspensions was taken from wells corresponding to MIC, 2× MIC, and 4× MIC and then plated on MHA plates. After one day of incubation at 37 °C, the agar plates were examined for bacterial growth. The MBC was determined as the lowest dilution causing absence of bacterial colonies growth on the plate.

### 4.8. Minimum Biofilm Inhibitory Concentration (MBIC)

The wells of the microplate, initially used for the determination of the MIC, were rinsed twice with 220 µL of phosphate-buffered solution (PBS) to remove loosely attached and planktonic bacteria. A total of 100 µL of fresh PBS and 100 µL of Bac-Titer Glo solution (G8231, Promega Italia S.r.l., Milan, Italy) were added to each well. After a 10 min incubation, a volume of 100 µL was transferred to a new microplate for luminescence determination as described above. As a general rule, the MBIC value was identified as the concentration at which a reduction of at least a log in the metabolic activity was observed.

### 4.9. AMPs Susceptibility to the Interference by MHB II

In order to assess the extent of the interference of the culture medium composition with the antibacterial activity of the three peptides, a “Rate of Abatement” of the bactericidal properties (RoA) was calculated using the following formula (Equation (1)):

RoA = MBC_MHB_/MBC_20% MHB_ (1)

RoA indicates the fold increases in the MBC when tested in undiluted medium (MBC_20% MHB_), i.e., with maximal interference of broth polyanionic substances, with respect to diluted medium (MBC_MHB_). A limitation of the rate of abatement is that, under some circumstances, the MBC does not correspond to a single value but rather to a range of values, thus RoA must be expressed as a range of values as well. A more versatile measure to express the loss of activity was calculated as follows. MBC values from each single test were Log_2_-transformed and the mean of the three to five values obtained from the replicated experiments was calculated per each peptide, per each strain, and per each condition (leading to a single Log_2_ (MBC) mean value). The loss of activity (LoA) was therefore measured in terms of Log_2_-reduction with the following formula (Equation (2)):

LoA (MBC) = Mean of Log_2_(MBC_MHB_) − Mean of Log_2_(MBC_20% MHB_) (2)

In case of no MBC variation between undiluted and diluted medium, LoA assumes the value of 0 (i.e., no loss of activity), while a LoA of 1 corresponds to a doubled MBC in undiluted medium (as easily calculated by the formula 2^LoA^).

### 4.10. Cell Culture of Osteoblast-like MG63 Cells

MG63 cells (ATCC, Rockville, MD, USA) were thawed from a frozen stock and cultured in DMEM (11885-084, Thermo Fisher Scientific, Segrate, Italy) supplemented with non-inactivated 10% fetal bovine serum (FBS, F7524, Sigma-Aldrich, Milan, Italy) and 100× Penicillin/Streptomycin solution (P/S, 10,000 units penicillin and 10 mg streptomycin/mL, P4333, Sigma-Aldrich, Milan, Italy), under standard culture conditions (i.e., at 37 °C, in a humidified atmosphere with 5% CO_2_). Cells were regularly subcultured 2–3 times a week by using TrypLE Select 1× (GIBCO, Thermo Fisher Scientific, Segrate, Italy).

### 4.11. Assessment of KSL-W Suppression of S. aureus Cytotoxicity

KSL-W was the peptide selected to investigate the efficacy in suppressing the cytotoxicity of *S. aureus* ATCC 25923 on osteoblast-like MG63 cells. *S. aureus* ATCC 25923 was thawed from the frozen stock and plated on MHA and incubated at 37 °C for 24 h.

A suspension of MG63 cells was prepared in complete DMEM at a concentration of 5 × 10^4^ cells/mL. A total of 100 µL of cell suspension was added to each well of a 96-well cell culture-treated plate (137101, Nunclon Delta Black Microwell, ThermoScientific, Thermo Fisher Scientific, Segrate, Italy). After cell seeding, the plate was incubated for a day at 37 °C under standard culture conditions. A single colony of *S. aureus* ATCC 25923 was taken from the MHA plate and inoculated in 9 mL of Tryptic Soy Broth (TSB, 24513, Liofilchem, Roseto degli Abruzzi, Italy). The inoculated broth was then incubated at 37 °C overnight.

The following day, the cultured broth was centrifuged at 4000 rpm for 15 min using a Biofuge Fresco (Heraeus Instruments, Hanau, Germany) and the pellet was resuspended in DMEM. The new bacterial suspension was sonicated for 10 min and diluted to obtain an optical density of 0.1 at 625 nm. A further 1:50 dilution was performed using DMEM. Before adding the bacterial inoculum to the MG63 cell, the cell culture medium was removed from the wells of the 96-well plate and replaced with 50 µL of fresh DMEM with 10% FBS but without antibiotics. Then, the following treatments were added to the wells cultured with MG63 cells: 50 µL of DMEM w/o FBS and w/o antibiotics (Negative Control and Lysate Control); 50 µL of DMEM containing 2% Tween 20 (P7949, Merck KGaA, Darmstadt, Germany) (Positive Control); 25 µL DMEM + 25 µL of a solution of 600 µg/mL of KSL-W in DMEM (150 µg/mL KSL-W); 25 µL DMEM + 25 µL of a solution of 400 µg/mL of KSL-W in DMEM (100 µg/mL KSL-W); 25 µL DMEM + 25 µL of a solution of 300 µg/mL of KSL-W in DMEM (75 µg/mL KSL-W); 25 µL of bacterial inoculum + 25 µL of DMEM (0 µg/mL KSL-W + *S. aureus*); 25 µL of bacterial inoculum + 25 µL of a solution of 300 µg/mL of KSL-W in DMEM (75 µg/mL KSL-W + *S. aureus*); 25 µL of bacterial inoculum + 25 µL of a solution of 400 µg/mL of KSL-W in DMEM (100 µg/mL KSL-W + *S. aureus*); 25 µL of bacterial inoculum + 25 µL of DMEM with 4× concentration of P/S (Antibiotics + *S. aureus*). The plate was incubated at 37 °C under standard culture conditions for 24 h. All treatments were performed in triplicate wells.

Cytotoxicity was assessed by measuring the release of lactate dehydrogenase (LDH) from damaged cells by the LDH Cytotoxicity Assay (ab197004, Abcam, Cambridge, UK), strictly following the instructions of the manufacturer. For all treatments, each well was assayed in duplicate by transferring 5 µL aliquots of the supernatants (5.5 µL in the case of the Lysate Control as per manufacturer’s indications) to the wells of a new 96-well white plate with clear bottom (165306, Thermo Fisher Scientific, Rochester, NY, USA). Then, 95 μL of working reagent was added to each well. After 10 min shaking, the plate was read in fluorescence (excitation: 535 nm; emission 587 nm) by an Infinite 200 PRO mod. M PLEX multifunction plate reader (Tecan Group Ltd., Männedorf, Switzerland). The experiments were independently performed three times. Furthermore, the bactericidal effectiveness of KSL-W treatment on co-cultures was verified by plating 10 μL of the culture medium on MHA and incubating the agar plates for a day at 37 °C.

### 4.12. Statistical Analysis

For the statistical analysis of the differences between AMPs under the different testing conditions, MIC, MBC, and MBIC values of each single experiment were Log_2_-normalized (see [47]). Differences in Log_2_-normalized data were analyzed by two-way ANOVA followed by post hoc Tukey’s multiple comparisons test using the statistical software GraphPad (ver. 10.4). Values obtained for Dadapin-1, especially in undiluted medium, generally exhibited consistent differences with respect to KSL and KSL-W and did not require statistical analysis.

Data in relative fluorescence units (RFU) from the investigation on KSL-W suppression of *S. aureus* cytotoxicity were processed as follows. The mean of the duplicate readings from each single treated well was calculated. Within each plate, the mean of the Lysate Control was equated to 100% cytotoxicity and all measurements were expressed as percentage of the Lysate Control. After this transformation, the statistical difference between treatments was analyzed by one-way ANOVA, followed by Bonferroni/Dunn test (significance level: 5%) using StatView (version 5.0.1, Sas Institute Inc., Cary, NC, USA).

## 5. Conclusions

The results of the present study demonstrate that all three investigated cationic peptides, KSL, KSL-W, and Dadapin-1, possess significant broad-spectrum antibacterial properties against clinical isolates involved in orthopedic bacterial infections, also including difficult-to-treat MDR strains.

The antimicrobial activity of the three AMPs markedly differed depending on the testing conditions. When AMPs were challenged in undiluted medium, KSL-W was the most stable peptide. The overall characteristics of KSL-W lead us to consider KSL-W the most robust peptide.

Our work can represent a small first step forward in the search for valuable single peptides, with a focus on specific applications, in our case within the orthopedic clinical context, while not neglecting a glance at the emerging potential of mixtures of (random or, in our view, preferably preselected) AMPs or AMPs combined with protective factors (such as nanoparticles or antiproteases).

## Figures and Tables

**Table 1:** Antibacterial activity of AMPs in undiluted MHB II medium.

100% MHB II						
Strain	MIC [μg/mL]			MBC [μg/mL]		
	KSL	KSL-W	Dadapin-1	KSL	KSL-W	Dadapin-1
SA 00	31.25	15.63	500 (>500)	31.25–62.5	15.63	(>500)
SA 01	62.5	31.25	(>500)	125	31.25–62.5	(>500)
SA 02	31.25	15.63	250–>500	31.25–62.5	15.63	>500
SE 01	1.95–3.91	3.91	500 (250)	3.91	3.91	(>500)
SE 02	0.98–1.95	0.49–0.98	>500 (250)	3.91	1.95	(>500)
EF 01	125–250	15.63–31.25	>500	250	15.63–31.25	>500
EF 02	ND ^OD/L^	15.63–31.25	>500	>500	31.25–62.5	>500
EC	7.81	3.91	(>500)	7.81–15.63	3.91	(>500)
PA 01	ND ^L^ 62.5–125 ^OD^	ND ^L^ 31.25 ^OD^	(>500)	125–250	31.25–62.5	(>500)
PA 02	ND ^L^ 62.5–125 ^OD^	ND ^L^ 31.25 ^OD^	>500	62.5–250	31.25	>500

When not differently specified, MIC values refer to results obtained by luminescence. For Dadapin-1, values between brackets indicate earlier published results [24]. For the earlier published data of Dadapin-1, MBC values were tested by plating 100 μL of culture medium instead of 10 μL. Legend: ^L^, value determined by ATP luminescence assay; ^OD^, value determined by optical density reading; ND, MIC could not be determined; green cells: lowest MIC and MBC values observed for a given bacterial strain across the different peptides; gray cells: same MIC or MBC values (or overlapping ranges of values); orange cells: highest MIC and MBC observed values.

**Table 2:** Antibacterial activity of AMPs in diluted MHB II medium.

20% MHB II						
Strain	MIC [μg/mL]			MBC [μg/mL]		
	KSL	KSL-W	Dadapin-1	KSL	KSL-W	Dadapin-1
SA 00	1.95	1.95–3.91	15.63 (7.81)	1.95–3.91	3.91	(15.63–31.25)
SA 02	1.95	1.95–3.91	(15.63)	1.95–3.91	3.91	(15.63)
SA 03	1.95–3.91	3.91	15.63	1.95–3.91	3.91	15.63
SE 01	0.49–0.98	0.98–1.95	3.91–7.81 (7.81)	0.98–1.95	1.95	(7.81)
SE 02	0.49	0.49–0.98	(7.81)	0.98	1.95	15.63 (31.25)
EF 01	15.63	7.81–15.63	ND ^L/OD^	15.63–31.25	7.81–15.63	31.25
EF 02	ND ^L/OD^	ND ^L/OD^	ND ^L/OD^	15.63	3.91	>500
EC	1.95 ^L/OD^	1.95–3.91 ^L/OD^	(31.25)	1.95–3.91	1.95–3.91	31.25–62.5 (31.25)
PA 01	3.91^L/OD^	3.91 ^L/OD^	(31.25)	3.91	3.91–7.81	(31.25)
PA 02	3.91 ^L/OD^	3.91–7.81 ^L/OD^	31.25	3.91	3.91–7.81	31.25

For Dadapin-1, values between brackets indicate earlier published results [24] and new data are introduced only when differing. For MBC, differences could probably derive from the different methodological approach. Legend: ^L^, value determined by ATP luminescence assay; ^OD^, value determined by optical density reading; ND, MIC could not be determined; green cells: lowest MIC and MBC values observed for a given bacterial strain across the different peptides; gray cells: same MIC or MBC values (or overlapping ranges of values); orange cells: highest MIC and MBC observed values.

**Table 3:** Biofilm-inhibitory activity of AMPs (MBIC).

MBIC [μg/mL]						
Strain	Diluted MHB II			Undiluted MHB II		
	KSL	KSL-W	Dadapin-1	KSL	KSL-W	Dadapin-1
SA 00	1.95	1.95	(7.81)	15.63	7.81	250–500 (500)
SA 02	2LR: 1.95	1.95–3.91 4LR: 3.91	7.81–15.63 (15.63)	31.25–62.5 2LR: 62.5	3LR: 31.25	(>500)
SA 03	1.95–3.91 3LR: 3.91	3LR: 3.91	7.81–15.63	2LR: 31.25	3LR: 15.63	500
SE 01	2LR: 0.98	3LR: 1.95	3.91–7.81 (7.81)	3LR: 3.91	1.95–3.91	(500)
SE 02	0.24–0.49	0.24–0.49	(7.81)	2LR: 1.95	0.49–1.95 2LR: 1.95	>500 (250)
EF 01	3.91–7.81	3.91–7.81	15.63–31.25 3LR: 31.25	125	15.63–31.25 3LR: 31.25	>500
EF 02	3.91–7.81 2LR: 7.81	2LR: 3.91	15.63–31.25	250	15.63–31.25 2LR: 31.25	>500
EC	3LR: 1.95	1.95 3LR: 3.91	15.63–31.25 (31.25) 2LR: 31.25	3LR: 7.81	3LR: 3.91	(>500)
PA 01	3LR: 3.91	2LR: 3.91	3LR: (31.25)	62.5–125	2LR: 31.25	(>500)
PA 02	2LR: 3.91	3.91–7.81 2LR: 7.81	15.6–31.25 3LR: 31.25	62.5–125 3LR: 125	3LR: 31.25	>500

MBIC values (in μg/mL) obtained in diluted and undiluted medium. For Dadapin-1 values between brackets indicate earlier published results [24] and new results from the present investigation are displayed only when differing; Legend: LR, Log_10_ reduction in bacterial metabolism for a given MBIC. Green cells indicate the lowest MIC and MBC values observed for a given bacterial strain across the three peptides. Gray cells indicate the same MIC or MBC values (or partially overlapping ranges of values) for a given bacterial strain across the different peptides. Orange cells indicate the highest MIC and MBC.

**Table 4:** Rate of abatement and loss of activity associated with undiluted MHB II.

Bacteria		RoA = (MBC_MHB II_)/(MBC_20% MHB II_)			LoA = Mean of Log_2_(MBC_MHB II_) − Log_2_(Mean of MBC_20% MHB II_)					
		KSL	KSL-W	Dadapin-1	KSL	KSL-W	Dadapin-1	KSL (Mean ± SD)	KSL-W (Mean ± SD)	Dadapin-1 (Mean ± SD)
*S. aureus*	SA 00	16	4	>16	4.33	2.00	>4.33	4.67 ± 0.88	2.55 ± 0.96	>4.78
	SA 02	32–64	8–16	>32	5.67	3.67	>5.00			
	SA 03	16	4	>32	4.00	2.00	>5.00			
*S. epidermidis*	SE 01	2–4	2	>64	1.67	1.00	>6.00	1.83 ± 0.23	0.50 ± 0.71	>5.30
	SE 02	4	1	>16	2.00	0.00	>4.60			
*E. faecalis*	EF 01	8–16	2	>16	3.67	1.33	>4.00	>4.33	2.17 ± 1.18	>4.0
	EF 02	>32	8–16	-	>5.00	3.00	-			
*E. coli*	EC	4	1–2	>16	2.00	0.33	>4.00	2	0.33	>4.0
*P. aeruginosa*	PA 01	32–64	8	>16	5.67	3.00	>4.00	5.33 ± 0.47	2.83 ± 0.24	>4.0
	PA 02	16–64	4–8	>16	5.00	2.67	>4.00			

## Data Availability

The experimental data are presented in detail in the Supplementary Materials.

## Supplementary Materials

The following supporting information can be downloaded at https://www.mdpi.com/article/10.3390/ijms26167745/s1.
