# Supplementary material for: Characteristics and Potential of KSL, KSL-W, and Dadapin-1 Antimicrobial Peptides for Preventing Infections of Orthopedic Prosthetic Devices: Identifying the Most Robust Candidate
Source: Int J Mol Sci. 2025 Aug 11;26(16):7745. doi: 10.3390/ijms26167745 (PMC12386934; doi:10.3390/ijms26167745)
Supplement: Supplementary file 1 [file ijms-26-07745-s001.zip › ijms-3820659-supplementary.pdf]

Article

Characteristics and Potential of KSL, KSL-W, and Dadapin-1 Antimicrobial Peptides for Preventing Infections of Orthopedic Prosthetic Devices: Identifying the Most Robust Candidate

Davide Campoccia [1,*], Andrea De Donno [1,†], Giulia Bottau [1,†], Gloria Bua [1], Stefano Ravaioli [1], Eleonora Capponi [1], Giovanna Sotgiu [2], Francesco Pegreffi [3,4], Silvia Costantini [5,§] and Carla Renata Arciola [5,6,*]

1 Laboratorio di Patologia delle Infezioni Associate all'Impianto, IRCCS Istituto Ortopedico Rizzoli, Via di Barbiano 1/10, 40136 Bologna, Italy; giulia.bottau@ior.it (Giulia Bottau); andreadedonno@ior.it (A.D.D.); gloria.bua@ior.it (Gloria Bua); stefano.ravaioli@ior.it (S.R.); eleonora.capponi@ior.it (E.C.)

2 Institute for Organic Synthesis and Photoreactivity (ISOF), National Research Council, Via Gobetti 101, 40129 Bologna, Italy; giovanna.sotgiu@isof.cnr.it

3 Osteoncology, Bone and Soft Tissue Sarcomas and Innovative Therapies Unit, IRCCS Istituto Ortopedico Rizzoli, Via di Barbiano 1/10, 40136 Bologna, Italy; chiara.bellotti@ior.it

4 Laboratory of Immunorheumatology and Tissue Regeneration, Laboratory of Pathology of Implant Infections, IRCCS Istituto Ortopedico Rizzoli, Via di Barbiano 1/10, 40136 Bologna, Italy; silvia.costantini4@studio.unibo.it

5 Department of Medical and Surgical Sciences (DIMEC), University of Bologna, Via San Giacomo 14, 40126 Bologna, Italy

* Correspondence: davide.campoccia@ior.it (D.C.); carlarenata.arciola@ior.it (C.R.A.)

† These authors contributed equally to the accomplishment of the experimental investigations.

§ Contributed as a medical resident at the Postgraduate School of Clinical Pathology and Clinical Biochemistry, University of Bologna.

Table S1. Ribotyping analysis

Strain	Ribopattern	Ribogroup
SA 01	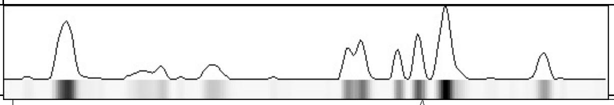	218-330-S-5
SA 02	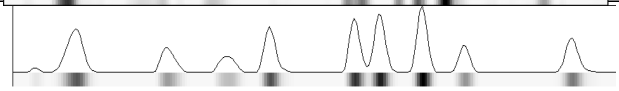	218-97-S-6
SE 01	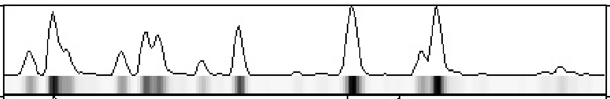	218-53-S-8
SE 02	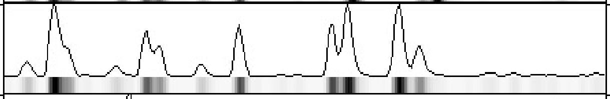	218-63-S-4
EF 01	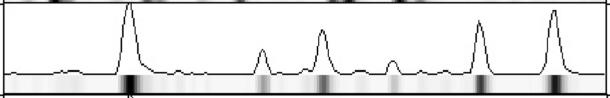	218-330-S-4
EF 02	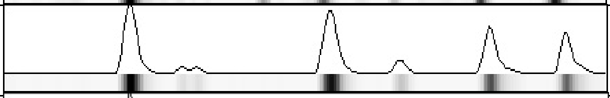	218-115-S-1
EC	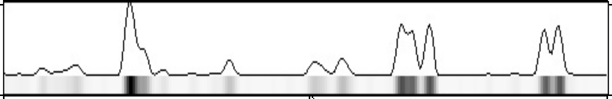	218-330-S-8
PA 01	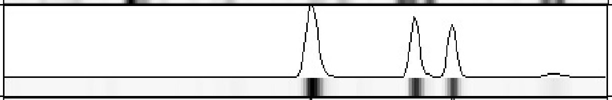	218-246-S-6
PA 02	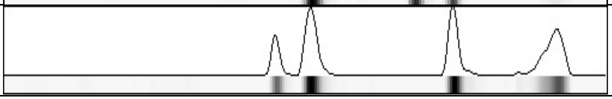	218-299-S-3

Legend: description of strain ribopatterns and ribogroups. The ribopattern shows specific profiles. On the left and centre side the peaks show the highly conserved regions, characteristic for identifying the genus and species.

Table S2. Antibiotic resistance of staphylococcal strains.

SA Strain	Antibiotic resistance										
	AMX	OXA	PEN	BPR	CIP	GEN	TOB	VAN	CLI	TGC	SXT
SA 01	S	S	S	S	I	S	S	S	S	S	S
SA 02	R	R	R	R	R	R	R	S	R	S	R
SE 02	S	S	S	-	I	S	S	S	S	S	S
SE 01	-	R	R	-	I	S	S	S	S	S	S

Legend: AMX, Amoxicillin; OXA, Oxacillin; PEN, Penicillin G; BPR, Ceftobiprole; CIP, Ciprofloxacin; GEN, Gentamicin; TOB, Tobramycin; VAN, Vancomycin; CLI, Clindamycin; TGC, Tigecycline; SXT, Trimethoprim-Sulfamethoxazole; S, antibiotic-sensitive; R, antibiotic-resistant; I, intermediate.

Table S3. Antibiotic resistance of *EF* strains.

EF Strain	Antibiotic resistance					
	AMX	PIP	IPM	CIP	VAN	TGC
EF 01	S	S	I	S	S	S
EF 02	S	S	I	S	S	S

Legend: AMX, Amoxicillin; PIP, Piperacillin; IPM, Imipenem; CIP, Ciprofloxacin; VAN, Vancomycin; TGC, Tigecycline; S, antibiotic-sensitive; I, intermediate.

Table S4. Antibiotic resistance of Gram- strains.

Gram- strain	Antibiotic resistance									
	AMX	PIP	CAZ	BPR	IPM	CIP	GEN	TOB	TGC	SXT
EC	R	R	R	R	S	R	R	R	S	R
PA 01	-	I	I	-	I	I	S	S	-	-
PA 02	-	I	I	-	I	I	S	S	-	-

Legend: AMX, Amoxicillin; PIP, Piperacillin; CAZ, Ceftazidime; BPR, Ceftobiprole; IPM, Imipenem; CIP, Ciprofloxacin; GEN, Gentamicin; TOB, Tobramycin; TGC, Tigecycline; SXT, Trimethoprim*-Sulfamethoxazole; PEN, Penicillin G; S, antibiotic-sensitive; R, antibiotic-resistant; I, intermediate.

Table S5. Antibacterial activity of AMPs in undiluted MHB II medium.

Strain	100% MHB II					
	MIC [µM]			MBC [µM]		
	KSL	KSL-W	Dadapin-1	KSL	KSL-W	Dadapin-1
SA 00	25.0	11.9	198.9 (>198.9)	25.0-50.0	11.9	>198.9 (>198.9)
SA 01	50.0	23.9	(>198.9)	100	23.9-47.8	(>198.9)
SA 02	25.0	11.9	99.4->198.9	25.0-50.0	11.9	>198.9
SE 01	1.56-3.13	2.99	>198.9 (99.4)	3.13	2.99	>198.9 (>198.9)
SE 02	0.78-1.56	0.37-0.75	>198.9	3.13	1.49	>198.9 (>198.9)
EF 01	100-200	11.9-23.9	>198.9	200	11.9-23.9	>198.9
EF 02	ND $^{OD/L}$	11.9-23.9	>198.9	>400	23.9-47.8	>198.9
EC	6.25	2.99	>198.9 (>198.9)	6.25-12.5	2.99	>198.9 (>198.9)
PA 01	ND ^{L} 50.0-100 ^{OD}	ND ^{L} 23.9 ^{OD}	>198.9 (>198.9)	100-200	23.9-47.8	>198.9 (>198.9)
PA 02	ND ^{L} 50.0-100^{OD}	ND ^{L} 23.9 ^{OD}	>198.9	50.0-200	23.9	>198.9

When not diversely specified, MIC values refer to results obtained by luminescence. For Dadapin-1, values between brackets indicate earlier published results [21]. For the earlier published data of Dadapin-1, MBC values were tested by plating 100 µl of culture medium instead of 10 µl. Legend: L, value determined by ATP luminescence assay; OD, value determined by optical density reading; ND, MIC could not be determined; green cells: lowest MIC and MBC values observed for a given bacterial strain across the different peptides; grey cells: same MIC or MBC values (or overlapping ranges of values); red cells: highest MIC and MBC observed.

Table S6. Heatmap of MIC and MBC values obtained in undiluted medium

Strain	100% MHB II (µM)					
	MIC			MBC		
	**KSL**	**KSL-W**	**Dadapin-1**	**KSL**	**KSL-W**	**Dadapin-1**
SA 00	25.0	11.9	198.9<br>>198.9	25.0<br>50.0	11.9	>198.9
SA 01	50.0	23.9	>198.9	100	23.9<br>47.8	>198.9
SA 02	25.0	11.9	99.4<br>>198.9	25.0<br>50.0	11.9	>198.9
SE 01	1.56<br>3.13	2.99	99.4<br>198.9	3.13	2.99	>198.9
SE 02	0.78<br>1.56	0.37<br>0.75	99.4<br>>198.9	3.13	1.49	>198.9
EF 01	100<br>200	11.9<br>23.9	>198.9	200	11.9<br>23.9	>198.9
EF 02	ND OD/L	11.9<br>23.9	>198.9	>400	23.9<br>47.8	>198.9
EC	6.25	2.99	>198.9	6.25<br>12.5	2.99	>198.9
PA 01	50<br>100	23.9	>198.9	100<br>200	23.9<br>47.8	>198.9
PA 02	50<br>100	23.9	>198.9	50.0<br>200	23.9	>198.9

Legend: in case of a range of values, the lower value has been reported in the upper half of the cell and the higher value in the lower half. For Dadapin-1 the range considers new and old values.

Table S7. Detailed view of all MIC, MBIC and MBC measurements of KSL in undiluted medium

KSL - 100% MHB II		MIC µg/mL (µM)			MBIC µg/mL (µM)			MBC µg/mL (µM)		
		N. Exp.	Values	Range	N. Exp.	Values	Range	N. Exp.	Values	Range
SA	SA 00	**3**	**31.25-31.25-31.25 (25.0-25.0-25.0)**	**31.25 (25.0)**	**3**	**15.63-15.63-15.63 (12.5-12.5-12.5)**	**15.63 (12.5)**	**3**	**31.25-62.5-62.5 (25.0-50.0-50.0)**	**31.25-62.5 (25.0-50.0)**
	SA 01	**3**	**62.5-62.5-62.5 (50.0-50.0-50.0)**	**62.5 (50.0)**	**3**	**62.5-31.25-62.5 (50.0-25.0-50.0)**	**31.25-62.5 (25.0-50.0)**	**3**	**125-125-125 (100-100-100)**	**125 (100)**
	SA 02	**3**	**31.25-31.25-31.25 (25.0-25.0-25.0)**	**31.25 (25.0)**	**3**	**31.25-31.25-31.25 (25.0-25.0-25.0)**	**31.25 (25.0)**	**3**	**62.5-31.25-62.5 (50.0-25.0-50.0)**	**31.25-62.5 (25.0-50.0)**
SE	SE 01	**3**	**3.91-3.91-1.95 (3.13-3.13-1.56)**	**1.95-3.91 (1.56-3.13)**	**3**	**3.91-3.91-3.91 (3.13-3.13-3.13)**	**3.91 (3.13)**	**3**	**3.91-3.91-3.91 (3.13-3.13-3.13)**	**3.91 (3.13)**
	SE 02	**3**	**0.98-1.95-1.95 (0.78-1.56-1.56)**	**0.98-1.95 (0.78-1.56)**	**3**	**1.95-1.95-1.95 (1.56-1.56-1.56)**	**1.95 (1.56)**	**3**	**3.91-3.91-3.91 (3.13-3.13-3.13)**	**3.91 (3.13)**
EF	EF 01	**3**	**125-125-250 (100-100-200)**	**125-250 (100-200)**	**3**	**125-125-125 (100-100-100)**	**125 (100)**	**3**	**250-250-250 (200-200-200)**	**250 (200)**
	EF 02	**3**	**250-N.D.-N.D. ^{OD} N.D.-N.D.-N.D. ^{L}**	**N.D. $^{OD/L}$**	**3**	**250-250-250 (200-200-200)**	**250 (200)**	**3**	**>250->500->500 (>400->400->400)**	**>500 (>400)**
EC	EC	**3**	**7.81-7.81-7.81 (6.25-6.25-6.25)**	**7.81 (6.25)**	**3**	**7.81-7.81-7.81 (6.25-6.25-6.25)**	**7.81 (6.25)**	**3**	**7.81-7.81-15.63 (6.25-6.25-12.5)**	**7.81-15.63 (6.25-12.5)**
PS	PA 01	**3**	**125-62.5-125 (100-50.0-100) ^{OD} N.D.-N.D.-N.D. ^{L}**	**62.5-125 (50.0-100) ^{OD} N.D. ^{L}**	**3**	**125-62.5-125 (100-50.0-100)**	**62.5-125 (50.0-100)**	**3**	**250-125-250 (200-100-200)**	**125-250 (100-250)**
	PA 02	**3**	**62.5-125-62.5 (50.0-100-50.0) ^{OD} N.D.-N.D.-N.D. ^{L}**	**62.5-125 (50.0-100) ^{OD} N.D. ^{L}**	**3**	**62.5-125-62.5 (50.0-100-50.0)**	**62.5-125 (50.0-100)**	**3**	**62.5-250-125 (50.0-200-100)**	**62.5-250 (50.0-200)**

Legend: N. Exp., number of experiments performed; OD, measurements by OD reading; L, measurements by luminescence reading.

Table S8. Detailed view of all MIC, MBIC and MBC measurements of KSL-W in undiluted medium

KSL - 100% MHB II		MIC µg/mL (µM)			MBIC µg/mL (µM)			MBC µg/mL (µM)		
		**N. Exp.**	**Values**	**Range**	**N. Exp.**	**Values**	**Range**	**N. Exp.**	**Values**	**Range**
SA	SA 00	**3**	**15.63-15.63-15.63<br>(11.9-11.9-11.9)**	**15.63<br>(11.9) [L]**	**3**	**7.81-7.81-7.81<br>(5.97-5.97-5.97) [L]**	**7.81<br>(5.97)**	**3**	**15.63-15.63-15.63<br>(11.9-11.9-11.9)**	**15.63<br>(11.9)**
	SA 01	**3**	**31.25-31.25-31.25<br>(23.9-23.9-23.9)**	**31.25<br>(23.9) [L]**	**3**	**31.25-31.25-31.25<br>(23.9-23.9-23.9) [L]**	**31.25<br>(23.9)**	**3**	**31.25-62.5-62.5<br>(23.9-47.8-47.8)**	**31.25-62.5<br>(23.9-47.8)**
	SA 02	**3**	**15.63-15.63-15.63<br>(11.9-11.9-11.9)**	**15.63<br>(11.9) [L]**	**3**	**15.63-15.63-15.63<br>(11.9-11.9-11.9)**	**15.63<br>(11.9)**	**3**	**15.63-15.63-15.63<br>(11.9-11.9-11.9)**	**15.63<br>(11.9)**
SE	SE 01	**3**	**3.91-3.91-3.91<br>(2.99-2.99-2.99)**	**3.91<br>(2.99) [L]**	**3**	**3.91-3.91-1.95<br>(2.99-2.99-1.49)**	**1.95-3.91<br>(1.49-2.99)**	**3**	**3.91-3.91-3.91<br>(2.99-2.99-2.99)**	**3.91<br>(2.99)**
	SE 02	**4**	**0.49-0.98-0.98-0.49<br>(0.37-0.75-0.75-0.37)**	**0.49-0.98<br>(0.37-0.75)**	**4**	**0.98-1.95-0.49-0.49<br>(0.75-1.49-0.37-0.37)**	**0.49-1.95<br>(0.37-1.49)**	**3**	**1.95-1.95-1.95<br>(1.49-1.49-1.49)**	**1.95<br>(1.49)**
EF	EF 01	**3**	**15.63-15.63-31.25<br>(11.9-11.9-23.9)**	**15.63-31.25<br>(11.9-23.9)**	**3**	**15.63-15.63-31.25<br>(11.9-11.9-23.9)**	**15.63-31.25<br>(11.9-23.9)**	**3**	**31.25-15.63-31.25<br>(23.9-11.9-23.9)**	**15.63-31.25<br>(11.9-23.9)**
	EF 02	**3**	**15.6-31.25-31.25<br>(11.9-23.9-23.9)**	**15.63-31.25<br>(11.9-23.9)**	**4**	**15.63-15.63-15.63-31.25<br>(11.9-11.9-11.9-23.9)**	**15.63-31.25<br>(11.9-23.9)**	**4**	**62.5-31.25-31.25-31.25<br>(47.8-23.9-23.9-23.9)**	**31.25-62.5<br>(23.9-47.8)**
EC	EC	**3**	**3.91-3.91-3.91<br>(2.99-2.99-2.99)**	**3.91<br>(2.99)**	**3**	**3.91-3.91-3.91<br>(2.99-2.99-2.99)**	**3.91<br>(2.99)**	**3**	**3.91-3.91-3.91<br>(2.99-2.99-2.99)**	**3.91<br>(2.99)**
PA	PA 01	**3**	**31.25-31.25-31.25<br>(23.9-23.9-23.9) [OD] N.D.-N.D.-N.D. [L]**	**31.25<br>(23.9) [OD] N.D. [L]**	**3**	**31.25-31.25-31.25<br>(23.9-23.9-23.9)**	**31.25<br>(23.9)**	**3**	**62.5-31.25-31.25<br>(47.8-23.9-23.9)**	**31.25-62.5<br>(23.9-47.8)**
	PA 02	**3**	**31.25-31.25-31.25<br>(23.9-23.9-23.9) [OD] N.D.-N.D.-N.D. [L]**	**31.25<br>(23.9) [OD] N.D. [L]**	**3**	**31.25-31.25-31.25<br>(23.9-23.9-23.9)**	**31.25<br>(23.9)**	**3**	**31.25-31.25-31.25<br>(23.9-23.9-23.9)**	**31.25<br>(23.9)**

Legend: N. Exp., number of experiments performed; OD, measurements by OD reading; L, measurements by luminescence reading.

Table S9. MIC, MBIC and MBC measurements of Dadapin-1 in undiluted medium performed in the present study either to test new strains or for re-testing past strains

Dadapin-1 - 100% MHB II		MIC µg/mL (µM)			MBIC µg/mL (µM)			MBC µg/mL (µM)		
		**N. Exp.**	**Values**	**Range**	**N. Exp.**	**Values**	**Range**	**N. Exp.**	**Values**	**Range**
SA	SA 00	**3**	**500-500-500 (198.9-198.9-198.9)**	**500 (198.9)**	**3**	**250-500-250 (99.4-198.9-99.4)**	**250-500 (99.4-198.9)**	**3**	**>500->500->500 (>198.9->198.9->198.9)**	**>500 (>198.9)**
	SA 02	**3**	**250->500->500 (99.4->198.9->198.9)**	**250->500 (99.4->198.9)**	**3**	**500-500-500 (198.9-198.9-198.9)**	**500 (198.9)**	**3**	**>500->500->500 (>198.9->198.9->198.9)**	**>500 (>198.9)**
SE	SE 01	**2**	**500-500 (198.9-198.9)**	**500 (198.9)**	**2**	**500-500 (198.9-198.9)**	**500 (198.9)**	**2**	**>500->500 (>198.9->198.9)**	**>500 (>198.9)**
	SE 02	**2**	**>500->500 (>198.9->198.9)**	**>500 (>198.9)**	**2**	**>500->500 (>198.9->198.9)**	**>500 (>198.9)**	**2**	**>500->500 (>198.9->198.9)**	**>500 (>198.9)**
EF	EF 01	**2**	**>500->500 (>198.9->198.9)**	**>500 (>198.9)**	**2**	**>500->500 (>198.9->198.9)**	**>500 (>198.9)**	**2**	**>500->500 (>198.9->198.9)**	**>500 (>198.9)**
	EF 02	**2**	**>500->500 (>198.9->198.9)**	**>500 (>198.9)**	**2**	**>500->500 (>198.9->198.9)**	**>500 (>198.9)**	**2**	**>500->500 (>198.9->198.9)**	**>500 (>198.9)**
EC	EC	**1**	**>500 (>198.9)**	**>500 (>198.9)**	**1**	**>500 (>198.9)**	**>500 (>198.9)**	**1**	**>500 (>198.9)**	**>500 (>198.9)**
PA	PA 01	**1**	**>500 (>198.9)**	**>500 (>198.9)**	**1**	**>500 (>198.9)**	**>500 (>198.9)**	**1**	**>500 (>198.9)**	**>500 (>198.9)**
	PA 02	**2**	**>500->500 (>198.9->198.9)**	**>500 (>198.9)**	**2**	**>500->500 (>198.9->198.9)**	**>500 (>198.9)**	**2**	**>500->500 (>198.9->198.9)**	**>500 (>198.9)**

Legend: N. Exp., number of experiments performed.

Table S10. Two-way ANOVA analysis of Log_2 (MIC) of KSL and KSL-W in undiluted (100%) and diluted (20%) MHB II.

Table Analyzed	Log_2 MIC				
Two-way ANOVA	Ordinary				
Alpha	0.05				
Source of Variation	% of total variation	P value	P value summary	Significant?	
Interaction	8.695	<0.0001	****	Yes	
Row Factor	49.66	<0.0001	****	Yes	
Column Factor	39.92	<0.0001	****	Yes	
ANOVA table	SS (Type III)	DF	MS	F (DFn, DFd)	P value
Interaction	46.17	24	1.924	F (24, 75) = 12.47	P<0.0001
Row Factor	263.7	8	32.96	F (8, 75) = 213.6	P<0.0001
Column Factor	212	3	70.66	F (3, 75) = 457.9	P<0.0001
Residual	11.57	75	0.1543		
Data summary					
Number of columns (Column Factor)	4				
Number of rows (Row Factor)	9				
Number of values	111				

Table S11. Tukey's multiple comparisons test of Log_2 (MIC) of KSL and KSL-W in undiluted (100%) and diluted (20%) MHB II.

Within each row, compare columns (simple effects within rows)					
Number of families	9				
Number of comparisons per family	6				
Alpha	0.05				
Tukey's multiple comparisons test	Predicted (LS) mean diff.	95.00% CI of diff.	Below threshold?	Summary	Adjusted P Value
SA 00					
Log2 KSL 100% vs. Log2 KSL-W 100%	1	0.1572 to 1.843	Yes	*	0.0135
Log2 KSL 100% vs. Log2 KSL 20%	4.01	3.167 to 4.853	Yes	****	<0.0001
Log2 KSL 100% vs. Log2 KSL-W 20%	3.673	2.831 to 4.516	Yes	****	<0.0001
Log2 KSL-W 100% vs. Log2 KSL 20%	3.01	2.167 to 3.853	Yes	****	<0.0001
Log2 KSL-W 100% vs. Log2 KSL-W 20%	2.673	1.831 to 3.516	Yes	****	<0.0001
Log2 KSL 20% vs. Log2 KSL-W 20%	-0.3367	-1.179 to 0.5061	No	ns	0.7209
SA 01					
Log2 KSL 100% vs. Log2 KSL-W 100%	1	0.1572 to 1.843	Yes	*	0.0135
Log2 KSL 100% vs. Log2 KSL 20%	5.01	4.167 to 5.853	Yes	****	<0.0001
Log2 KSL 100% vs. Log2 KSL-W 20%	4.673	3.831 to 5.516	Yes	****	<0.0001
Log2 KSL-W 100% vs. Log2 KSL 20%	4.01	3.167 to 4.853	Yes	****	<0.0001
Log2 KSL-W 100% vs. Log2 KSL-W 20%	3.673	2.831 to 4.516	Yes	****	<0.0001
Log2 KSL 20% vs. Log2 KSL-W 20%	-0.3367	-1.179 to 0.5061	No	ns	0.7209
SA 02					

Log2 KSL 100% vs. Log2 KSL-W 100%	1 0.1572 to 1.843	Yes	*	0.0135
Log2 KSL 100% vs. Log2 KSL 20%	3.673 2.831 to 4.516	Yes	****	<0.0001
Log2 KSL 100% vs. Log2 KSL-W 20%	3 2.157 to 3.843	Yes	****	<0.0001
Log2 KSL-W 100% vs. Log2 KSL 20%	2.673 1.831 to 3.516	Yes	****	<0.0001
Log2 KSL-W 100% vs. Log2 KSL-W 20%	2 1.157 to 2.843	Yes	****	<0.0001
Log2 KSL 20% vs. Log2 KSL-W 20%	-0.6733 -1.516 to 0.1695	No	ns	0.1628
SE 01				
Log2 KSL 100% vs. Log2 KSL-W 100%	-0.3367 -1.179 to 0.5061	No	ns	0.7209
Log2 KSL 100% vs. Log2 KSL 20%	1.997 1.154 to 2.839	Yes	****	<0.0001
Log2 KSL 100% vs. Log2 KSL-W 20%	1.333 0.4905 to 2.176	Yes	***	0.0005
Log2 KSL-W 100% vs. Log2 KSL 20%	2.333 1.491 to 3.176	Yes	****	<0.0001
Log2 KSL-W 100% vs. Log2 KSL-W 20%	1.67 0.8272 to 2.513	Yes	****	<0.0001
Log2 KSL 20% vs. Log2 KSL-W 20%	-0.6633 -1.506 to 0.1795	No	ns	0.1731
SE 02				
Log2 KSL 100% vs. Log2 KSL-W 100%	1.16 0.3716 to 1.948	Yes	**	0.0013
Log2 KSL 100% vs. Log2 KSL 20%	1.66 0.8172 to 2.503	Yes	****	<0.0001
Log2 KSL 100% vs. Log2 KSL-W 20%	1.327 0.4839 to 2.169	Yes	***	0.0005
Log2 KSL-W 100% vs. Log2 KSL 20%	0.5 -0.2884 to 1.288	No	ns	0.3485
Log2 KSL-W 100% vs. Log2 KSL-W 20%	0.1667 -0.6217 to 0.9550	No	ns	0.9448
Log2 KSL 20% vs. Log2 KSL-W 20%	-0.3333 -1.176 to 0.5095	No	ns	0.727
EF 01				
Log2 KSL 100% vs. Log2 KSL-W 100%	3 2.157 to 3.843	Yes	****	<0.0001
Log2 KSL 100% vs. Log2 KSL 20%	3.333 2.491 to 4.176	Yes	****	<0.0001
Log2 KSL 100% vs. Log2 KSL-W 20%	4 3.157 to 4.843	Yes	****	<0.0001
Log2 KSL-W 100% vs. Log2 KSL 20%	0.3333 -0.5095 to 1.176	No	ns	0.727

Log2 KSL-W 100% vs. Log2 KSL-W 20%	1	0.1572 to 1.843	Yes	*	0.0135
Log2 KSL 20% vs. Log2 KSL-W 20%	0.6667	-0.1761 to 1.509	No	ns	0.1696
EC					
Log2 KSL 100% vs. Log2 KSL-W 100%	1	0.1572 to 1.843	Yes	*	0.0135
Log2 KSL 100% vs. Log2 KSL 20%	2.01	1.167 to 2.853	Yes	****	<0.0001
Log2 KSL 100% vs. Log2 KSL-W 20%	1.337	0.4939 to 2.179	Yes	***	0.0005
Log2 KSL-W 100% vs. Log2 KSL 20%	1.01	0.1672 to 1.853	Yes	*	0.0123
Log2 KSL-W 100% vs. Log2 KSL-W 20%	0.3367	-0.5061 to 1.179	No	ns	0.7209
Log2 KSL 20% vs. Log2 KSL-W 20%	-0.6733	-1.516 to 0.1695	No	ns	0.1628
PA 01					
Log2 KSL 100% vs. Log2 KSL-W 100%	1.667	0.8239 to 2.509	Yes	****	<0.0001
Log2 KSL 100% vs. Log2 KSL 20%	4.667	3.824 to 5.509	Yes	****	<0.0001
Log2 KSL 100% vs. Log2 KSL-W 20%	4.667	3.824 to 5.509	Yes	****	<0.0001
Log2 KSL-W 100% vs. Log2 KSL 20%	3	2.157 to 3.843	Yes	****	<0.0001
Log2 KSL-W 100% vs. Log2 KSL-W 20%	3	2.157 to 3.843	Yes	****	<0.0001
Log2 KSL 20% vs. Log2 KSL-W 20%	0	-0.8428 to 0.8428	No	ns	>0.9999
PA 02					
Log2 KSL 100% vs. Log2 KSL-W 100%	1.333	0.4905 to 2.176	Yes	***	0.0005
Log2 KSL 100% vs. Log2 KSL 20%	4.333	3.491 to 5.176	Yes	****	<0.0001
Log2 KSL 100% vs. Log2 KSL-W 20%	3.733	2.980 to 4.487	Yes	****	<0.0001
Log2 KSL-W 100% vs. Log2 KSL 20%	3	2.157 to 3.843	Yes	****	<0.0001
Log2 KSL-W 100% vs. Log2 KSL-W 20%	2.4	1.646 to 3.154	Yes	****	<0.0001
Log2 KSL 20% vs. Log2 KSL-W 20%	-0.6	-1.354 to 0.1538	No	ns	0.1653

Test details	Predicted (LS) mean 1	Predicted (LS) mean 2	Predicted (LS) mean diff.	SE of diff.	N1	N2	q	DF
SA 00								
Log2 KSL 100% vs. Log2 KSL-W 100%	4.97	3.97	1	0.3207	3	3	4.409	75
Log2 KSL 100% vs. Log2 KSL 20%	4.97	0.96	4.01	0.3207	3	3	17.68	75
Log2 KSL 100% vs. Log2 KSL-W 20%	4.97	1.297	3.673	0.3207	3	3	16.2	75
Log2 KSL-W 100% vs. Log2 KSL 20%	3.97	0.96	3.01	0.3207	3	3	13.27	75
Log2 KSL-W 100% vs. Log2 KSL-W 20%	3.97	1.297	2.673	0.3207	3	3	11.79	75
Log2 KSL 20% vs. Log2 KSL-W 20%	0.96	1.297	-0.3367	0.3207	3	3	1.484	75
SA 01								
Log2 KSL 100% vs. Log2 KSL-W 100%	5.97	4.97	1	0.3207	3	3	4.409	75
Log2 KSL 100% vs. Log2 KSL 20%	5.97	0.96	5.01	0.3207	3	3	22.09	75
Log2 KSL 100% vs. Log2 KSL-W 20%	5.97	1.297	4.673	0.3207	3	3	20.61	75
Log2 KSL-W 100% vs. Log2 KSL 20%	4.97	0.96	4.01	0.3207	3	3	17.68	75
Log2 KSL-W 100% vs. Log2 KSL-W 20%	4.97	1.297	3.673	0.3207	3	3	16.2	75
Log2 KSL 20% vs. Log2 KSL-W 20%	0.96	1.297	-0.3367	0.3207	3	3	1.484	75
SA 02								
Log2 KSL 100% vs. Log2 KSL-W 100%	4.97	3.97	1	0.3207	3	3	4.409	75
Log2 KSL 100% vs. Log2 KSL 20%	4.97	1.297	3.673	0.3207	3	3	16.2	75
Log2 KSL 100% vs. Log2 KSL-W 20%	4.97	1.97	3	0.3207	3	3	13.23	75
Log2 KSL-W 100% vs. Log2 KSL 20%	3.97	1.297	2.673	0.3207	3	3	11.79	75
Log2 KSL-W 100% vs. Log2 KSL-W 20%	3.97	1.97	2	0.3207	3	3	8.818	75
Log2 KSL 20% vs. Log2 KSL-W 20%	1.297	1.97	-0.6733	0.3207	3	3	2.969	75
SE 01								

Log2 KSL 100% vs. Log2 KSL-W 100%	1.633	1.97	-0.3367	0.3207	3	3	1.484	75
Log2 KSL 100% vs. Log2 KSL 20%	1.633	-0.3633	1.997	0.3207	3	3	8.804	75
Log2 KSL 100% vs. Log2 KSL-W 20%	1.633	0.3	1.333	0.3207	3	3	5.879	75
Log2 KSL-W 100% vs. Log2 KSL 20%	1.97	-0.3633	2.333	0.3207	3	3	10.29	75
Log2 KSL-W 100% vs. Log2 KSL-W 20%	1.97	0.3	1.67	0.3207	3	3	7.363	75
Log2 KSL 20% vs. Log2 KSL-W 20%	-0.3633	0.3	-0.6633	0.3207	3	3	2.925	75
SE 02								
Log2 KSL 100% vs. Log2 KSL-W 100%	0.63	-0.53	1.16	0.3	3	4	5.468	75
Log2 KSL 100% vs. Log2 KSL 20%	0.63	-1.03	1.66	0.3207	3	3	7.319	75
Log2 KSL 100% vs. Log2 KSL-W 20%	0.63	-0.6967	1.327	0.3207	3	3	5.849	75
Log2 KSL-W 100% vs. Log2 KSL 20%	-0.53	-1.03	0.5	0.3	4	3	2.357	75
Log2 KSL-W 100% vs. Log2 KSL-W 20%	-0.53	-0.6967	0.1667	0.3	4	3	0.7856	75
Log2 KSL 20% vs. Log2 KSL-W 20%	-1.03	-0.6967	-0.3333	0.3207	3	3	1.47	75
EF 01								
Log2 KSL 100% vs. Log2 KSL-W 100%	7.303	4.303	3	0.3207	3	3	13.23	75
Log2 KSL 100% vs. Log2 KSL 20%	7.303	3.97	3.333	0.3207	3	3	14.7	75
Log2 KSL 100% vs. Log2 KSL-W 20%	7.303	3.303	4	0.3207	3	3	17.64	75
Log2 KSL-W 100% vs. Log2 KSL 20%	4.303	3.97	0.3333	0.3207	3	3	1.47	75
Log2 KSL-W 100% vs. Log2 KSL-W 20%	4.303	3.303	1	0.3207	3	3	4.409	75
Log2 KSL 20% vs. Log2 KSL-W 20%	3.97	3.303	0.6667	0.3207	3	3	2.939	75
EC								
Log2 KSL 100% vs. Log2 KSL-W 100%	2.97	1.97	1	0.3207	3	3	4.409	75
Log2 KSL 100% vs. Log2 KSL 20%	2.97	0.96	2.01	0.3207	3	3	8.862	75
Log2 KSL 100% vs. Log2 KSL-W 20%	2.97	1.633	1.337	0.3207	3	3	5.894	75
Log2 KSL-W 100% vs. Log2 KSL 20%	1.97	0.96	1.01	0.3207	3	3	4.453	75

Log2 KSL-W 100% vs. Log2 KSL-W 20%	1.97	1.633	0.3367	0.3207	3	3	1.484	75
Log2 KSL 20% vs. Log2 KSL-W 20%	0.96	1.633	-0.6733	0.3207	3	3	2.969	75
PA 01								
Log2 KSL 100% vs. Log2 KSL-W 100%	6.637	4.97	1.667	0.3207	3	3	7.349	75
Log2 KSL 100% vs. Log2 KSL 20%	6.637	1.97	4.667	0.3207	3	3	20.58	75
Log2 KSL 100% vs. Log2 KSL-W 20%	6.637	1.97	4.667	0.3207	3	3	20.58	75
Log2 KSL-W 100% vs. Log2 KSL 20%	4.97	1.97	3	0.3207	3	3	13.23	75
Log2 KSL-W 100% vs. Log2 KSL-W 20%	4.97	1.97	3	0.3207	3	3	13.23	75
Log2 KSL 20% vs. Log2 KSL-W 20%	1.97	1.97	0	0.3207	3	3	0	75
PA 02								
Log2 KSL 100% vs. Log2 KSL-W 100%	6.303	4.97	1.333	0.3207	3	3	5.879	75
Log2 KSL 100% vs. Log2 KSL 20%	6.303	1.97	4.333	0.3207	3	3	19.11	75
Log2 KSL 100% vs. Log2 KSL-W 20%	6.303	2.57	3.733	0.2869	3	5	18.4	75
Log2 KSL-W 100% vs. Log2 KSL 20%	4.97	1.97	3	0.3207	3	3	13.23	75
Log2 KSL-W 100% vs. Log2 KSL-W 20%	4.97	2.57	2.4	0.2869	3	5	11.83	75
Log2 KSL 20% vs. Log2 KSL-W 20%	1.97	2.57	-0.6	0.2869	3	5	2.958	75

Table S12. Two-way ANOVA analysis of Log_2 (MBC) of KSL and KSL-W in undiluted (100%) and diluted (20%) MHB II.

Table Analyzed	Log MBC				
Two-way ANOVA	Ordinary				
Alpha	0.05				
Source of Variation	% of total variation	P value	P value summary	Significant?	
Interaction	11.51	<0.0001	****	Yes	
Row Factor	38.81	<0.0001	****	Yes	
Column Factor	48.2	<0.0001	****	Yes	
ANOVA table	SS (Type III)	DF	MS	F (DFn, DFd)	P value
Interaction	58.75	24	2.448	F (24, 75) = 12.87	P<0.0001
Row Factor	198.1	8	24.77	F (8, 75) = 130.2	P<0.0001
Column Factor	246.1	3	82.02	F (3, 75) = 431.2	P<0.0001
Residual	14.27	75	0.1902		
Data summary					
Number of columns (Column Factor)	4				
Number of rows (Row Factor)	9				
Number of values	111				

Table S13. Tukey's multiple comparisons test of Log_2 (MBC) of KSL and KSL-W in undiluted (100%) and diluted (20%) MHB II.

Within each row, compare columns (simple effects within rows)					
Number of families	9				
Number of comparisons per family	6				
Alpha	0.05				
Tukey's multiple comparisons test	Predicted (LS) mean diff.	95.00% CI of diff.	Below threshold?	Summary	Adjusted P Value
SA 00					
Log2 KSL 100% vs. Log2 KSL-W 100%	1.667	0.7309 to 2.602	Yes	****	<0.0001
Log2 KSL 100% vs. Log2 KSL 20%	4.34	3.404 to 5.276	Yes	****	<0.0001
Log2 KSL 100% vs. Log2 KSL-W 20%	3.667	2.731 to 4.602	Yes	****	<0.0001
Log2 KSL-W 100% vs. Log2 KSL 20%	2.673	1.738 to 3.609	Yes	****	<0.0001
Log2 KSL-W 100% vs. Log2 KSL-W 20%	2	1.064 to 2.936	Yes	****	<0.0001
Log2 KSL 20% vs. Log2 KSL-W 20%	-0.6733	-1.609 to 0.2624	No	ns	0.2407
SA 01					
Log2 KSL 100% vs. Log2 KSL-W 100%	1.333	0.3976 to 2.269	Yes	**	0.002
Log2 KSL 100% vs. Log2 KSL 20%	5.673	4.738 to 6.609	Yes	****	<0.0001
Log2 KSL 100% vs. Log2 KSL-W 20%	5	4.064 to 5.936	Yes	****	<0.0001
Log2 KSL-W 100% vs. Log2 KSL 20%	4.34	3.404 to 5.276	Yes	****	<0.0001
Log2 KSL-W 100% vs. Log2 KSL-W 20%	3.667	2.731 to 4.602	Yes	****	<0.0001
Log2 KSL 20% vs. Log2 KSL-W 20%	-0.6733	-1.609 to 0.2624	No	ns	0.2407
SA 02					
Log2 KSL 100% vs. Log2 KSL-W 100%	1.667	0.7309 to 2.602	Yes	****	<0.0001

Log2 KSL 100% vs. Log2 KSL 20%	4.003	3.068 to 4.939	Yes	****	<0.0001
Log2 KSL 100% vs. Log2 KSL-W 20%	3.667	2.731 to 4.602	Yes	****	<0.0001
Log2 KSL-W 100% vs. Log2 KSL 20%	2.337	1.401 to 3.272	Yes	****	<0.0001
Log2 KSL-W 100% vs. Log2 KSL-W 20%	2	1.064 to 2.936	Yes	****	<0.0001
Log2 KSL 20% vs. Log2 KSL-W 20%	-0.3367	-1.272 to 0.5991	No	ns	0.7805
SE 01					
Log2 KSL 100% vs. Log2 KSL-W 100%	1,110e-015	-0.9357 to 0.9357	No	ns	>0.9999
Log2 KSL 100% vs. Log2 KSL 20%	1.67	0.7343 to 2.606	Yes	****	<0.0001
Log2 KSL 100% vs. Log2 KSL-W 20%	1.01	0.07428 to 1.946	Yes	*	0.0293
Log2 KSL-W 100% vs. Log2 KSL 20%	1.67	0.7343 to 2.606	Yes	****	<0.0001
Log2 KSL-W 100% vs. Log2 KSL-W 20%	1.01	0.07428 to 1.946	Yes	*	0.0293
Log2 KSL 20% vs. Log2 KSL-W 20%	-0.66	-1.596 to 0.2757	No	ns	0.257
SE 02					
Log2 KSL 100% vs. Log2 KSL-W 100%	1.01	0.07428 to 1.946	Yes	*	0.0293
Log2 KSL 100% vs. Log2 KSL 20%	2	1.064 to 2.936	Yes	****	<0.0001
Log2 KSL 100% vs. Log2 KSL-W 20%	1.01	0.07428 to 1.946	Yes	*	0.0293
Log2 KSL-W 100% vs. Log2 KSL 20%	0.99	0.05428 to 1.926	Yes	*	0.034
Log2 KSL-W 100% vs. Log2 KSL-W 20%	0	-0.9357 to 0.9357	No	ns	>0.9999
Log2 KSL 20% vs. Log2 KSL-W 20%	-0.99	-1.926 to -0.05428	Yes	*	0.034
EF 01					
Log2 KSL 100% vs. Log2 KSL-W 100%	3.333	2.398 to 4.269	Yes	****	<0.0001
Log2 KSL 100% vs. Log2 KSL 20%	3.667	2.731 to 4.602	Yes	****	<0.0001
Log2 KSL 100% vs. Log2 KSL-W 20%	4.5	3.625 to 5.375	Yes	****	<0.0001
Log2 KSL-W 100% vs. Log2 KSL 20%	0.3333	-0.6024 to 1.269	No	ns	0.7856
Log2 KSL-W 100% vs. Log2 KSL-W 20%	1.167	0.2914 to 2.042	Yes	**	0.0043
Log2 KSL 20% vs. Log2 KSL-W 20%	0.8333	-0.04196 to 1.709	No	ns	0.0678

EF 02					
Log2 KSL-W 100% vs. Log2 KSL 20%	1.25	0,5135 to 1,987	Yes	***	0.0004
Log2 KSL-W 100% vs. Log2 KSL-W 20%	3.25	2,513 to 3,987	Yes	****	<0,0001
Log2 KSL 20% vs. Log2 KSL-W 20%	2	1,213 to 2,787	Yes	****	<0,0001
EC					
Log2 KSL 100% vs. Log2 KSL-W 100%	1.327	0.3909 to 2.262	Yes	**	0.0021
Log2 KSL 100% vs. Log2 KSL 20%	2	1.064 to 2.936	Yes	****	<0.0001
Log2 KSL 100% vs. Log2 KSL-W 20%	1.663	0.7276 to 2.599	Yes	****	<0.0001
Log2 KSL-W 100% vs. Log2 KSL 20%	0.6733	-0.2624 to 1.609	No	ns	0.2407
Log2 KSL-W 100% vs. Log2 KSL-W 20%	0.3367	-0.5991 to 1.272	No	ns	0.7805
Log2 KSL 20% vs. Log2 KSL-W 20%	-0.3367	-1.272 to 0.5991	No	ns	0.7805
PA 01					
Log2 KSL 100% vs. Log2 KSL-W 100%	2.333	1.398 to 3.269	Yes	****	<0.0001
Log2 KSL 100% vs. Log2 KSL 20%	5.667	4.731 to 6.602	Yes	****	<0.0001
Log2 KSL 100% vs. Log2 KSL-W 20%	5.333	4.398 to 6.269	Yes	****	<0.0001
Log2 KSL-W 100% vs. Log2 KSL 20%	3.333	2.398 to 4.269	Yes	****	<0.0001
Log2 KSL-W 100% vs. Log2 KSL-W 20%	3	2.064 to 3.936	Yes	****	<0.0001
Log2 KSL 20% vs. Log2 KSL-W 20%	-0.3333	-1.269 to 0.6024	No	ns	0.7856
PA 02					
Log2 KSL 100% vs. Log2 KSL-W 100%	2	1.064 to 2.936	Yes	****	<0.0001
Log2 KSL 100% vs. Log2 KSL 20%	5	4.064 to 5.936	Yes	****	<0.0001
Log2 KSL 100% vs. Log2 KSL-W 20%	4.4	3.563 to 5.237	Yes	****	<0.0001
Log2 KSL-W 100% vs. Log2 KSL 20%	3	2.064 to 3.936	Yes	****	<0.0001
Log2 KSL-W 100% vs. Log2 KSL-W 20%	2.4	1.563 to 3.237	Yes	****	<0.0001
Log2 KSL 20% vs. Log2 KSL-W 20%	-0.6	-1.437 to 0.2369	No	ns	0.2438

Test details	Predicted (LS) mean 1	Predicted (LS) mean 2	Predicted (LS) mean diff.	SE of diff.	N1	N2	q	DF
SA 00								
Log2 KSL 100% vs. Log2 KSL-W 100%	5.637	3.97	1.667	0.3561	3	3	6.619	75
Log2 KSL 100% vs. Log2 KSL 20%	5.637	1.297	4.34	0.3561	3	3	17.24	75
Log2 KSL 100% vs. Log2 KSL-W 20%	5.637	1.97	3.667	0.3561	3	3	14.56	75
Log2 KSL-W 100% vs. Log2 KSL 20%	3.97	1.297	2.673	0.3561	3	3	10.62	75
Log2 KSL-W 100% vs. Log2 KSL-W 20%	3.97	1.97	2	0.3561	3	3	7.942	75
Log2 KSL 20% vs. Log2 KSL-W 20%	1.297	1.97	-0.6733	0.3561	3	3	2.674	75
SA 01								
Log2 KSL 100% vs. Log2 KSL-W 100%	6.97	5.637	1.333	0.3561	3	3	5.295	75
Log2 KSL 100% vs. Log2 KSL 20%	6.97	1.297	5.673	0.3561	3	3	22.53	75
Log2 KSL 100% vs. Log2 KSL-W 20%	6.97	1.97	5	0.3561	3	3	19.86	75
Log2 KSL-W 100% vs. Log2 KSL 20%	5.637	1.297	4.34	0.3561	3	3	17.24	75
Log2 KSL-W 100% vs. Log2 KSL-W 20%	5.637	1.97	3.667	0.3561	3	3	14.56	75
Log2 KSL 20% vs. Log2 KSL-W 20%	1.297	1.97	-0.6733	0.3561	3	3	2.674	75
SA 02								
Log2 KSL 100% vs. Log2 KSL-W 100%	5.637	3.97	1.667	0.3561	3	3	6.619	75
Log2 KSL 100% vs. Log2 KSL 20%	5.637	1.633	4.003	0.3561	3	3	15.9	75
Log2 KSL 100% vs. Log2 KSL-W 20%	5.637	1.97	3.667	0.3561	3	3	14.56	75
Log2 KSL-W 100% vs. Log2 KSL 20%	3.97	1.633	2.337	0.3561	3	3	9.279	75
Log2 KSL-W 100% vs. Log2 KSL-W 20%	3.97	1.97	2	0.3561	3	3	7.942	75
Log2 KSL 20% vs. Log2 KSL-W 20%	1.633	1.97	-0.3367	0.3561	3	3	1.337	75

SE 01								
Log2 KSL 100% vs. Log2 KSL-W 100%	1.97	1.97	1,110e-015	0.3561	3	3	4,409e-015	75
Log2 KSL 100% vs. Log2 KSL 20%	1.97	0.3	1.67	0.3561	3	3	6.632	75
Log2 KSL 100% vs. Log2 KSL-W 20%	1.97	0.96	1.01	0.3561	3	3	4.011	75
Log2 KSL-W 100% vs. Log2 KSL 20%	1.97	0.3	1.67	0.3561	3	3	6.632	75
Log2 KSL-W 100% vs. Log2 KSL-W 20%	1.97	0.96	1.01	0.3561	3	3	4.011	75
Log2 KSL 20% vs. Log2 KSL-W 20%	0.3	0.96	-0.66	0.3561	3	3	2.621	75
SE 02								
Log2 KSL 100% vs. Log2 KSL-W 100%	1.97	0.96	1.01	0.3561	3	3	4.011	75
Log2 KSL 100% vs. Log2 KSL 20%	1.97	-0.03	2	0.3561	3	3	7.942	75
Log2 KSL 100% vs. Log2 KSL-W 20%	1.97	0.96	1.01	0.3561	3	3	4.011	75
Log2 KSL-W 100% vs. Log2 KSL 20%	0.96	-0.03	0.99	0.3561	3	3	3.931	75
Log2 KSL-W 100% vs. Log2 KSL-W 20%	0.96	0.96	0	0.3561	3	3	0	75
Log2 KSL 20% vs. Log2 KSL-W 20%	-0.03	0.96	-0.99	0.3561	3	3	3.931	75
EF 01								
Log2 KSL 100% vs. Log2 KSL-W 100%	7.97	4.637	3.333	0.3561	3	3	13.24	75
Log2 KSL 100% vs. Log2 KSL 20%	7.97	4.303	3.667	0.3561	3	3	14.56	75
Log2 KSL 100% vs. Log2 KSL-W 20%	7.97	3.47	4.5	0.3331	3	4	19.1	75
Log2 KSL-W 100% vs. Log2 KSL 20%	4.637	4.303	0.3333	0.3561	3	3	1.324	75
Log2 KSL-W 100% vs. Log2 KSL-W 20%	4.637	3.47	1.167	0.3331	3	4	4.953	75
Log2 KSL 20% vs. Log2 KSL-W 20%	4.303	3.47	0.8333	0.3331	3	4	3.538	75
EF 02								
Log2 KSL-W 100% vs. Log2 KSL 20%	5.22	3.97	1.25	0.3069	4	3	5.759	64
Log2 KSL-W 100% vs. Log2 KSL-W 20%	5.22	1.97	3.25	0.3069	4	3	14.97	64
Log2 KSL 20% vs. Log2 KSL-W 20%	3.97	1.97	2	0.3281	3	3	8.619	64

EC								
Log2 KSL 100% vs. Log2 KSL-W 100%	3.297	1.97	1.327	0.3561	3	3	5.268	75
Log2 KSL 100% vs. Log2 KSL 20%	3.297	1.297	2	0.3561	3	3	7.942	75
Log2 KSL 100% vs. Log2 KSL-W 20%	3.297	1.633	1.663	0.3561	3	3	6.605	75
Log2 KSL-W 100% vs. Log2 KSL 20%	1.97	1.297	0.6733	0.3561	3	3	2.674	75
Log2 KSL-W 100% vs. Log2 KSL-W 20%	1.97	1.633	0.3367	0.3561	3	3	1.337	75
Log2 KSL 20% vs. Log2 KSL-W 20%	1.297	1.633	-0.3367	0.3561	3	3	1.337	75
PA 01								
Log2 KSL 100% vs. Log2 KSL-W 100%	7.637	5.303	2.333	0.3561	3	3	9.266	75
Log2 KSL 100% vs. Log2 KSL 20%	7.637	1.97	5.667	0.3561	3	3	22.5	75
Log2 KSL 100% vs. Log2 KSL-W 20%	7.637	2.303	5.333	0.3561	3	3	21.18	75
Log2 KSL-W 100% vs. Log2 KSL 20%	5.303	1.97	3.333	0.3561	3	3	13.24	75
Log2 KSL-W 100% vs. Log2 KSL-W 20%	5.303	2.303	3	0.3561	3	3	11.91	75
Log2 KSL 20% vs. Log2 KSL-W 20%	1.97	2.303	-0.3333	0.3561	3	3	1.324	75
PA 02								
Log2 KSL 100% vs. Log2 KSL-W 100%	6.97	4.97	2	0.3561	3	3	7.942	75
Log2 KSL 100% vs. Log2 KSL 20%	6.97	1.97	5	0.3561	3	3	19.86	75
Log2 KSL 100% vs. Log2 KSL-W 20%	6.97	2.57	4.4	0.3185	3	5	19.54	75
Log2 KSL-W 100% vs. Log2 KSL 20%	4.97	1.97	3	0.3561	3	3	11.91	75
Log2 KSL-W 100% vs. Log2 KSL-W 20%	4.97	2.57	2.4	0.3185	3	5	10.66	75
Log2 KSL 20% vs. Log2 KSL-W 20%	1.97	2.57	-0.6	0.3185	3	5	2.664	75

Table S14. MBC/MIC ratio with 100% MHB II.

Strain	KSL	KSL-W	Dadapin-1
	MBC/MIC	MBC/MIC	MBC/MIC
SA 00	1–2	1	-
SA 01	2	1–2	-
SA 02	1–2	1	-
SE 01	1–2	1	-
SE 02	2–**4**	2–**4**	**-**
EF 01	1–2	1	-
EF 02	-	2	-
EC	1–2	1	-
PA 01	2	1–2	-
PA 02	1–2	1	-

Legend: values greater than two are in bold. n new Dadapin-1 results from the present study.

Table S15. Antibacterial activity of AMPs in diluted MHB II medium.

Strain	20% MHB II					
	MIC [µM]			MBC [µM]		
	KSL	KSL-W	Dadapin-1	KSL	KSL-W	Dadapin-1
SA 00	1.56	1.49-2.99	6.22 (3.11)	1.56-3.13	2.99	(6.22 -12.43)
SA 01	1.56	1.49-2.99	(6.22)	1.56-3.13	2.99	(6.22)
SA 02	1.56-3.13	2.99	6.22	1.56-3.13	2.99	6.22
SE 01	0.39-0.78	0.75-1.49	1.55-3.11 (3.11)	0.78-1.56	1.49	(3.11)
SE 02	0.39	0.37-0.75	(3.11)	0.78	1.49	6.22 (12.43)
EF 01	12.5	5.97-11.9	ND $^{L/ODn}$	12.5-25.0	5.97-11.9	12.43
EF 02	ND $^{L/OD}$	ND $^{L/OD}$	ND $^{L/ODn}$	12.5	2.99	>198.9
EC	1.56 $^{L/OD}$	1.49-2.99 $^{L/OD}$	(12.43)	1.56-3.13	1.49-2.99	12.43-24.9 (12.43)
PA 01	3.13 $^{L/OD}$	2.99 $^{L/OD}$	(12.43)	3.13	2.99-5.97	(12.43)
PA 02	3.13 $^{L/OD}$	2.99-5.97 $^{L/OD}$	12.43	3.13	2.99-5.97	12.43

When not diversely specified MIC values refer to results obtained by luminescence. For Dadapin-1, values between brackets indicate earlier published results [21] and new data are introduced only when differing. MBC values from the past study were tested by plating 100 µl of culture medium instead of 10 µl. Legend: ^{L}, value determined by ATP luminescence assay; ^{OD}, value determined by optical density reading; ND, MIC could not be determined (likely due to the poor growth of the bacterial strain in diluted MHB II); green cells: lowest MIC and MBC values observed for a given bacterial strain across the different peptides; grey cells: same MIC or MBC values (or overlapping ranges of values); red cells: highest MIC and MBC observed.

Table S16. Heatmap of MIC and MBC values obtained in diluted medium.

<table>
<tr><th rowspan="3">Bacterial strain</th><th colspan="6">20% MHB II (μM)</th></tr>
<tr><th colspan="3">MIC</th><th colspan="3">MBC</th></tr>
<tr><th>KSL</th><th>KSL-W</th><th>Dadapin-1</th><th>KSL</th><th>KSL-W</th><th>Dadapin-1</th></tr>
<tr><td rowspan="2">SA 00</td><td rowspan="2">1.56</td><td>1.49</td><td>3.1</td><td>1.56</td><td rowspan="2">2.99</td><td>6.2</td></tr>
<tr><td>2.99</td><td>6.2</td><td>3.13</td><td>12.4</td></tr>
<tr><td rowspan="2">SA 01</td><td rowspan="2">1.56</td><td>1.49</td><td rowspan="2">6.2</td><td>1.56</td><td rowspan="2">2.99</td><td rowspan="2">6.2</td></tr>
<tr><td>2.99</td><td>3.13</td></tr>
<tr><td rowspan="2">SA 02</td><td>1.56</td><td rowspan="2">2.99</td><td rowspan="2">6.2</td><td>1.56</td><td rowspan="2">2.99</td><td rowspan="2">6.2</td></tr>
<tr><td>3.13</td><td>3.13</td></tr>
<tr><td rowspan="2">SE 01</td><td>0.39</td><td>0.75</td><td>1.55</td><td>0.78</td><td rowspan="2">1.49</td><td rowspan="2">3.1</td></tr>
<tr><td>0.78</td><td>1.49</td><td>3.11</td><td>1.56</td></tr>
<tr><td rowspan="2">SE 02</td><td rowspan="2">0.39</td><td>0.37</td><td rowspan="2">3.1</td><td rowspan="2">0.78</td><td rowspan="2">1.49</td><td>6.2</td></tr>
<tr><td>0.75</td><td>12.4</td></tr>
<tr><td rowspan="2">EF 01</td><td rowspan="2">12.5</td><td>5.97</td><td rowspan="2">ND $^{OD/L}$</td><td>12.5</td><td>5.97</td><td rowspan="2">12.4</td></tr>
<tr><td>11.9</td><td>25.0</td><td>11.9</td></tr>
<tr><td>EF 02</td><td>ND $^{OD/L}$</td><td>ND $^{OD/L}$</td><td>ND $^{OD/L}$</td><td>12.5</td><td>2.99</td><td>>198.9</td></tr>
<tr><td rowspan="2">EC</td><td rowspan="2">1.56</td><td>1.49</td><td rowspan="2">12.4</td><td>1.56</td><td>1.49</td><td>12.4</td></tr>
<tr><td>2.99</td><td>3.13</td><td>2.99</td><td>24.9</td></tr>
<tr><td rowspan="2">PA 01</td><td rowspan="2">3.13</td><td rowspan="2">2.99</td><td rowspan="2">12.4</td><td rowspan="2">3.13</td><td>2.99</td><td rowspan="2">12.4</td></tr>
<tr><td>5.97</td></tr>
<tr><td rowspan="2">PA 02</td><td rowspan="2">3.13</td><td>2.99</td><td rowspan="2">12.4</td><td rowspan="2">3.13</td><td>2.99</td><td rowspan="2">12.4</td></tr>
<tr><td>5.97</td><td>5.97</td></tr>
</table>

Legend: in case of a range of values, the lower value has been reported in the upper half of the cell and the higher value in the lower half. For Dadapin-1 the entire range of new and old values was considered.

Table S17. MIC, MBIC and MBC measurements of KSL in diluted medium

KSL - 20% MHB II		MIC µg/mL (µM)			MBIC µg/mL (µM)			MBC µg/mL (µM)		
		N. Exp.	values	range	N. Exp.	values	range	N. Exp.	values	range
SA	SA 00	3	1.95-1.95-1.95 (1.56-1.56-1.56)	1.95 (1.56)	3	1.95-1.95-1.95 (1.56-1.56-1.56) L	1.95 (1.56)	3	1.95-1.95-3.91 (1.56-1.56-3.13)	1.95-3.91 (1.56-3.13)
	SA 01	3	1.95-1.95-1.95 (1.56-1.56-1.56)	1.95 (1.56) L	3	1.95-1.95-1.95 (1.56-1.56-1.56) L	1.95 (1.56)	3	1.95-3.91-1.95 (1.56-3.13-1.56)	1.95-3.91 (1.56-3.13)
	SA 02	3	3.91-1.95-1.95 (3.13-1.56-1.56)	1.95-3.91 (1.56-3.13)	3	3.91-1.95-1.95 (3.13-1.56-1.56) L	1.95-3.91 (1.56-3.13)	3	3.91-1.95-3.91 (3.13-1.56-3.13)	1.95-3.91 (1.56-3.13)
SE	SE 01	3	0.98-0.98-0.49 (0.78-0.78-0.39)	0.49-0.78 (0.39-0.78)	3	0.98-0.98-0.98 (0.78-0.78-0.78) L	0.98 (0.78)	3	0.98-0.98-1.95 (0.78-0.78-1.56)	0.98-1.95 (0.78-1.56)
	SE 02	3	0.49-0.49-0.49 (0.39-0.39-0.39)	0.49 (0.39)	3	0.49-0.24-0.49 (0.39-0.19-0.39) L	0.24-0.49 (0.19-0.39)	3	0.98-0.98-0.98 (0.78-0.78-0.78)	0.98 (0.78)
EF	EF 01	3	15.63-15.63-15.63 (12.5-12.5-12.5)	15.63 (12.5)	3	7.81-3.91-7.81 (6.25-3.13-6.25) L	3.91-7.81 (3.13-6.25)	3	31.25-15.63-15.63 (25.0-12.5-12.5)	15.63-31.25 (12.5-25.0)
	EF 02	3	N.D.-N.D.-N.D. OD/L	N.D. OD/L	3	7.81-3.91-7.81 (6.25-3.13-6.25) L	3.91-7.81 (3.13-6.25)	3	15.63-15.63-15.63 (12.5-12.5-12.5)	15.63 (12.5)
EC	EC	3	1.95-1.95-1.95 (1.56-1.56-1.56) OD/L	1.95 (1.56) OD/L	3	1.95-1.95-1.95 (1.56-1.56-1.56) L	1.95 (1.56)	3	1.95-1.95-3.91 (1.56-1.56-3.13)	1.95-3.91 (1.56-3.13)
PA	PA 01	3	3.91-3.91-3.91 (3.13-3.13-3.13) OD/L	3.91 (3.13) OD/L	3	3.91-3.91-3.91 (3.13-3.13-3.13) L	3.91 (3.13)	3	3.91-3.91-3.91 (3.13-3.13-3.13)	3.91 (3.13)
	PA 02	3	3.91-3.91-3.91 (3.13-3.13-3.13) OD/L	3.91 (3.13) OD/L	3	3.91-3.91-3.91 (3.13-3.13-3.13) L	3.91 (3.13)	3	3.91-3.91-3.91 (3.13-3.13-3.13)	3.91 (3.13)

Legend: N. Exp., number of experiments performed; OD, measurements by OD reading; L, measurements by luminescence reading.

Table S18. Detailed view of all MIC, MBIC and MBC measurements of KSL-W in diluted medium

KSL - 20% MHB II		MIC μg/mL (μM)			MBIC μg/mL (μM)			MBC μg/mL (μM)		
		**N. Exp.**	**values**	**range**	**N. Exp.**	**values**	**range**	**N. Exp.**	**values**	**range**
SA	SA 00	3	1.95-1.95-3.91 (1.49-1.49-2.99)	1.95-3.91 (1.49-2.99)	3	1.95-1.95-1.95 (1.49-1.49-1.49)	1.95 (1.49)	3	3.91-3.91-3.91 (2.99-2.99-2.99)	3.91 (2.99)
	SA 01	3	1.95-1.95-3.91 (1.49-1.49-2.99)	1.95-3.91 (1.49-2.99)	3	3.91-1.95-1.95 (2.99-1.49-1.49)	1.95-3.91 (1.49-2.99)	3	3.91-3.91-3.91 (2.99-2.99-2.99)	3.91 (2.99)
	SA 02	3	3.91-3.91-3.91 (2.99-2.99-2.99)	3.91 (2.99)	3	3.91-3.91-3.91 (2.99-2.99-2.99)	3.91 (2.99)	3	3.91-3.91-3.91 (2.99-2.99-2.99)	3.91 (2.99)
SE	SE 01	3	0.98-0.98-1.95 (0.75-0.75-1.49)	0.98-1.95 (0.75-1.49)	3	1.95-1.95-1.95 (1.49-1.49-1.49)	1.95 (1.49)	3	1.95-1.95-1.95 (1.49-1.49-1.49)	1.95 (1.49)
	SE 02	4	0.98-0.49-0.49 (0.75-0.37-0.37)	0.49-0.98 (0.37-0.75)	3	0.24-0.24-0.49 (0.19-0.19-0.37)	0.24-0.49 (0.19-0.37)	3	1.95-1.95-1.95 (1.49-1.49-1.49)	1.95 (1.49)
EF	EF 01	3	7.81-7.81-15.63 (5.97-5.97-11.9)	7.81-15.63 (5.97-11.9)	4	3.91-3.91-3.91-7.81 (2.99-2.99-2.99-5.97)	3.91-7.81 (2.99-5.97)	4	7.81-7.81-15.63-15.63 (5.97-5.97-11.9-11.9)	7.81-15.63 (5.97-11.9)
	EF 02	3	N.D.-N.D.-N.D. OD/L	N.D. OD/L	3	3.91-3.91-3.91 (2.99-2.99-2.99)	3.91 (2.99)	3	3.91-3.91-3.91 (2.99-2.99-2.99)	3.91 (2.99)
EC	EC	3	3.91-1.95-3.91 (2.99-1.49-2.99) OD/L	1.95-3.91 (1.49-2.99) OD/L	3	1.95-1.95-1.95 (1.49-1.49-1.49)	1.95 (1.49)	3	3.91-1.95-3.91 (2.99-1.49-2.99)	1.95-3.91 (1.49-2.99)
PA	PA 01	3	3.91-3.91-3.91 (2.99-2.99-2.99) OD/L	3.91 (2.99)	3	3.91-3.91-3.91 (2.99-2.99-2.99)	3.91 (2.99)	3	3.91-7-81-3.91 (2.99-5.97-2.99)	3.91-7.81 (2.99-5.97)
	PA 02	5	7.81-3.91-7.81-3.91-7.81 (5.97-2.99-5.97-2.99-5.97) OD/L	3.91-7.81 (2.99-5.97)	5	7.81-3.91-7.81-3.91-7.81 (5.97-2.99-5.97-2.99-5.97)	3.91-7.81 (2.99-5.97)	5	7.81-3.91-7.81-3.91-7.81 (5.97-2.99-5.97-2.99-5.97)	3.91-7.81 (2.99-5.97)

Legend: N. Exp., number of experiments performed; OD, measurements by OD reading; L, measurements by luminescence reading.

Table S19. MIC, MBIC and MBC new measurements of Dadapin-1 in diluted medium performed in the present study either to test new strains or for re-testing past strains

Dadapin-1 - 20% MHB II		MIC µg/mL (µM)			MBIC µg/mL (µM)			MBC µg/mL (µM)		
		**N. Exp.**	**Values**	**Range**	**N. Exp.**	**Values**	**Range**	**N. Exp.**	**Values**	**Range**
SA	SA 00	2	15.63-15.63 (6.2-6.2)	15.63 (6.2)	2	7.81-7.81 (3.1-3.1)	7.81 (3.1)	2	31.25-15.63 (12.4-6.2)	15.63-31.25 (6.2-12.4)
	SA 01	2	15.63-15.63 (6.2-6.2)	15.63 (6.2)	2	7.81-15.63 (3.1-6.2)	7.81-15.63 (3.1-6.2)	2	15.63-15.63 (6.2-6.2)	15.63 (6.2)
	SA 02	3	15.63-15.63-15.63 (6.2-6.2-6.2)	15.63 (6.2)	3	7.81-15.63-15.63 (3.1-6.2-6.2)	7.81-15.63 (3.1- 6.2)	3	15.63-15.63-15.63 (6.2-6.2-6.2)	15.63 (6.2)
SE	SE 01	2	3.91-7.81 (3.1-1.55)	3.91-7.81 (1.55-3.1)	2	7.81-3.91 (3.1-1.55)	3.91-7.81 (1.55-3.1)	2	7.81-7.81 (3.1-3.1)	7.81 (3.1)
	SE 02	3	7.81-N.D.-N.D. (3.1) [L]	7.81 (3.1)	3	7.81-7.81-7.81 (3.1-3.1-3.1)	7.81 (3.1)	3	15.63-15.63-15.63 (6.2-6.2-6.2)	15.63 (6.2)
EF	EF 01	3	N.D.-N.D.-N.D. [OD/L]	N.D. [OD/L]	3	15.63-15.63-31.25 (6.2-6.2-12.4)	15.63-31.25 (6.2-12.4)	3	31.25-31.25-31.25 (12.4-12.4-12.4)	31.25 (12.4)
	EF 02	4	N.D.-N-D.-N.D.-N.D. [OD/L]	N.D. [OD/L]	4	31.25-31.25-15.63-15.63 (12.4-12.4-6.2-6.2)	15.63-31.25 (6.2-12.4)	2	>500->500 (>198.9->198.9)	>500 (>198.9)
EC	EC	2	31.25-31.25 (12.4-12.4)	31.25 (12.4)	2	31.25-15.63 (12.4-6.2)	15.63-31.25 (6.2-12.4)	2	31.25-62.5 (12.4-24.9)	31.25-62.5 (12.4-24.9)
PA	PA 01	2	31.25-31.25 (12.4-12.4)	31.25 (12.4)	2	31.25-31.25 (12.4-12.4)	31.25 (12.4)	2	31.25-31.25 (12.4-12.4)	31.25 (12.4)
	PA 02	3	31.25-31.25-31.25 (12.4-12.4-12.4)	31.25 (12.4)	3	31.25-31.25-15.63 (12.4-12.4-6.2)	15.63-31.25 (6.2-12.4)	3	31.25-31.25-31.25 (12.4-12.4-12.4)	31.25 (12.4)

Legend: N. Exp., number of experiments performed; OD, measurements by OD reading; L, measurements by luminescence reading.

Table S20. MBC/MIC ratio in diluted MHB II medium.

Strain	MBC/MIC		
	**KSL**	**KSL-W**	**Dadapin-1**
SA 00	1–2	1–2	1 [n]
SA 01	1–2	1–2	1 [n]
SA 02	1	1	1 [n]
SE 01	2	1–2	1–2 [n]
SE 02	2	**2–4**	2 [n]
EF 01	1–2	1	-
EF 02	-	-	-
EC	1–2	1	1–2 [n]
PA 01	1	1–2	1 [n]
PA 02	1	1	1 [n]

Legend: values greater than two are in bold. [n] new Dadapin-1 results from the present study. Note that, for the earlier published data, MBC values were ascertained by plating 100 µl on MHA plates as previously described [21], instead of 10 µl as in the present study.

Table S21. Biofilm-inhibitory activity of AMPs (MBIC).

Bacterial strain	MBIC [µM]					
	Diluted MHB II			Undiluted MHB II		
	KSL	KSL-W	Dadapin-1	KSL	KSL-W	Dadapin-1
SA 00	1.56	1.49	(3.11)	12.5	5.97	99.4-198.9 (>198.9)
SA 01	1.56	1.49-2.99<br>4LR: 2.99	3.11-6.22 (6.22)	25.0-50.0<br>2LR: 50.0	3LR: 23.9	(>198.9)
SA 02	1.56-3.13<br>3LR: 3.13	3LR: 2.99	3.1-6.22	2LR: 25.0	3LR: 11.9	198.9
SE 01	2LR: 0.78	3LR: 1.49	1.55-3.11 (3.11)	3LR: 3.13	1.49-2.99	(198.9)
SE 02	0.19-0.39	0.19-0.37	(3.11)	2LR: 1.56	0.37-1.49<br>2LR: 1.49	>198.9 (99.4)
EF 01	3.13-6.25	2.99-5.97	6.22-12.43<br>3LR: 12.43	100	11.9-23.9<br>3LR: 23.9	>198.9
EF 02	3.13-6.25<br>2LR: 6.25	2LR: 2.99	6.22-12.43	200	11.9-23.9<br>2LR: 23.9	>198.9
EC	3LR: 1.56	1.49<br>3LR: 2.99	6.22-12.43 (12.43)<br>2LR: 12.43	3LR: 6.25	3LR: 2.99	(>198.9)
PA 01	3LR: 3.13	2LR: 2.99	3LR: (12.43)	50.0-100	2LR: 23.9	(>198.9)
PA 02	2LR: 3.13	2.99-5.97<br>2LR: 5.97	6.22-12.43<br>3LR: 12.43	50.0-100<br>3LR: 100	3LR: 23.9	>198.9

MBIC values (in µM) obtained in diluted and undiluted medium. For Dadapin-1 values between brackets indicate earlier published results [21] and new results from the present investigation are displayed only when differing. Legend: LR, Log_{10} reduction in bacterial metabolism for a given MBIC. Green cells indicate the lowest MIC and MBC values observed for a given bacterial strain across the three peptides. Grey cells indicate the same MIC or MBC values (or partially overlapping ranges of values) for a given bacterial strain across the different peptides. Orange cells indicate the highest MIC and MBC.

Table S22. Heatmap of MBIC values obtained in diluted (20% MHB) and undiluted (MHB) medium.

Bacterial strain	MBIC (µM)					
	MHB II			20% MHB II		
	KSL	KSL-W	Dadapin-1	KSL	KSL-W	Dadapin-1
SA 00	12.5	5.97	99.4<br>198.9<br>>198.9	1.56	1.49	3.1
SA 01	25.0<br>50.0	23.9	>198.9	1.56	1.49<br>2.99	3.1<br>6.2
SA 02	25.00	11.9	198.9	1.56<br>3.13	2.99	3.1<br>6.2
SE 01	3.13	1.49<br>2.99	198.9	0.78	1.49	1.6<br>3.1
SE 02	1.56	0.37<br>1.49	99.4<br>>198.9	0.19<br>0.39	0.19<br>0.37	3.1
EF 01	100	11.9<br>23.9	>198.9	3.13<br>6.25	2.99<br>5.97	6.2<br>12.4
EF 02	200	11.9<br>23.9	>198.9	3.13<br>6.25	2.99	6.2<br>12.4
EC	6.25	2.99	>198.9	1.56	1.49	6.2<br>12.4
PA 01	50.0<br>100	23.9	>198.9	3.13	2.99	12.4
PA 02	50.0<br>100	23.9	>198.9	3.13	2.99<br>5.97	6.2<br>12.4

Legend: in case of a range of values, the lower value has been reported in the upper half of the cell and the higher value in the lower half. For Dadapin-1 the range considers new and old values.

Table S23. Two-way ANOVA analysis of Log_2 (MBIC) of KSL and KSL-W in undiluted (100%) and diluted (20%) MHB II.

Table Analyzed	MBIC				
Two-way ANOVA	Ordinary				
Alpha	0.05				
Source of Variation	% of total variation	P value	P value summary	Significant?	
Interaction	7.543	<0.0001	****	Yes	
Row Factor	45.64	<0.0001	****	Yes	
Column Factor	46.02	<0.0001	****	Yes	
ANOVA table	SS (Type III)	DF	MS	F (DFn, DFd)	P value
Interaction	45.88	27	1.699	F (27, 85) = 11.21	P<0.0001
Row Factor	277.6	9	30.84	F (9, 85) = 203.5	P<0.0001
Column Factor	279.9	3	93.31	F (3, 85) = 615.8	P<0.0001
Residual	12.88	85	0.1515		
Data summary					
Number of columns (Column Factor)	4				
Number of rows (Row Factor)	10				
Number of values	125				

Table S24. Tukey's multiple comparisons test of Log_2 (MBIC) of KSL and KSL-W in undiluted (100%) and diluted (20%) MHB II.

Within each row, compare columns (simple effects within rows)

Number of families	10
Number of comparisons per family	6
Alpha	0.05

Tukey's multiple comparisons test	Predicted (LS) mean diff.	95.00% CI of diff.	Below thresh-old?	Summary	Adjusted P Value
SA 00					
Log2 KSL 100% vs. Log2 KSL-W 100%	1	0.1671 to 1.833	Yes	*	0.012
Log2 KSL 100% vs. Log2 KSL 20%	3.01	2.177 to 3.843	Yes	****	<0.0001
Log2 KSL 100% vs. Log2 KSL-W 20%	3.01	2.177 to 3.843	Yes	****	<0.0001
Log2 KSL-W 100% vs. Log2 KSL 20%	2.01	1.177 to 2.843	Yes	****	<0.0001
Log2 KSL-W 100% vs. Log2 KSL-W 20%	2.01	1.177 to 2.843	Yes	****	<0.0001
Log2 KSL 20% vs. Log2 KSL-W 20%	-1.11E-15	-0.8329 to 0.8329	No	ns	>0.9999
SA 01					
Log2 KSL 100% vs. Log2 KSL-W 100%	0.6667	-0.1662 to 1.500	No	ns	0.1622
Log2 KSL 100% vs. Log2 KSL 20%	4.677	3.844 to 5.510	Yes	****	<0.0001
Log2 KSL 100% vs. Log2 KSL-W 20%	4.34	3.507 to 5.173	Yes	****	<0.0001
Log2 KSL-W 100% vs. Log2 KSL 20%	4.01	3.177 to 4.843	Yes	****	<0.0001
Log2 KSL-W 100% vs. Log2 KSL-W 20%	3.673	2.840 to 4.506	Yes	****	<0.0001
Log2 KSL 20% vs. Log2 KSL-W 20%	-0.3367	-1.170 to 0.4962	No	ns	0.7151
SA 02					
Log2 KSL 100% vs. Log2 KSL-W 100%	1	0.1671 to 1.833	Yes	*	0.012
Log2 KSL 100% vs. Log2 KSL 20%	3.673	2.840 to 4.506	Yes	****	<0.0001

Log2 KSL 100% vs. Log2 KSL-W 20%	3	2.167 to 3.833	Yes	****	<0.0001
Log2 KSL-W 100% vs. Log2 KSL 20%	2.673	1.840 to 3.506	Yes	****	<0.0001
Log2 KSL-W 100% vs. Log2 KSL-W 20%	2	1.167 to 2.833	Yes	****	<0.0001
Log2 KSL 20% vs. Log2 KSL-W 20%	-0.6733	-1.506 to 0.1596	No	ns	0.1555
SE 01					
Log2 KSL 100% vs. Log2 KSL-W 100%	0.3367	-0.4962 to 1.170	No	ns	0.7151
Log2 KSL 100% vs. Log2 KSL 20%	2	1.167 to 2.833	Yes	****	<0.0001
Log2 KSL 100% vs. Log2 KSL-W 20%	1.01	0.1771 to 1.843	Yes	*	0.0109
Log2 KSL-W 100% vs. Log2 KSL 20%	1.663	0.8304 to 2.496	Yes	****	<0.0001
Log2 KSL-W 100% vs. Log2 KSL-W 20%	0.6733	-0.1596 to 1.506	No	ns	0.1555
Log2 KSL 20% vs. Log2 KSL-W 20%	-0.99	-1.823 to -0.1571	Yes	*	0.0131
SE 02					
Log2 KSL 100% vs. Log2 KSL-W 100%	1.243	0.4634 to 2.022	Yes	***	0.0004
Log2 KSL 100% vs. Log2 KSL 20%	2.333	1.500 to 3.166	Yes	****	<0.0001
Log2 KSL 100% vs. Log2 KSL-W 20%	2.677	1.844 to 3.510	Yes	****	<0.0001
Log2 KSL-W 100% vs. Log2 KSL 20%	1.091	0.3117 to 1.870	Yes	**	0.0024
Log2 KSL-W 100% vs. Log2 KSL-W 20%	1.434	0.6550 to 2.213	Yes	****	<0.0001
Log2 KSL 20% vs. Log2 KSL-W 20%	0.3433	-0.4896 to 1.176	No	ns	0.7025
EF 01					
Log2 KSL 100% vs. Log2 KSL-W 100%	2.667	1.834 to 3.500	Yes	****	<0.0001
Log2 KSL 100% vs. Log2 KSL 20%	4.333	3.500 to 5.166	Yes	****	<0.0001
Log2 KSL 100% vs. Log2 KSL-W 20%	4.75	3.971 to 5.529	Yes	****	<0.0001
Log2 KSL-W 100% vs. Log2 KSL 20%	1.667	0.8338 to 2.500	Yes	****	<0.0001
Log2 KSL-W 100% vs. Log2 KSL-W 20%	2.083	1.304 to 2.862	Yes	****	<0.0001
Log2 KSL 20% vs. Log2 KSL-W 20%	0.4167	-0.3625 to 1.196	No	ns	0.5019

EF 02					
Log2 KSL 100% vs. Log2 KSL-W 100%	3.75	2.971 to 4.529	Yes	****	<0.0001
Log2 KSL 100% vs. Log2 KSL 20%	5.333	4.500 to 6.166	Yes	****	<0.0001
Log2 KSL 100% vs. Log2 KSL-W 20%	6	5.167 to 6.833	Yes	****	<0.0001
Log2 KSL-W 100% vs. Log2 KSL 20%	1.583	0.8042 to 2.362	Yes	****	<0.0001
Log2 KSL-W 100% vs. Log2 KSL-W 20%	2.25	1.471 to 3.029	Yes	****	<0.0001
Log2 KSL 20% vs. Log2 KSL-W 20%	0.6667	-0.1662 to 1.500	No	ns	0.1622
EC					
Log2 KSL 100% vs. Log2 KSL-W 100%	1	0.1671 to 1.833	Yes	*	0.012
Log2 KSL 100% vs. Log2 KSL 20%	2.01	1.177 to 2.843	Yes	****	<0.0001
Log2 KSL 100% vs. Log2 KSL-W 20%	2.01	1.177 to 2.843	Yes	****	<0.0001
Log2 KSL-W 100% vs. Log2 KSL 20%	1.01	0.1771 to 1.843	Yes	*	0.0109
Log2 KSL-W 100% vs. Log2 KSL-W 20%	1.01	0.1771 to 1.843	Yes	*	0.0109
Log2 KSL 20% vs. Log2 KSL-W 20%	0	-0.8329 to 0.8329	No	ns	>0.9999
PA 01					
Log2 KSL 100% vs. Log2 KSL-W 100%	1.667	0.8338 to 2.500	Yes	****	<0.0001
Log2 KSL 100% vs. Log2 KSL 20%	4.667	3.834 to 5.500	Yes	****	<0.0001
Log2 KSL 100% vs. Log2 KSL-W 20%	4.667	3.834 to 5.500	Yes	****	<0.0001
Log2 KSL-W 100% vs. Log2 KSL 20%	3	2.167 to 3.833	Yes	****	<0.0001
Log2 KSL-W 100% vs. Log2 KSL-W 20%	3	2.167 to 3.833	Yes	****	<0.0001
Log2 KSL 20% vs. Log2 KSL-W 20%	-1.55E-15	-0.8329 to 0.8329	No	ns	>0.9999
PA 02					
Log2 KSL 100% vs. Log2 KSL-W 100%	1.333	0.5004 to 2.166	Yes	***	0.0004
Log2 KSL 100% vs. Log2 KSL 20%	4.333	3.500 to 5.166	Yes	****	<0.0001
Log2 KSL 100% vs. Log2 KSL-W 20%	3.733	2.988 to 4.478	Yes	****	<0.0001
Log2 KSL-W 100% vs. Log2 KSL 20%	3	2.167 to 3.833	Yes	****	<0.0001

Log2 KSL-W 100% vs. Log2 KSL-W 20%	2.4	1.655 to 3.145	Yes	****	<0.0001
Log2 KSL 20% vs. Log2 KSL-W 20%	-0.6	-1.345 to 0.1450	No	ns	0.158

Test details	Predicted (LS) mean 1	Predicted (LS) mean 2	Predicted (LS) mean diff.	SE of diff.	N1	N2	q	DF
SA 00								
Log2 KSL 100% vs. Log2 KSL-W 100%	3.97	2.97	1	0.3178	3	3	4.45	85
Log2 KSL 100% vs. Log2 KSL 20%	3.97	0.96	3.01	0.3178	3	3	13.39	85
Log2 KSL 100% vs. Log2 KSL-W 20%	3.97	0.96	3.01	0.3178	3	3	13.39	85
Log2 KSL-W 100% vs. Log2 KSL 20%	2.97	0.96	2.01	0.3178	3	3	8.944	85
Log2 KSL-W 100% vs. Log2 KSL-W 20%	2.97	0.96	2.01	0.3178	3	3	8.944	85
Log2 KSL 20% vs. Log2 KSL-W 20%	0.96	0.96	-1.11E-15	0.3178	3	3	4,940e-015	85
SA 01								
Log2 KSL 100% vs. Log2 KSL-W 100%	5.637	4.97	0.6667	0.3178	3	3	2.966	85
Log2 KSL 100% vs. Log2 KSL 20%	5.637	0.96	4.677	0.3178	3	3	20.81	85
Log2 KSL 100% vs. Log2 KSL-W 20%	5.637	1.297	4.34	0.3178	3	3	19.31	85
Log2 KSL-W 100% vs. Log2 KSL 20%	4.97	0.96	4.01	0.3178	3	3	17.84	85
Log2 KSL-W 100% vs. Log2 KSL-W 20%	4.97	1.297	3.673	0.3178	3	3	16.34	85
Log2 KSL 20% vs. Log2 KSL-W 20%	0.96	1.297	-0.3367	0.3178	3	3	1.498	85
SA 02								
Log2 KSL 100% vs. Log2 KSL-W 100%	4.97	3.97	1	0.3178	3	3	4.45	85
Log2 KSL 100% vs. Log2 KSL 20%	4.97	1.297	3.673	0.3178	3	3	16.34	85
Log2 KSL 100% vs. Log2 KSL-W 20%	4.97	1.97	3	0.3178	3	3	13.35	85
Log2 KSL-W 100% vs. Log2 KSL 20%	3.97	1.297	2.673	0.3178	3	3	11.9	85
Log2 KSL-W 100% vs. Log2 KSL-W 20%	3.97	1.97	2	0.3178	3	3	8.899	85

Log2 KSL 20% vs. Log2 KSL-W 20%	1.297	1.97	-0.6733	0.3178	3	3	2.996	85
SE 01								
Log2 KSL 100% vs. Log2 KSL-W 100%	1.97	1.633	0.3367	0.3178	3	3	1.498	85
Log2 KSL 100% vs. Log2 KSL 20%	1.97	-0.03	2	0.3178	3	3	8.899	85
Log2 KSL 100% vs. Log2 KSL-W 20%	1.97	0.96	1.01	0.3178	3	3	4.494	85
Log2 KSL-W 100% vs. Log2 KSL 20%	1.633	-0.03	1.663	0.3178	3	3	7.401	85
Log2 KSL-W 100% vs. Log2 KSL-W 20%	1.633	0.96	0.6733	0.3178	3	3	2.996	85
Log2 KSL 20% vs. Log2 KSL-W 20%	-0.03	0.96	-0.99	0.3178	3	3	4.405	85
SE 02								
Log2 KSL 100% vs. Log2 KSL-W 100%	0.96	-0.2825	1.243	0.2973	3	4	5.91	85
Log2 KSL 100% vs. Log2 KSL 20%	0.96	-1.373	2.333	0.3178	3	3	10.38	85
Log2 KSL 100% vs. Log2 KSL-W 20%	0.96	-1.717	2.677	0.3178	3	3	11.91	85
Log2 KSL-W 100% vs. Log2 KSL 20%	-0.2825	-1.373	1.091	0.2973	4	3	5.189	85
Log2 KSL-W 100% vs. Log2 KSL-W 20%	-0.2825	-1.717	1.434	0.2973	4	3	6.822	85
Log2 KSL 20% vs. Log2 KSL-W 20%	-1.373	-1.717	0.3433	0.3178	3	3	1.528	85
EF 01								
Log2 KSL 100% vs. Log2 KSL-W 100%	6.97	4.303	2.667	0.3178	3	3	11.87	85
Log2 KSL 100% vs. Log2 KSL 20%	6.97	2.637	4.333	0.3178	3	3	19.28	85
Log2 KSL 100% vs. Log2 KSL-W 20%	6.97	2.22	4.75	0.2973	3	4	22.59	85
Log2 KSL-W 100% vs. Log2 KSL 20%	4.303	2.637	1.667	0.3178	3	3	7.416	85
Log2 KSL-W 100% vs. Log2 KSL-W 20%	4.303	2.22	2.083	0.2973	3	4	9.91	85
Log2 KSL 20% vs. Log2 KSL-W 20%	2.637	2.22	0.4167	0.2973	3	4	1.982	85
EF 02								
Log2 KSL 100% vs. Log2 KSL-W 100%	7.97	4.22	3.75	0.2973	3	4	17.84	85
Log2 KSL 100% vs. Log2 KSL 20%	7.97	2.637	5.333	0.3178	3	3	23.73	85

Log2 KSL 100% vs. Log2 KSL-W 20%	7.97	1.97	6	0.3178	3	3	26.7	85
Log2 KSL-W 100% vs. Log2 KSL 20%	4.22	2.637	1.583	0.2973	4	3	7.532	85
Log2 KSL-W 100% vs. Log2 KSL-W 20%	4.22	1.97	2.25	0.2973	4	3	10.7	85
Log2 KSL 20% vs. Log2 KSL-W 20%	2.637	1.97	0.6667	0.3178	3	3	2.966	85
EC								
Log2 KSL 100% vs. Log2 KSL-W 100%	2.97	1.97	1	0.3178	3	3	4.45	85
Log2 KSL 100% vs. Log2 KSL 20%	2.97	0.96	2.01	0.3178	3	3	8.944	85
Log2 KSL 100% vs. Log2 KSL-W 20%	2.97	0.96	2.01	0.3178	3	3	8.944	85
Log2 KSL-W 100% vs. Log2 KSL 20%	1.97	0.96	1.01	0.3178	3	3	4.494	85
Log2 KSL-W 100% vs. Log2 KSL-W 20%	1.97	0.96	1.01	0.3178	3	3	4.494	85
Log2 KSL 20% vs. Log2 KSL-W 20%	0.96	0.96	0	0.3178	3	3	0	85
PA 01								
Log2 KSL 100% vs. Log2 KSL-W 100%	6.637	4.97	1.667	0.3178	3	3	7.416	85
Log2 KSL 100% vs. Log2 KSL 20%	6.637	1.97	4.667	0.3178	3	3	20.76	85
Log2 KSL 100% vs. Log2 KSL-W 20%	6.637	1.97	4.667	0.3178	3	3	20.76	85
Log2 KSL-W 100% vs. Log2 KSL 20%	4.97	1.97	3	0.3178	3	3	13.35	85
Log2 KSL-W 100% vs. Log2 KSL-W 20%	4.97	1.97	3	0.3178	3	3	13.35	85
Log2 KSL 20% vs. Log2 KSL-W 20%	1.97	1.97	-1.55E-15	0.3178	3	3	6,916e-015	85
PA 02								
Log2 KSL 100% vs. Log2 KSL-W 100%	6.303	4.97	1.333	0.3178	3	3	5.933	85
Log2 KSL 100% vs. Log2 KSL 20%	6.303	1.97	4.333	0.3178	3	3	19.28	85
Log2 KSL 100% vs. Log2 KSL-W 20%	6.303	2.57	3.733	0.2843	3	5	18.57	85
Log2 KSL-W 100% vs. Log2 KSL 20%	4.97	1.97	3	0.3178	3	3	13.35	85
Log2 KSL-W 100% vs. Log2 KSL-W 20%	4.97	2.57	2.4	0.2843	3	5	11.94	85
Log2 KSL 20% vs. Log2 KSL-W 20%	1.97	2.57	-0.6	0.2843	3	5	2.985	85

Table S25. Comparison of current results with data on KSL available from past studies

KSL	Past studies					Present study	
	MIC (µg/ml)	MBC (µg/ml)	MBIC (µg/ml)	Medium	Ref.	MIC (MHB II)	MIC (20% MHB II)
SA 00	3.12	NP	NP	AM3	13	31.25-62.5	1.95-3.91
	3.12	NP	NP	AM3	14		
S. aureus	12.5	NP	NP	AM3	13		
S. aureus 1550	22	NP	NP	AM3	14		
S. epidermidis ATCC 12228	0.78	NP	NP	AM3	13	0.98-3.91	0.49-0.98
E. coli ATCC 2592	3.12	NP	NP	AM3	13	7.81	1.95
	10	NP	NP	AM3	14		
P. aeruginosa ATCC 9027	1.56	NP	NP	AM3	13	62.5-125	3.91
	5	NP	NP	AM3	14		

Legend: NP, not performed.

Table S26. Comparison of current results with data on KSL-W available from past studies

KSL-W	Past studies					Present study	
	MIC (µg/ml)	MBC (µg/ml)	MBIC (µg/ml)	Medium	Ref.	MIC (MHB II)	MIC (20% MHB II)
S. aureus PCM 2054	25	NP	NP	MHB	22	15.63-31.25	1.95-3.91
S. epidermidis PCM 2118	7.8	NP	NP	MHB	22	0.49-3.91	0.98-1.95
E. coli PCM 2057	7.8	NP	NP	MHB	22	7.81	1.95-3.91
P. aeruginosa PCM 499	25	NP	NP	MHB	22	31.25	3.91-7.81

Legend: NP, not performed.

Spectra and chromatograms of the different lots of peptides

Peptide: Dadapin-1
Lot n°: P210416-GB889991
Producer: GenicBio

GenicBio Limited

Tel: +86-(0)21-3762 1270 | Fax: +86(0)21-6085 3385 | Email: service@genicbio.com

HPLC Result

Product Name	Dadapin-1		
Lot. No.:	P210416-GB889991		
Column:	4.6×250mm, Kromasil 100-5 C18		
Solvent A	0.1% trifluoroacetic in 100% acetonitrile		
Solvent B	0.1% trifluoroacetic in 100% water		
Gradient		A	B
	0.01min	18%	82%
	25.00min	43%	57%
	25.10min	100%	0%
	30.00min	Stop	
Flow Rate	1.0 ml/min		
Wavelength:	220nm		
Volume:	5μl		

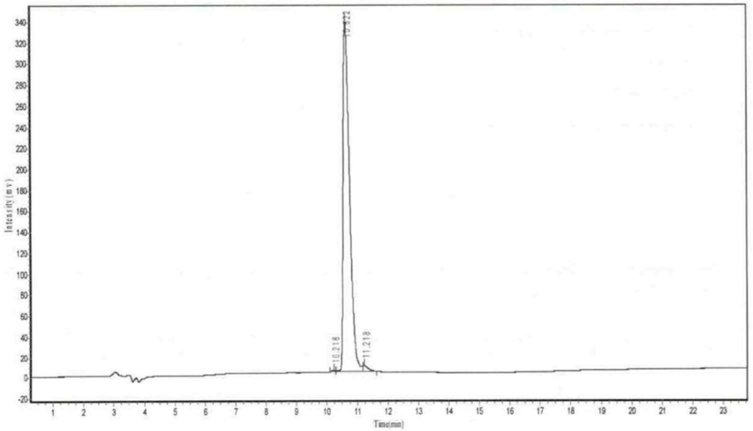

Peak	Time	Height	Area	Conc.
1	10.218	1191.543	9235.632	0.1834
2	10.622	336604.219	4956876.500	98.4187
3	11.218	5900.917	70407.953	1.3979
Total				100.0000

GenicBio Limited

Tel: +86-(0)21-3762 1270 | Fax: +86(0)21-6085 3385 | Email: service@genicbio.com

Mass Spectrometry

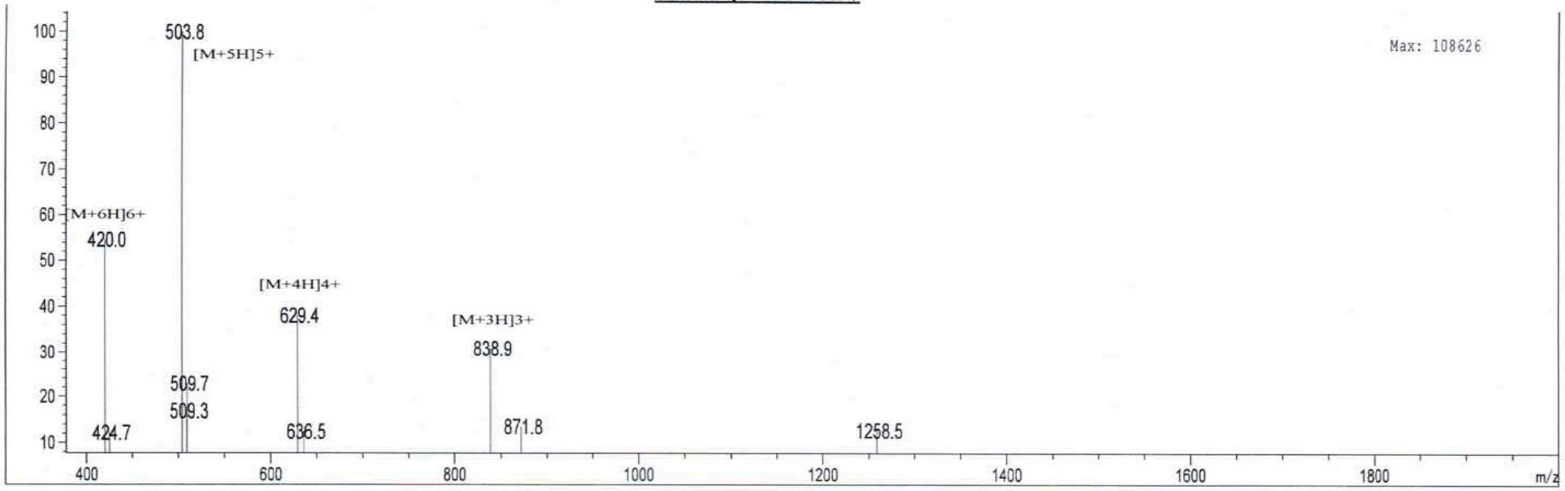

Sample Description		Instrument	Agilent-6125B		
Analyzed date:	2021-05-07	Probe:	ESI	Probe Bias:	+4.5kv
Analyst:	Wang	Nebulizer Gas Flow:	1.5L/min	Detector:	1.5kv
Sample:	Dadapin-1	CDL:	-20.0v	T. Flow:	0.2ml/min
M.W.:	2513.91	CDL Temp.:	250℃	B. Conc:	50%H2O/50%ACN
Lot. No.:	P210416-GB889991	Block Temp.:	200℃		

Peptide: Dadapin-1
Lot n°: P220627-JQ1000384
Producer: ProteoGenix

Sample: GA-23
Lot. No.: P220627-JQ1000384
Column: Kromasil 100-5C18, 4.6*250mm, 5μm
Solvent A: A: 0.1% Trifluoroacetic Acid in 100% Acetonitrile
Solvent B: B: 0.1% Trifluoroacetic Acid in 100% Water
Gradient:

	A	B
0.0min	17%	83%
25.0min	42%	58%
25.1min	100%	0%
30.0min	Stop	

Volume: 10μl
Wavelength: 220nm
Flow rate: 1.0ml/min

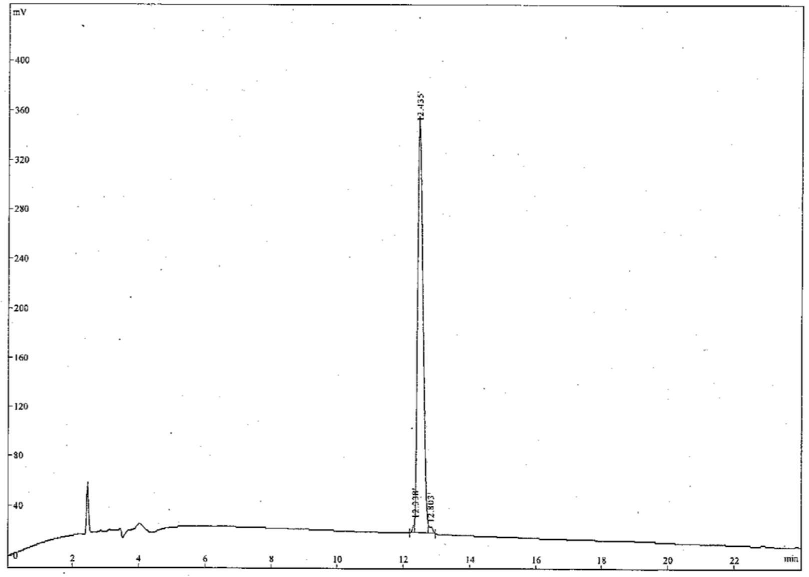

Rank	Time	Conc.	Area	Height
1	12.338	0.7683	29867	11224
2	12.435	98.04	3811224	338178
3	12.803	1.198	46560	5716
Total		100	3887651	355118

MASS SPECTROMETRY REPORT

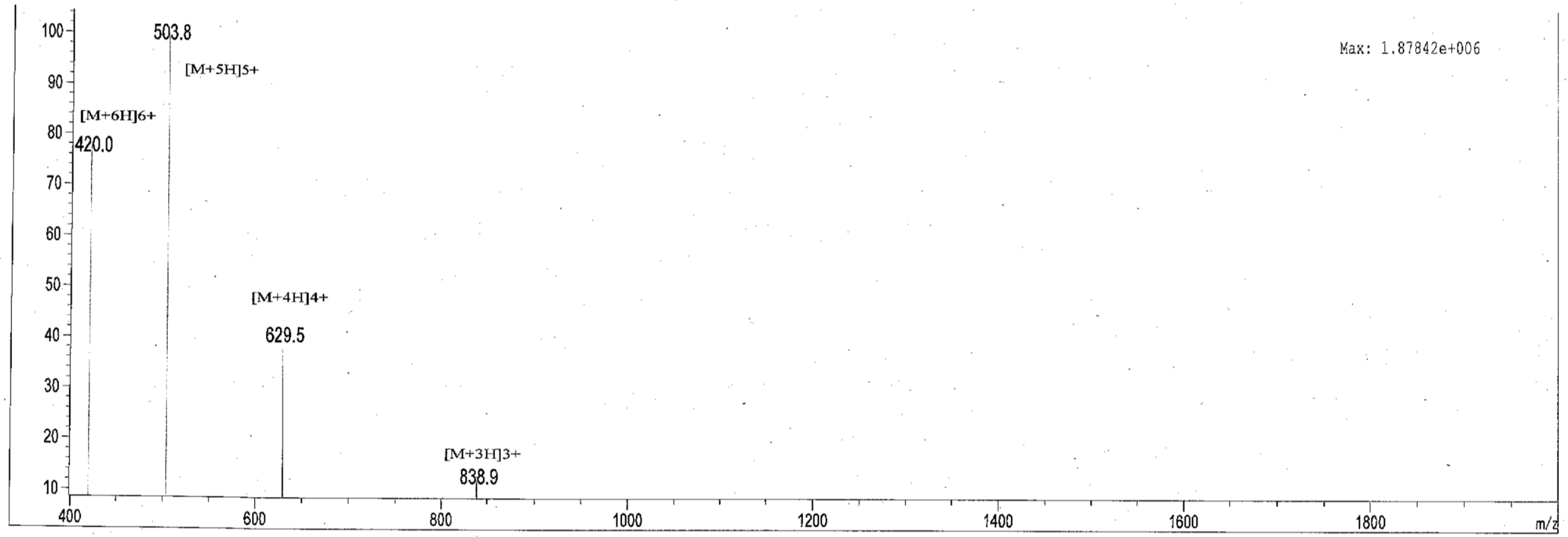

Sample Information		Probe:	ESI	Probe Bias:	+4.5kv
Injection Volume	: 1.00 μ l	Nebulizer Gas Flow:	1.5L/min	Detector:	1.5kv
Sample:	GA-23	CDL:	-20.0v	T. Flow:	0.2ml/min
M.W.:	2513.91	CDL Temp.:	250 °C	B. Conc.:	50%H2O/50%ACN
Lot. No.:	P220627-JQ1000384	Block Temp.:	200 °C		

Peptide: Dadapin-1

Sample: Dadapin-1 GA-23

Lot. No.: P221129-YS1000383

Column: 4.6×250mm,ChromCore 120 C18 5u

Lot n°: P221129-YS1000383
Producer: ProteoGenix

MASS SPECTROMETRY REPORT

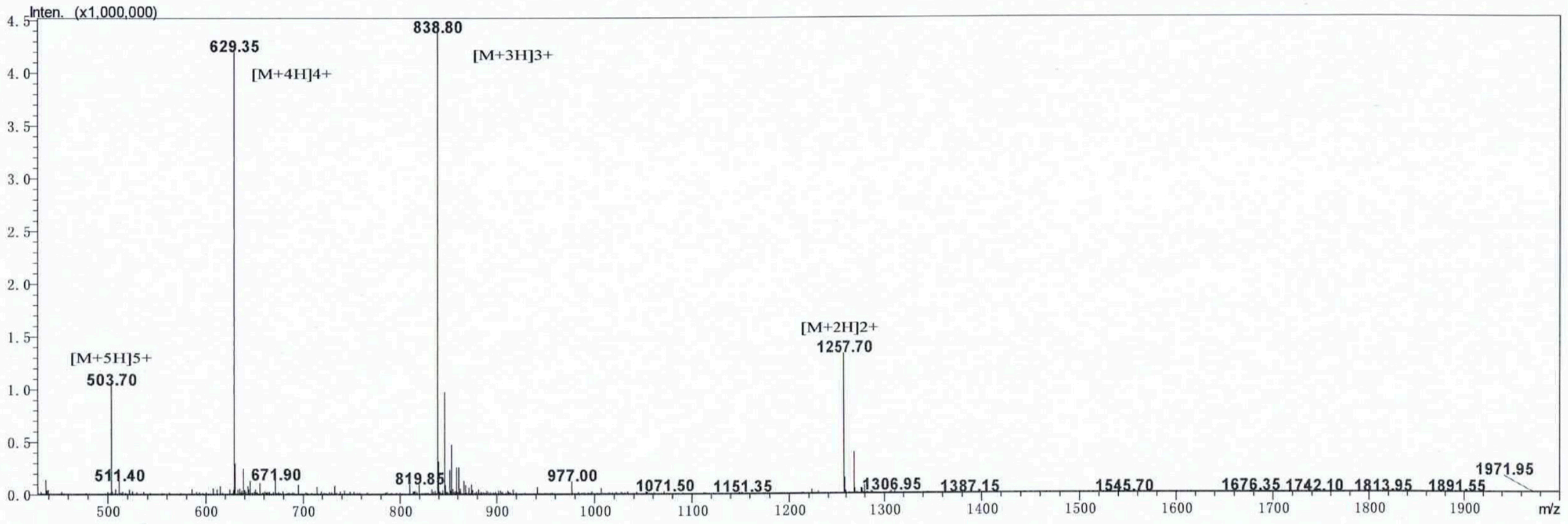

Sample Information

Injection Volume : 1.00 μ l

Sample: GA-23

M.W.: 2513.91

Lot. No.: P221129-YS1000383

Probe: ESI

Nebulizer Gas Flow: 1.5L/min

CDL: -20.0v

CDL Temp.: 250 °C

Block Temp.: 400 °C

Probe Bias: +4.5kv

Detector: 1.2kv

T. Flow: 0.2ml/min

B. Conc.: 50%H2O/50%ACN

Peptide: KSL
Lot n°: P220627-JQ1000387
Producer: ProteoGenix

HPLC REPORT

Sample: KK-10
Analyst: HCM
Lot. No.: P220627-JQ1000387
Column: 4.6×250mm, SinoChrom ODS-BP
Solvent A: A: 0.1% Trifluoroacetic Acid in 100% Acetonitrile
Solvent B: B: 0.1% Trifluoroacetic Acid in 100% Water

Gradient:

	A	B
0.0min	15%	85%
25.0min	40%	60%
25.1min	100%	0%
30.0min	Stop	

Volume: 5μl
Wavelength: 220nm
Flow rate: 1.0ml/min

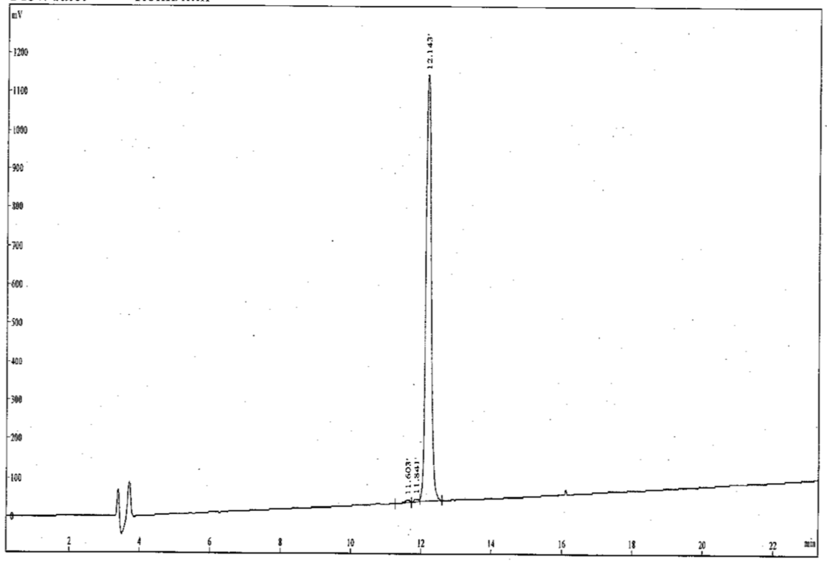

Rank	Time	Conc.	Area	Height
1	11.603	0.5088	53700	5922
2	11.841	0.6439	67962	7822
3	12.143	98.85	10433256	1102399
Total		100	10554918	1116143

MASS SPECTROMETRY REPORT

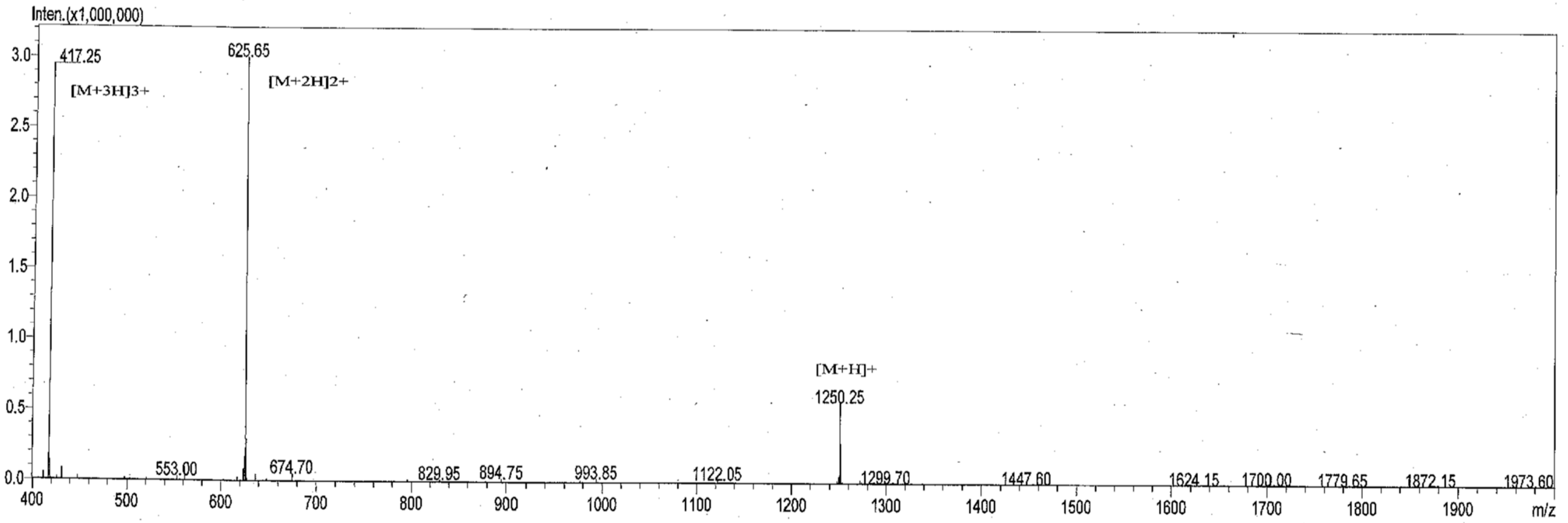

Sample Information		Probe:	ESI	Probe Bias:	+4.5kv
Injection Volume	: 1.00 μ l	Nebulizer Gas Flow:	1.5L/min	Detector:	1.5kv
Sample:	KK-10	CDL:	-20.0v	T. Flow:	0.2ml/min
M.W.:	1249.63	CDL Temp.:	250 °C	B. Conc.:	50%H2O/50%ACN
Lot. No.:	P220627-JQ1000387	Block Temp.:	200 °C		

Peptide: KSL
Lot n°: P220627-JQ1000621
Producer: ProteoGenix

HPLC REPORT

Sample:	KK-10
Analyst:	HCM
Lot. No.:	P220627-JQ1000621
Column:	4.6×250mm, SinoChrom ODS-BP
Solvent A	A: 0.1% Trifluoroacetic Acid in 100% Acetonitrile
Solvent B	B: 0.1% Trifluoroacetic Acid in 100% Water

Gradient:

	A	B
0.0min	15%	85%
25.0min	40%	60%
25.1min	100%	0%
30.0min	Stop	

Volume:	5μl
Wavelength:	220nm
Flow rate:	1.0ml/min

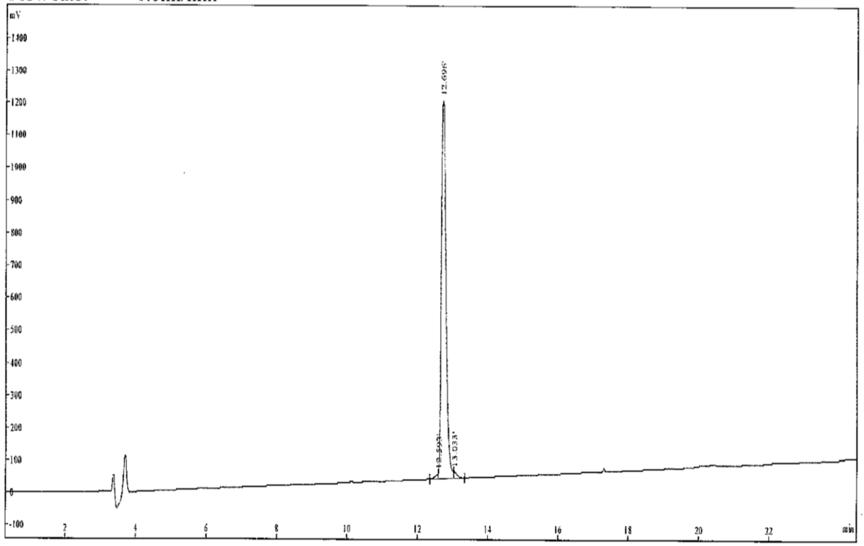

Rank	Time	Conc.	Area	Height
1	12.593	0.8751	85873	15457
2	12.696	98.17	9632902	1164184
3	13.033	0.9568	93892	18124
Total		100	9812667	1197765

MASS SPECTROMETRY REPORT

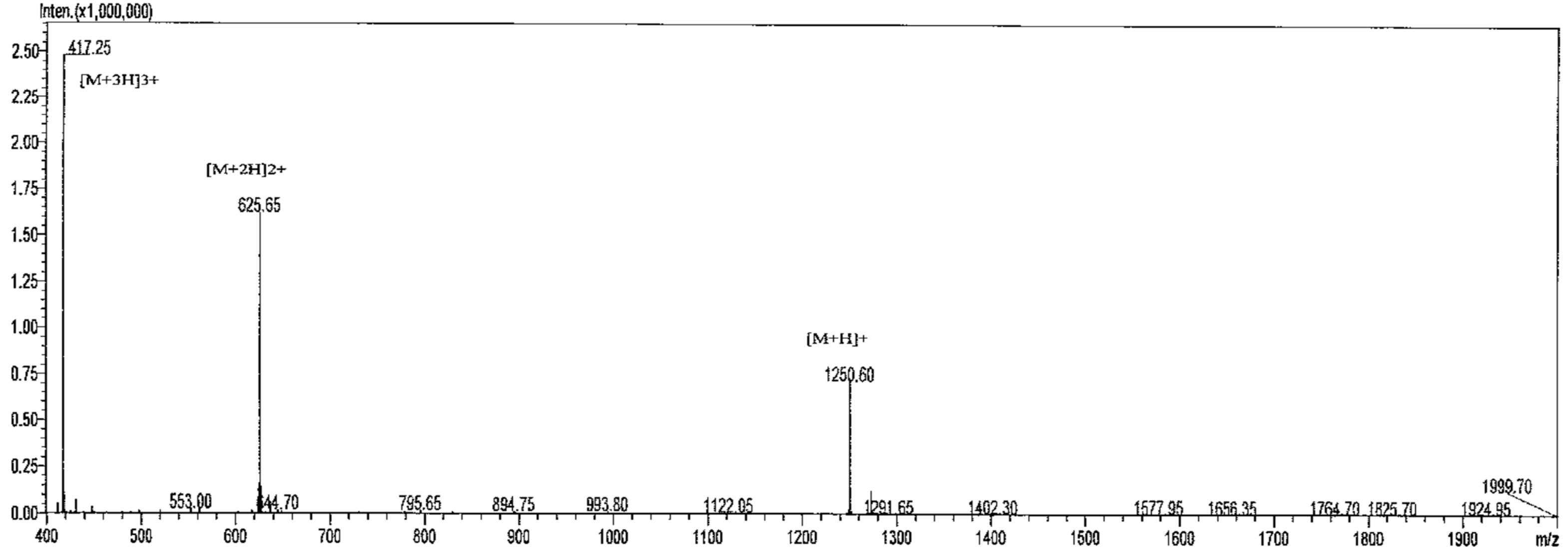

Sample Information		Probe:	ESI	Probe Bias:	+4.5kv
Injection Volume	: 1.00 µl	Nebulizer Gas Flow:	1.5L/min	Detector:	1.5kv
Sample:	KK-10	CDL:	-20.0v	T. Flow:	0.2ml/min
M.W.:	1249.63	CDL Temp.:	250°C	B. Conc.:	50%H2O/50%ACN
Lot. No.:	P220627-JQ1000621	Block Temp.:	200°C		

Peptide: KSL
Lot n°: P230327-MJ1066775
Producer: ProteoGenix

Sample: >KSL-10AA KK-10-NH2
Lot. No.: P230327-MJ1066775
Column: Kromasil 100-5C18, 4.6*250mm, 5μm
Solvent A A: 0.1% Trifluoroacetic Acid in 100% Acetonitrile
Solvent B B: 0.1% Trifluoroacetic Acid in 100% Water
Gradient:

	A	B
0.0min	13%	87%
25.0min	38%	62%
25.1min	100%	0%
30.0min	Stop	

Volume: 10μl
Wavelength: 220nm
Flow rate: 1.0ml/min

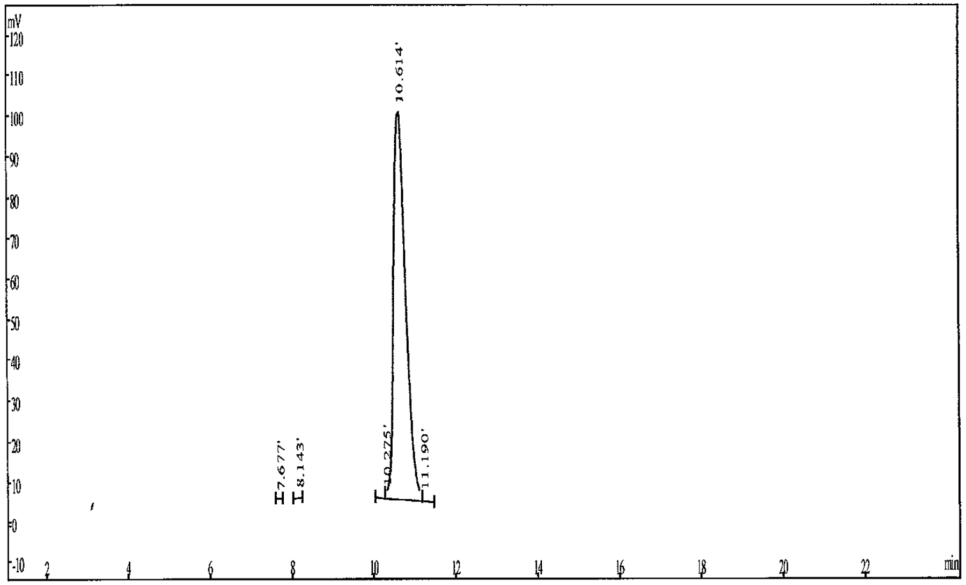

Rank	Time	Conc.	Area	Height
1	0.011	0.1835	3475	396
2	7.677	0.1177	2230	329
3	8.143	0.3494	6617	841
4	10.275	0.4285	8116	1061
5	10.614	98.33	1862321	95620
6	11.190	0.5905	11184	861
Total		100	1893943	99108

MASS SPECTROMETRY REPORT

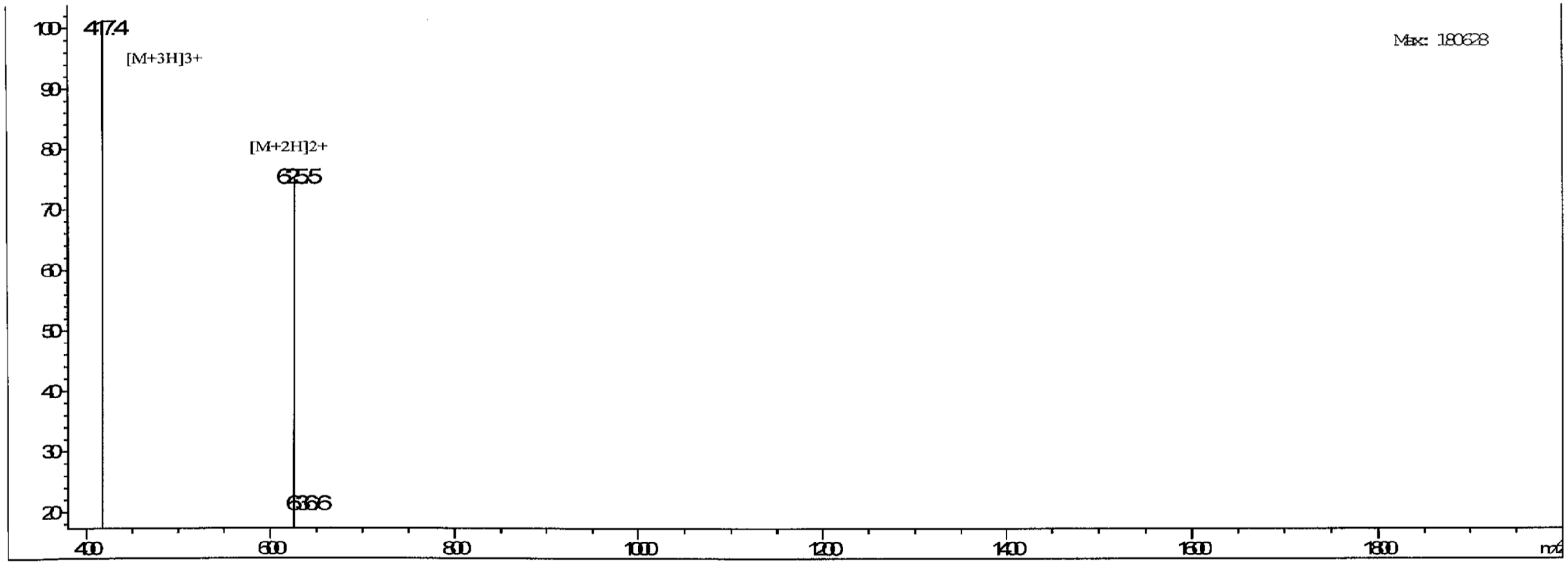

Sample Information		Probe:	ESI	Probe Bias:	+4.5kv
Injection Volume	: 1.00 µ l	Nebulizer Gas Flow:	1.5L/min	Detector:	1.5kv
Sample:	>KSL- 10 AA KK-10-NH2	CDL:	-20.0v	T. Flow:	0.2ml/min
M.W.:	1249.63	CDL Temp.:	250 °C	B. Conc.:	50%H2O/50%ACN
Lot. No.:	P230327-MJ1066775	Block Temp.:	200 °C		

Peptide: KSL-W
Lot n°: P220627-JQ1000388
Producer: ProteoGenix

HPLC REPORT

Sample: KK-10
Analyst: HCM
Lot. No.: P220627-JQ1000388
Column: 4.6×250mm, SinoChrom ODS-BP
Solvent A: A: 0.1% Trifluoroacetic Acid in 100% Acetonitrile
Solvent B: B: 0.1% Trifluoroacetic Acid in 100% Water
Gradient:

	A	B
0.0min	20%	80%
25.0min	45%	55%
25.1min	100%	0%
30.0min	Stop	

Volume: 5μl
Wavelength: 220nm
Flow rate: 1.0ml/min

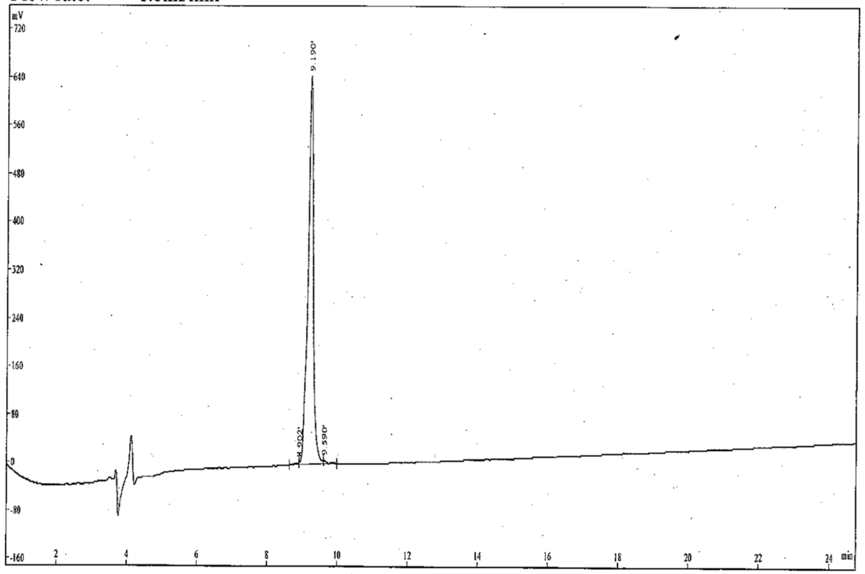

Rank	Time	Conc.	Area	Height
1	8.902	0.4391	28186	5615
2	9.190	98.52	6323560	644517
3	9.590	1.039	66679	6375
Total		100	6418425	656507

MASS SPECTROMETRY REPORT

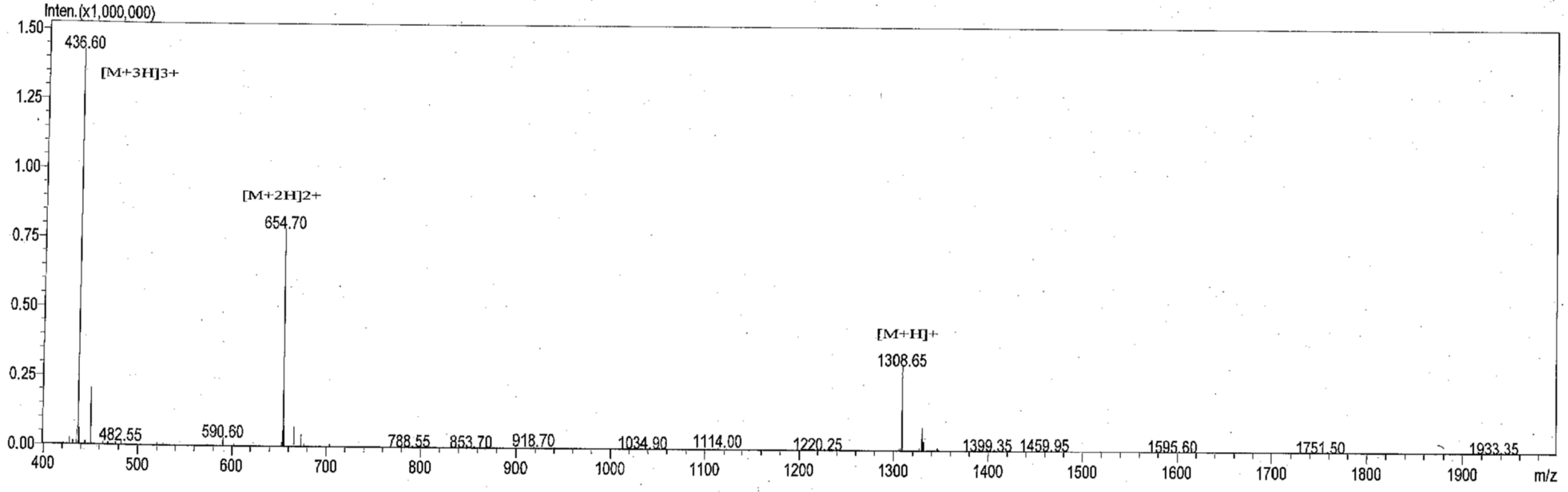

Sample Information					
Injection Volume	: 1.00 μ l	Probe:	ESI	Probe Bias:	+4.5kv
Sample:	KK-10	Nebulizer Gas Flow:	1.5L/min	Detector:	1.5kv
M.W.:	1307.67	CDL:	-20.0v	T. Flow:	0.2ml/min
Lot. No.:	P220627-JQ1000388	CDL Temp.:	250 °C	B. Conc.:	50%H2O/50%ACN
		Block Temp.:	200 °C		

Peptide: KSL-W
Lot n°: P220627-JQ1000622
Producer: ProteoGenix

HPLC REPORT

Sample: KK-10
Analyst: HCM
Lot. No.: P220627-JQ1000622
Column: 4.6×250mm, SinoChrom ODS-BP
Solvent A A: 0.1% Trifluoroacetic Acid in 100% Acetonitrile
Solvent B B: 0.1% Trifluoroacetic Acid in 100% Water
Gradient:

	A	B
0.0min	20%	80%
25.0min	45%	55%
25.1min	100%	0%
30.0min	Stop	

Volume: 5μl
Wavelength: 220nm
Flow rate: 1.0ml/min

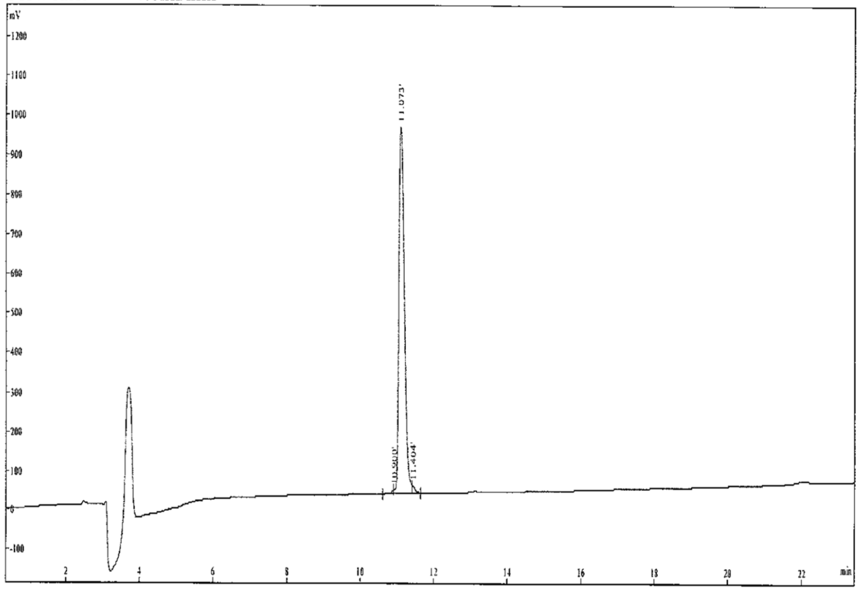

Rank	Time	Conc.	Area	Height
1	10.900	0.5047	44850	8792
2	11.073	98.79	8777242	926126
3	11.404	0.7101	63094	17810
Total		100	8885186	952728

MASS SPECTROMETRY REPORT

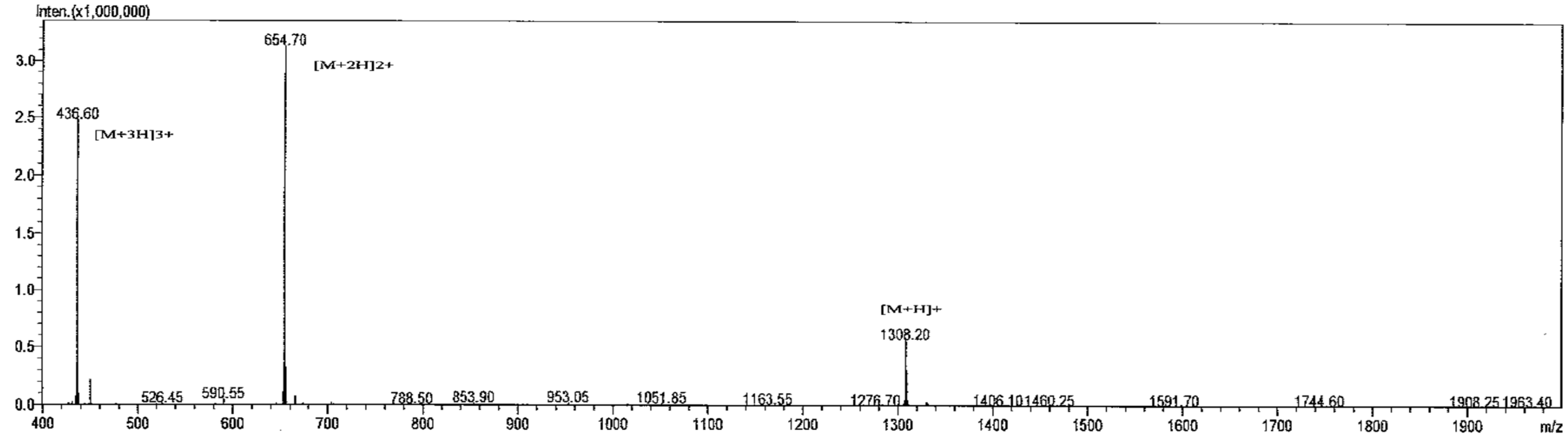

Sample Information		Probe:	ESI	Probe Bias:	+4.5kv
Injection Volume	: 1.00 μ l	Nebulizer Gas Flow:	1.5L/min	Detector:	1.5kv
Sample:	KK-10	CDL:	-20.0v	T. Flow:	0.2ml/min
M.W.:	1307.67	CDL Temp.:	250 °C	B. Conc.:	50%H2O/50%ACN
Lot. No.:	P220627-JQ1000622	Block Temp.:	200 °C		

Peptide: KSL-W
Lot n°: P230327-MJ1066774
Producer: ProteoGenix

Sample:	>KLS-W-10AA KK-10-NH2		
Lot. No.:	P230327-MJ1066774		
Column:	Boston Green 0DS-AQ, 4.6*250mm, 5μm		
Solvent A	A: 0.1% Trifluoroacetic Acid in 100% Acetonitrile		
Solvent B	B: 0.1% Trifluoroacetic Acid in 100% Water		
Gradient:		A	B
	0.0min	15%	85%
	25.0min	40%	60%
	25.1min	100%	0%
	30.0min	Stop	
Volume:	10μl		
Wavelength:	220nm		
Flow rate:	1.0ml/min		

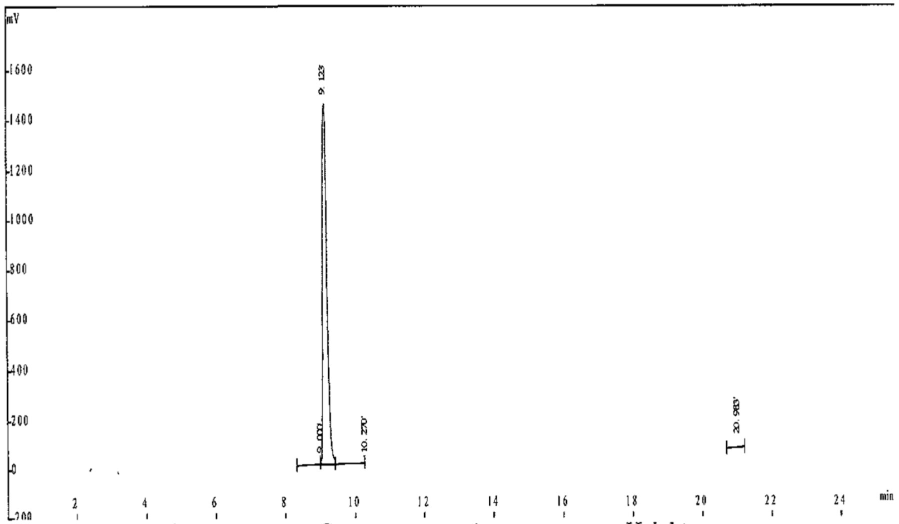

Rank	Time	Conc	Area	Height
1	9.000	0.2543	28069	5509
2	9.123	98.23	10841934	1444193
3	10.270	0.2263	24972	3
4	20.983	1.285	141796	15147
Total		100.0000		

MASS SPECTROMETRY REPORT

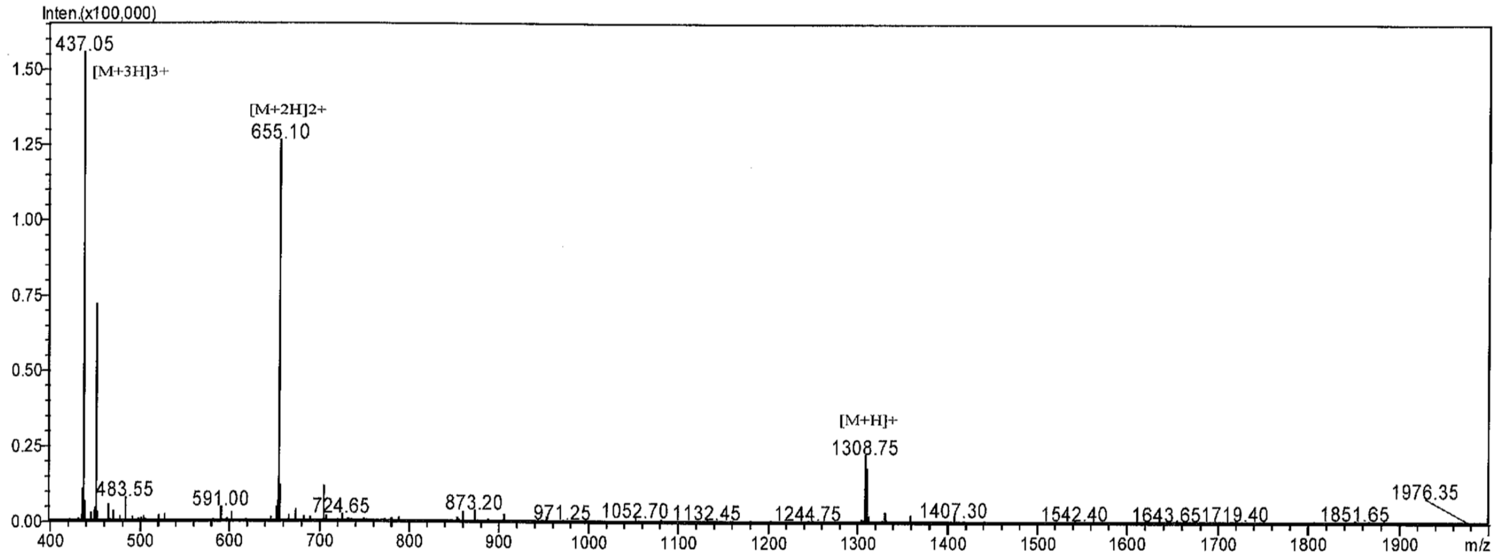

Sample Information

Injection Volume	: 1.00 μ l
Sample:	>KLS-W - 10 AA KK-10-NH2
M.W.:	1307.67
Lot. No.:	P230327-MJ1066774

Probe:	ESI	Probe Bias:	+4.5kv
Nebulizer Gas Flow:	1.5L/min	Detector:	1.5kv
CDL:	-20.0v	T. Flow:	0.2ml/min
CDL Temp.:	250 °C	B. Conc.:	50%H2O/50%ACN
Block Temp.:	200 °C		

Peptide: Dadapin-1
Lot n°: U2594588G0-1/PE8896
Producer: GenScript

Sample Name : Dadapin-1
Sample ID : U2594588G0-1
Time Processed :16:21:50
Month-Day-Year Processed :09/21/2023

Pump A : 0.065% trifluoroacetic in 100% water (v/v)
Pump B : 0.05% trifluoroacetic in 100% acetonitrile (v/v)
Total Flow:1 ml/min
Wavelength:220 nm
<<LC Time Program>>

Time	Module	Command	Value
0.01	Pumps	B.Conc	5
25.00	Pumps	B.Conc	65
25.01	Pumps	B.Conc	95
27.00	Pumps	B.Conc	95
27.01	Pumps	B.Conc	5
35.00	Pumps	B.Conc	5
35.01	Controller	Stop	

<<Column Performance>>
<Detector A>
Column :Inertsil ODS-SP 4.6 x 250 mm
Equipment: GR11010440

<Chromatogram>

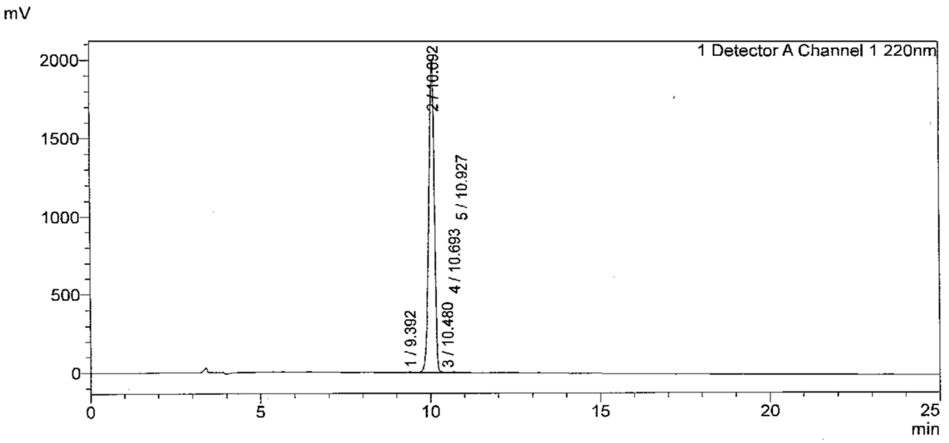

<Peak Table>

Detector A Channel 1 220nm

Peak#	Ret. Time	Area	Height	Area%
1	9.392	48793	4226	0.214
2	10.092	22656940	2008888	99.281
3	10.480	39052	5405	0.171
4	10.693	56168	6562	0.246
5	10.927	20082	1840	0.088
Total		22821035	2026921	100.000

Mass Spectrum

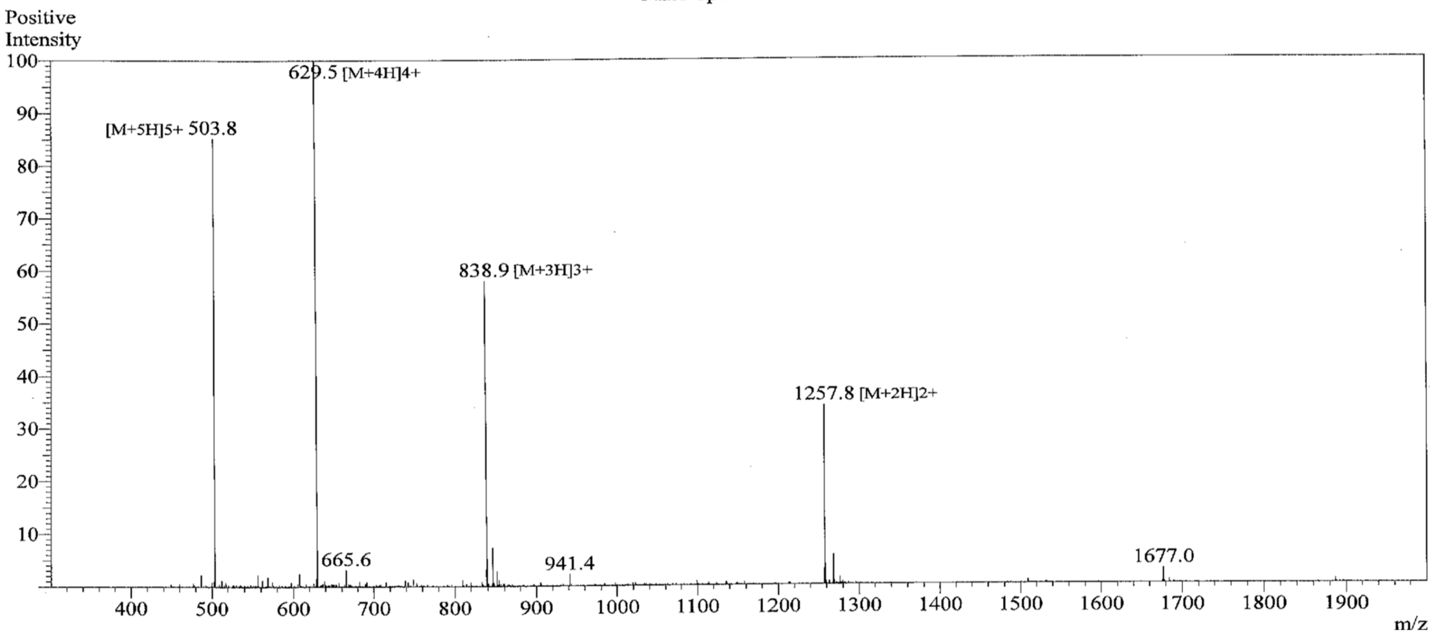

Sample Information
Month-Day Processed : 09/20/23
Time Processed : 22:00:21
Injection Volume : 0.1
Sample Name : Dadapin-1
Sample ID : U2594588G0-1
Theoretical MW : 2513.93
Observed MW : 2514.0

Interface :ESI
Nebulizing Gas Flow :1.5L/min
CDL Temp :250
Block Temp :200

Equipment :ZJ22010150
Interface Bias : +4.5 kV
Drying Gas Flow :5 L/min
T.Flow :0.2 ml/min
B.conc :50%H2O/50%MeOH

Peptide: KSL
Lot n°: U2594588G0-8/PE8903
Producer: GenScript

Sample Name :KSL
Sample ID :U2594588G0-8
Time Processed :23:24:05
Month-Day-Year Processed :07/29/2023

Pump A : 0.065% trifluoroacetic in 100% water (v/v)
Pump B : 0.05% trifluoroacetic in 100% acetonitrile (v/v)
Total Flow:1 ml/min
Wavelength:220 nm
<<LC Time Program>>

Time	Module	Command	Value
0.01	Pumps	B.Conc	5
25.00	Pumps	B.Conc	65
25.01	Pumps	B.Conc	95
27.00	Pumps	B.Conc	95
27.01	Pumps	B.Conc	5
35.00	Pumps	B.Conc	5
35.01	Controller	Stop	

<<Column Performance>>
<Detector A>
Column :Inertsil ODS-SP 4.6 x 250 mm
Equipment: GK12010012

<Chromatogram>

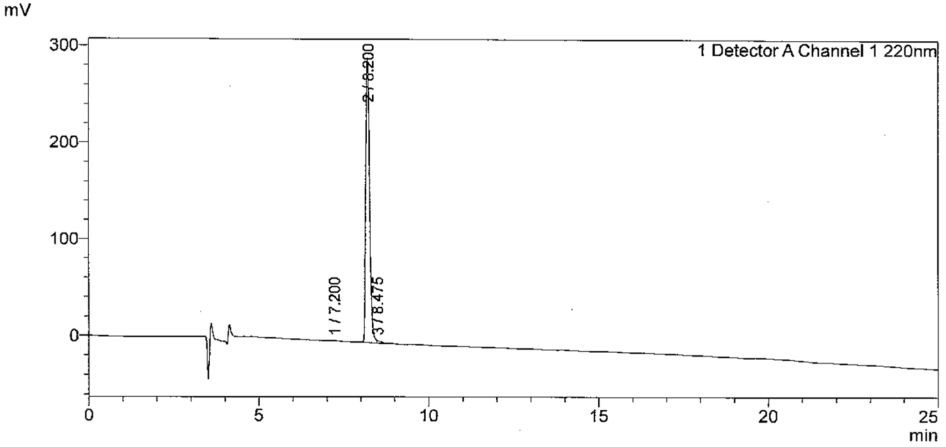

<Peak Table>

Detector A Channel 1 220nm

Peak#	Ret. Time	Area	Height	Area%
1	7.200	9472	1334	0.404
2	8.200	2302003	295255	98.300
3	8.475	30344	2967	1.296
Total		2341819	299556	100.000

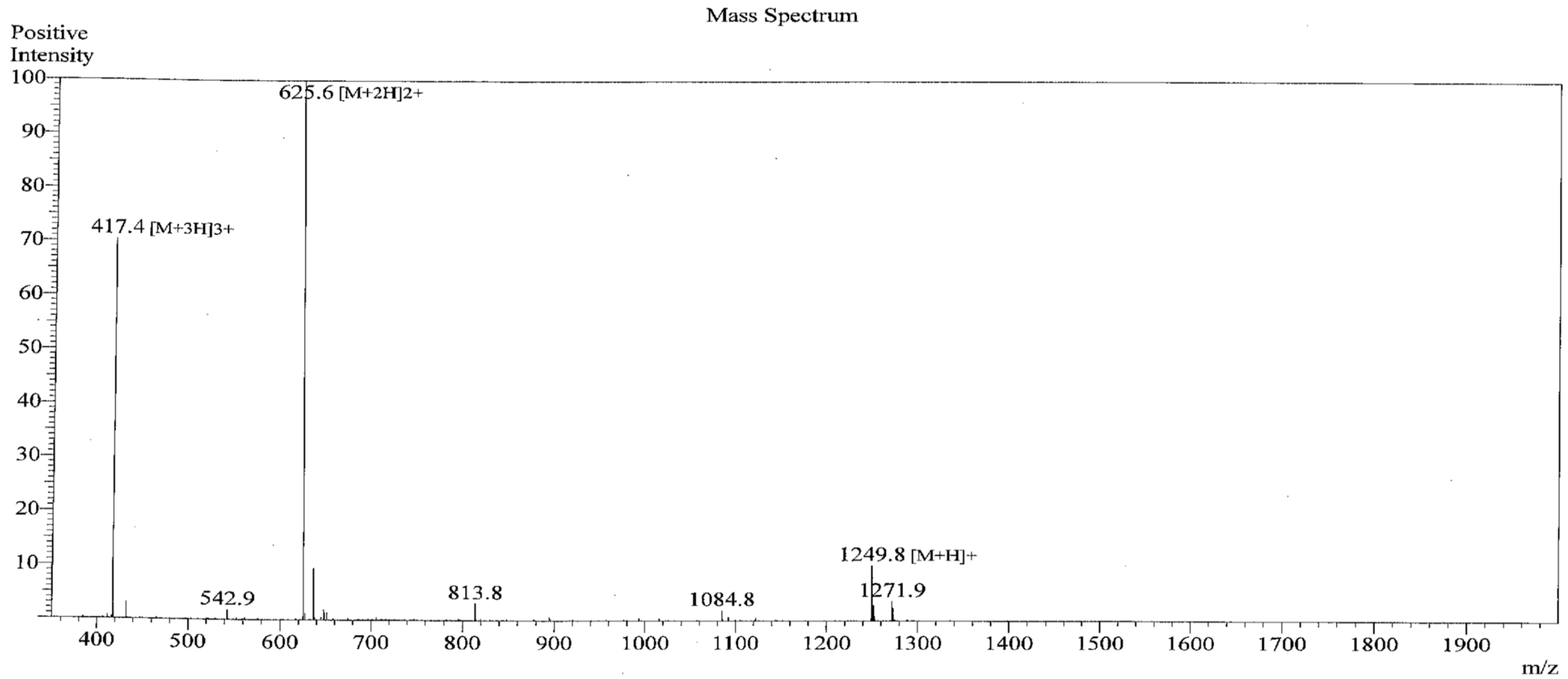

Sample Information

Month-Day Processed :	07/29/23
Time Processed :	19:13:10
Injection Volume :	0.3
Sample Name :	KSL
Sample ID :	U2594588G0-8
Theoretical MW :	1249.64
Observed MW :	1249.2

Interface	:ESI
Nebulizing Gas Flow	:1.5L/min
CDL Temp	:250
Block Temp	:200

Equipment	: ZJ21010035
Interface Bias	: +4.5 kV
Drying Gas Flow	:5 L/min
T.Flow	:0.2 ml/min
B.conc	:50%H2O/50%MeOH

Peptide: KSL-W
Lot n°: U2594588G0-10/PE8905
Producer: GenScript

Sample Name :KLS-W
Sample ID :U2594588G0-10
Time Processed :6:15:54
Month-Day-Year Processed :07/29/2023

Pump A : 0.065% trifluoroacetic in 100% water (v/v)
Pump B : 0.05% trifluoroacetic in 100% acetonitrile (v/v)
Total Flow:1 ml/min
Wavelength:220 nm
<<LC Time Program>>

Time	Module	Command	Value
0.01	Pumps	B.Conc	5
25.00	Pumps	B.Conc	65
25.01	Pumps	B.Conc	95
27.00	Pumps	B.Conc	95
27.01	Pumps	B.Conc	5
35.00	Pumps	B.Conc	5
35.01	Controller	Stop	

<<Column Performance>>
<Detector A>
Column :Inertsil ODS-SP 4.6 x 250 mm
Equipment: ZJ20010140

<Chromatogram>

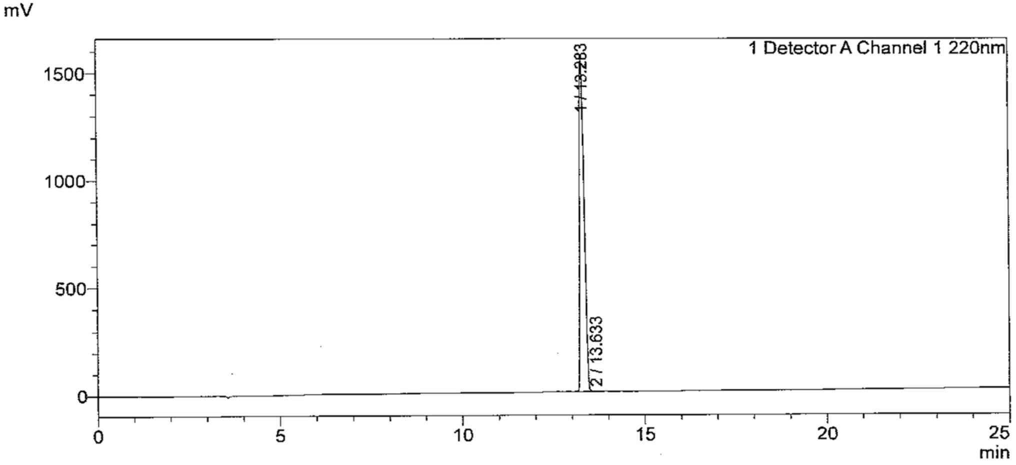

<Peak Table>

Detector A Channel 1 220nm

Peak#	Ret. Time	Area	Height	Area%
1	13.283	13778339	1555024	99.831
2	13.633	23272	3652	0.169
Total		13801611	1558675	100.000

Mass Spectrum

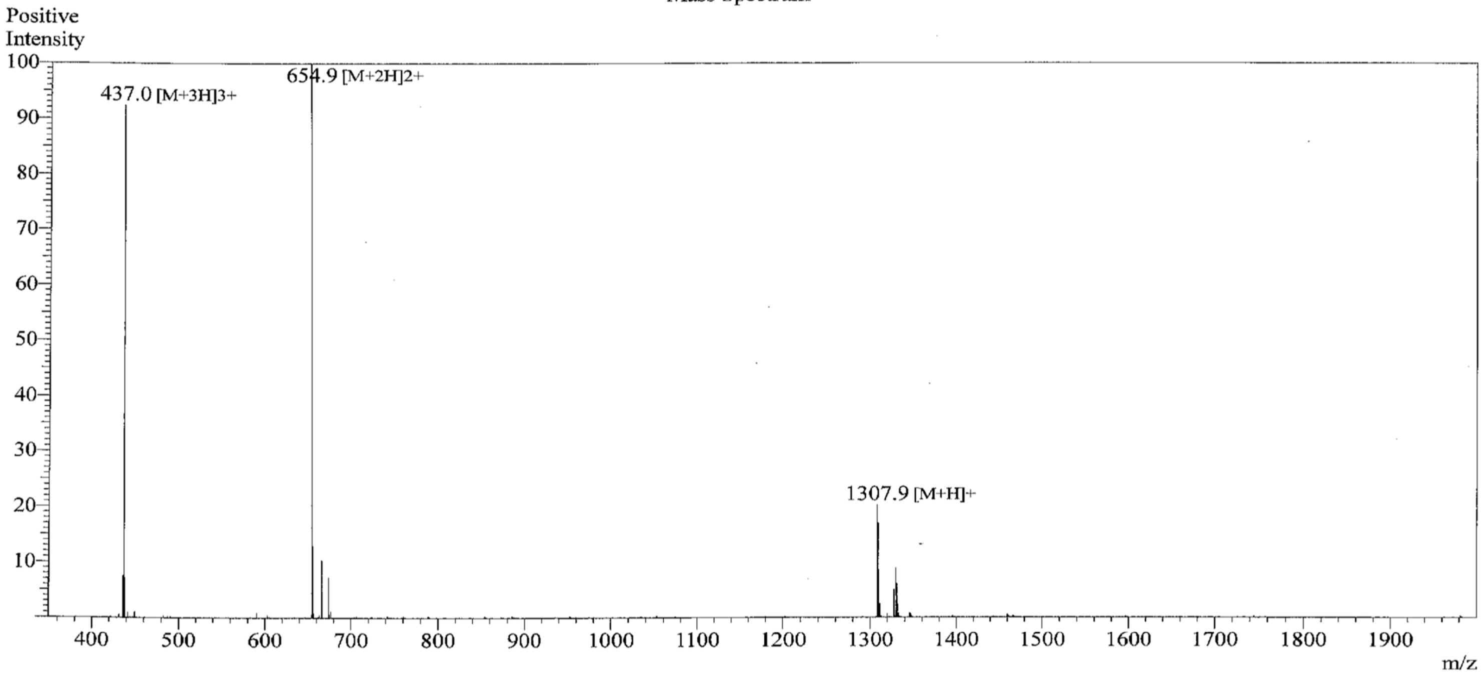

Sample Information
Month-Day Processed : 07/28/23
Time Processed : 22:04:37
Injection Volume : 0.3
Sample Name : KLS-W
Sample ID : U2594588G0-10
Theoretical MW : 1307.68
Observed MW : 1307.8

Interface :ESI
Nebulizing Gas Flow :1.5L/min
CDL Temp :250
Block Temp :200

Equipment : ZJ21010035
Interface Bias : +4.5 kV
Drying Gas Flow :5 L/min
T.Flow :0.2 ml/min
B.conc :50%H2O/50%MeOH
